# The Wnt-specific astacin proteinase HAS-7 restricts head organizer formation in *Hydra*

**DOI:** 10.1186/s12915-021-01046-9

**Published:** 2021-06-09

**Authors:** Berenice Ziegler, Irene Yiallouros, Benjamin Trageser, Sumit Kumar, Moritz Mercker, Svenja Kling, Maike Fath, Uwe Warnken, Martina Schnölzer, Thomas W. Holstein, Markus Hartl, Anna Marciniak-Czochra, Jörg Stetefeld, Walter Stöcker, Suat Özbek

**Affiliations:** 1 Centre for Organismal Studies, Department of Molecular Evolution and Genomics, University of Heidelberg, Im Neuenheimer Feld 230, 69120 Heidelberg, Germany; 2 Institute of Molecular Physiology, Cell and Matrix Biology, Johannes Gutenberg University Mainz, 55099 Mainz, Germany; 3 Department of Cell and Chemical Biology, Leiden University Medical Center, Leiden, Netherlands; 4 Institute for Applied Mathematics, Interdisciplinary Center for Scientific Computing, Heidelberg University, Im Neuenheimer Feld 205, 69120 Heidelberg, Germany; 5 Functional Proteome Analysis, German Cancer Research Center (DKFZ), Heidelberg, Germany; 6 Institute of Biochemistry and Center for Molecular Biosciences, University of Innsbruck, Innrain 80-82, A-6020 Innsbruck, Austria; 7 Department of Chemistry, University of Manitoba, 144 Dysart Road, Winnipeg, Manitoba R3T 2 N2 Canada

**Keywords:** Hydra, Wnt signaling, Proteinase, Astacin, Axis formation

## Abstract

**Background:**

The *Hydra* head organizer acts as a signaling center that initiates and maintains the primary body axis in steady state polyps and during budding or regeneration. Wnt/beta-Catenin signaling functions as a primary cue controlling this process, but how Wnt ligand activity is locally restricted at the protein level is poorly understood. Here we report a proteomic analysis of *Hydra* head tissue leading to the identification of an astacin family proteinase as a Wnt processing factor.

**Results:**

*Hydra* astacin-7 (HAS-7) is expressed from gland cells as an apical-distal gradient in the body column, peaking close beneath the tentacle zone. *HAS-7* siRNA knockdown abrogates HyWnt3 proteolysis in the head tissue and induces a robust double axis phenotype, which is rescued by simultaneous *HyWnt3* knockdown. Accordingly, double axes are also observed in conditions of increased Wnt activity as in transgenic actin::HyWnt3 and *HyDkk1/2/4* siRNA treated animals. HyWnt3-induced double axes in *Xenopus* embryos could be rescued by coinjection of *HAS-7* mRNA. Mathematical modelling combined with experimental promotor analysis indicate an indirect regulation of *HAS-7* by beta-Catenin, expanding the classical Turing-type activator-inhibitor model.

**Conclusions:**

We show the astacin family protease HAS-7 maintains a single head organizer through proteolysis of HyWnt3. Our data suggest a negative regulatory function of Wnt processing astacin proteinases in the global patterning of the oral-aboral axis in *Hydra*.

**Supplementary Information:**

The online version contains supplementary material available at 10.1186/s12915-021-01046-9.

## Background

**Fig. 1:**
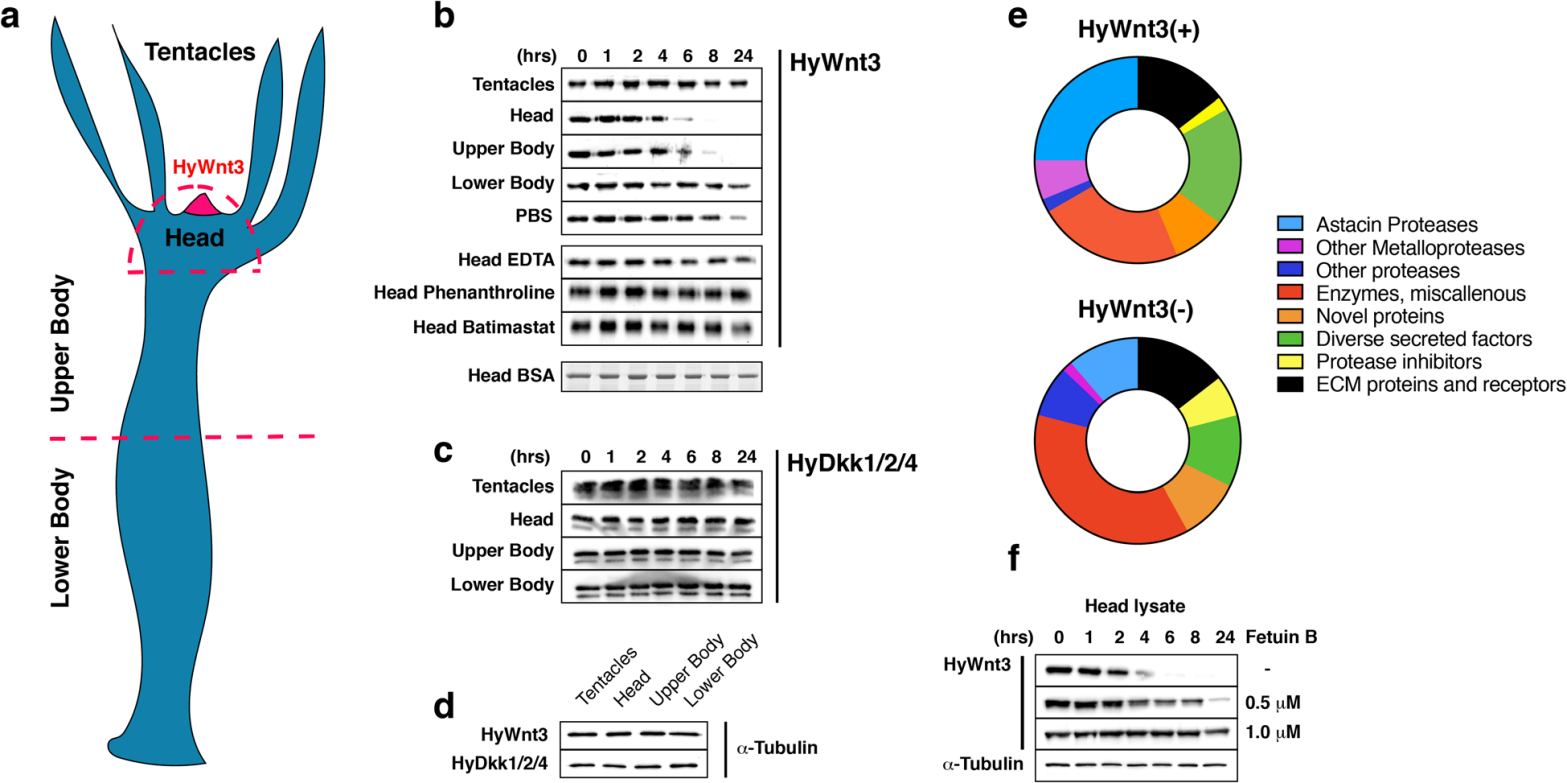
Screen for HyWnt3 proteolytic activity in *Hydra* tissue lysates. **a** Schematic representation of the *Hydra* body plan. Body parts used for lysates in **b**–**d** are indicated. The hypostomal organizer, which harbors *HyWnt3* expressing cells is marked in red. **b** Recombinant HyWnt3-His levels, monitored by Western blotting with anti-His antibody, were reduced after ~ 4 h incubation in the head lysate and after ~ 6 h in the upper body lysate. No cleavage was observed during incubation in tentacle and lower body lysates, while incubation in the PBS control showed unspecific cleavage at 24 h. No unspecific proteolysis of 1 μg BSA was detectable in HL over the time period of 24 h as detected by SDS-PAGE and Coomassie staining. HyWnt3-His cleavage activity in HL was completely blocked by the addition of broad zinc metalloproteinase inhibitors EDTA and Phenanthroline or the matrix metalloproteinase inhibitor Batimastat. **c** No cleavage was observed for the recombinant Wnt antagonist HyDkk1/2/4-His in the respective body tissue lysates during a 24-h incubation time. Mark that the double band appearance is an SDS-PAGE artifact. **d** Tissue lysates from different body parts of adult hydra polyps as indicated in the scheme were adjusted in total protein concentrations by tubulin Western blotting. **e** Distribution of protein classes in the *Hydra* HL secretome identified in HyWnt3(+) and HyWnt3(−) fractions as indicated. The full dataset is given in Additional file 2: Table S1a-b. **f** HyWnt3-His processing is inhibited by recombinant mouse Fetuin-B protein in a dose-dependent manner as indicated

Wnt signaling promotes primary axis development in diverse phyla across the animal kingdom [1–3]. The role of Wnt/beta-Catenin signaling in the axial patterning of cnidarians has been extensively studied in the freshwater polyp *Hydra* [4–7], which has a single oral-aboral body axis. The head is separated from the gastric region by a ring of tentacles and runs out at the upper part into a cone-shaped mouth region, called the hypostome. At its apical tip, the hypostome contains the head organizer [8], comprising a small cluster of ecto- and endodermal cells that continuously express *HyWnt3* in steady state polyps (Fig. 1a) [9]. *HyWnt3* is upregulated early during head regeneration and has been shown to initiate a cascade of Wnt signaling events directing axial patterning [7]. While the spatially restricted HyWnt3 ligand production is controlled at the transcriptional level by repressive elements in the *HyWnt3* promotor region [9, 10], it is poorly understood how Wnt activity is regulated at protein level in the extracellular space. In *Hydra*, only a member of the Dkk1/2/4 family of secreted Wnt inhibitors has so far been shown to function as a Wnt antagonist by creating a Wnt-suppressed region in the body column [11]. Recently, we have shown that the matricellular protein Thrombospondin (HmTSP) is expressed directly from or in close vicinity of *HyWnt3* expressing cells of the hypostome and exerts a negative regulatory function on organizer formation [12]. It is unclear, though, whether HmTSP interacts directly with Wnt ligands or modulates Wnt inactivity by influencing receptor mobility or turnover.

Morphogen activity during embryogenesis can also be restricted by proteinases that process secreted ligands. A prominent example is the zinc metalloproteinase BMP1 and its splice variant mammalian Tolloid (i.e. Xolloid in *Xenopus*), which specifically cleaves Chordin and thus promotes local BMP signaling at the ventral side of the vertebrate embryo [13]. A similar case for morphogen inactivation has been proposed for TIKI1, a highly conserved metalloproteinase expressed in the *Xenopus* organizer and shown to antagonize Wnt function by cleaving eight amino-terminal residues of Wnt3a [14].

In *Hydra*, functional studies on astacin metalloproteinases have indicated important roles in processes of morphogenesis and regeneration [15–17]. Yan et al. have shown that the metalloproteinase HMP1 is localized to the head pole and that an anti-HMP1 antibody can effectively block head regeneration [17]. HMP2, a *Hydra* astacin proteinase containing a MAM-domain, which is typical for meprin-like enzymes of the astacin-family, formed an opposing gradient to HMP1, showing the highest expression at the basal pole of the animal [16]. Although different mechanistic pathways as the proteolytic activation of morphogens or regulatory peptides have been discussed in these studies, no detailed molecular mechanisms comparable to those for Tolloid or TIKI1 have been described so far for any cnidarian metalloproteinase.

Here, we identify a member of the astacin proteinase family in *Hydra* with Wnt3 processing activity. *Hydra* Astacin-7 (HAS-7) is expressed in an increasing gradient towards the tentacle base of the polyp, forming a ring-like zone between head and body column that shows upregulated expression for several other members of the astacin family. siRNA knockdown of *HAS-7* eliminates the HyWnt3 proteolytic activity of the head tissue leading to a robust double-axis phenotype with a fully developed head structure. In addition, *HAS-7* mRNA injection into *Xenopus* embryos rescues double axes induced by *HyWnt3* mRNA. Our combined experimental data and mathematical models demonstrate a direct mechanistic link between astacin proteinases and Wnt-regulated pattern formation in *Hydra* by restricting Wnt ligand activity to the head region via specific proteolysis.

## Results and discussion

### Identification of HyWnt3 proteolytic activity in the *Hydra* head lysate

To identify factors restricting Wnt activity in the extracellular space, we first examined the protein stability of recombinant HyWnt3-His in tissue lysates generated from different body parts of *Hydra* (Fig. 1a). For this, lysates of the head region, tentacles, upper and lower body trunk were prepared and their soluble fractions were adjusted in total protein concentrations to 4 mg/ml. ~ 10 ng of purified recombinant HyWnt3-His protein was incubated with equal amounts of each tissue lysate and then the reaction was stopped after different time points. Detection by Western blotting localized the highest proteolytic activity for HyWnt3-His in the head lysate (reduced after ~ 4 h) and, to a lesser extent, in the upper body lysate (reduced after ~ 6 h) (Fig. 1b, d). While unspecific proteolysis of HyWnt3-His was evident after 24 h incubation in PBS, the recombinant protein stayed remarkably stable in lysates of tentacles and the lower body part. Incubation of 1 μg BSA in the head lysate (HL) did not show unspecific proteolysis over the given time period of 24 h (Fig. 1b). In HL samples supplemented with broad-spectrum metalloproteinase inhibitors like EDTA and 1,10-Phenanthroline or the specific matrix metalloproteinase inhibitor Batimastat, HyWnt3-His processing was completely blocked in the given time frame, indicating that metalloproteinases could be responsible for the observed activity. A parallel experiment performed with recombinantly expressed HyDkk1/2/4-His protein, a major Wnt antagonist [11], showed no specific proteolytic activity targeting this factor when it was incubated with the respective lysates (Fig. 1c, d).

To isolate candidate factors involved in HyWnt3 processing we next used a proteomic approach. A pool of HL from 200 polyps was fractionated by cation exchange chromatography (Additional file 1: Fig. S1a) and peak fractions were re-examined for their HyWnt3-His processing activity applying a 6-h incubation time (Additional file 1: Fig. S1b). A fragment encompassing the two N-terminal cadherin domains of *Hydra* cadherin [18] was used as a control substrate to monitor general matrix metalloproteinase activity. We observed complete HyWnt3-His cleavage using fractions 1–5, while *Hydra* cadherin was degraded partially by fractions 2 and 3. To exclude a high background of possibly unspecific proteinases in fractions 1-3 we pooled fractions 4–5 (HyWnt3(+)) and 6-7 (HyWnt3(−)) for further analysis and performed orbitrap mass spectrometry analysis after in-solution digestion of the respective pooled samples. When we filtered the obtained protein hits for unique sequences of proteins having a signal peptide for secretion and at least two peptide hits, astacin family proteinases constituted the largest group in the HyWnt3(+) secretome whereas miscellaneous enzymes dominated in the HyWnt3(−) fraction (Fig. 1e, Additional file 2: Table S1a-b, Additional file 3: Table S2). Of the 12 astacin sequences detected in the HyWnt3(+) fraction, five were also present in the HyWnt3(−) fraction, although with lower protein scores. The HyWnt3(−) secretome additionally contained an increased number of proteinases belonging to diverse families (Fig. 1e, Additional file 2: Table S1b, Additional file 3: Table S2). We concluded from these results that metalloproteinases, in particular astacin-type proteinases, are likely candidates for the observed HyWnt3-His processing activity. To confirm this notion, we tested the proteolytic activity of HL on HyWnt3-His in the presence of recombinant mammalian Fetuin-B, which was recently shown to function as a highly specific physiological inhibitor of astacin-type proteinases like ovastacin [19]. As shown in Fig. 1f, murine Fetuin-B blocked HyWnt3-His processing by HL in a dose-dependent manner.

### Characterization of the HyWnt3(+) astacin secretome

**Fig. 2:**
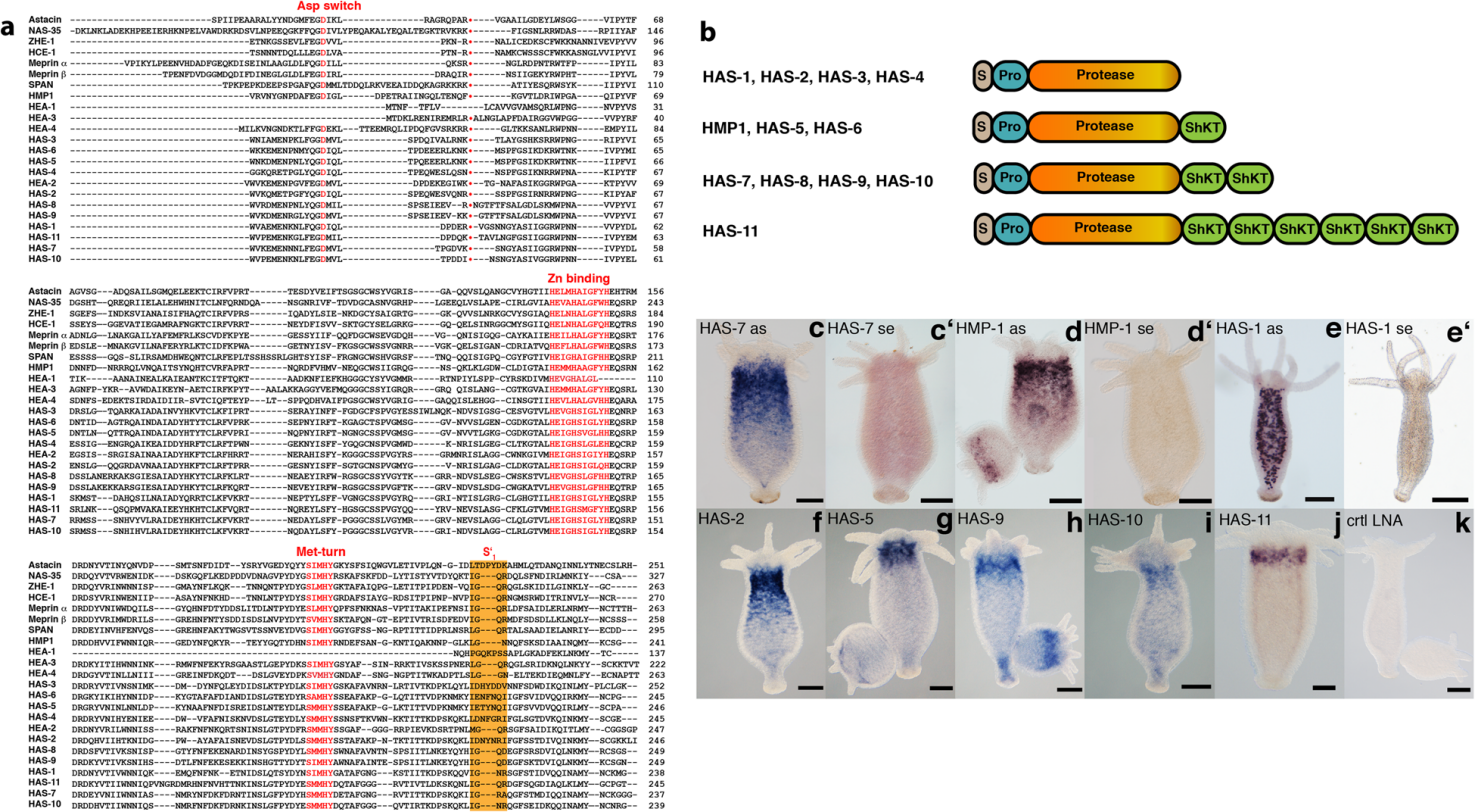
Sequence features and expression patterns of HyWnt3(+) astacin genes. **a** Multiple sequence alignment of pro-domain and catalytic domain sequences of astacins identified in this study. For comparison, astacin sequences from diverse species outside the cnidarian phylum were included. Gene ID numbers are as follows: Astacin *A. astacus* (P07584), NAS-35 *C. elegans* (P98060), ZHE-1 *Danio rerio* (Q1LW01), HCE-1 *O. latipes* (P31580), Meprin α *H. sapiens* (Q16819), Meprin β *H. sapiens* (Q16820), SPAN *S. purpuratus* (P98068), HMP1 (NP_001296695.1), HEA-1 *Hydractinia echinata* astacin 1 (Q2MCX9), HEA-3 *Hydractinia echinata* astacin 3 (Q2MCX7), HEA-4 *Hydractinia echinate* astacin 4 (Q2MCX6), HEA-2 *Hydractinia echinata* astacin 2 (Q2MCX8), HAS-3 (XP_002166229.3), HAS-6 (XP_002157397.2), HAS-5 (XP_002164800.1), HAS-4 (XP_002162738.1), HAS-2 (XP_002162822.1), HAS-8 (XP_002153855.1), HAS-9 (XP_002161766.1), HAS-1 (XP_012565441.1), HAS-11 (XP_012561076.1), HAS-7 (XP_012560086.1), HAS-10 (XP_002159980.2). In red: the aspartate switch residue in the pro-peptide, the zinc-binding motif, and the Met-turn. Orange background: residues forming the S1 ′ sub-site. A red bullet denotes the activation site. **b** Domain structures of astacins detected in HyWnt3(+) head lysate fractions. S, signal peptide; Pro, pro-domain; ShKT, *Stichodactyla* toxin domain. **c**–**k** WISH experiments using antisense and sense oligonucleotide probes (**c**–**e**) or LNAs (**f**–**k**) show a collar-like expression pattern marking a transition zone between head and body column for the majority of astacin genes. Representative of 10 hydras examined. Scale bars: HAS-7, 200 μm; HMP-1, 200 μm; HAS-1, 200 μm; HAS-2, 100 μm; HAS-11, 50 μm; HAS-5, 100 μm; HAS-9, 100 μm; HAS-10, 200 μm, control LNA, 200 μm

The HL HyWnt3(+) secretome contained 12 unique astacin sequences (hence called *Hydra* Astacins, HAS) with HAS-1 and HAS-7 showing the highest protein scores in the orbitrap mass spectrometry analysis (Additional file 2: Table S1a, Additional file 3: Table S2). The alignment of the pro- and catalytic domains with known astacin proteinase amino acid sequences demonstrated a high conservation of critical sequence motifs as the aspartate switch residue, methionine turn, and zinc binding motif (Fig. 2a). The domain structure of astacins comprises a signal peptide and a variable pro-domain segment, which is cleaved to activate the central ~ 200-residue catalytic domain (Fig. 2a, b). Typical for cnidarian astacins is the possession of C-terminal ShKT (*Stichodactyla* toxin) domains [20]. The majority of the astacins detected in our analysis comprises 1-2 ShKT domains, but several lack a C-terminal segment (Fig. 2b). HAS-11 is exceptional in possessing six ShKT domains in a tandem repeat. None of the astacin sequences was predicted to possess a transmembrane domain. A phylogenetic analysis places the *Hydra* astacins HAS-1-11 in a clade together with *Podocoryne carnea* PMP1 [21] and *Hydractinia echinata* astacin HEA2 [22] with high similarity to meprins (Additional file 4: Fig. S2). HMP1 forms a distantly related clade together with *Hydractinia* astacins HEA1, HEA3 and HEA4.

To be able to correlate the observed HyWnt3-specific proteolytic activity in the head and upper body tissues with expression patterns of the respective genes, we performed *in situ* hybridization experiments in whole mounts (WISH). Conventional WISH using mRNA antisense probes and sense controls was performed for the genes encoding the two highest-scoring protein hits, *HAS-1* and *HAS-7*, as well as for the previously described *HMP1* [16]. For the remaining astacin genes of the Wnt3(+) fraction, synthetic locked nucleic acid (LNA) probes [23] were used that have an increased target specificity for highly similar gene sequences. The majority of astacin genes showed an endodermal expression pattern in the upper half of the body trunk, often as a gradient that increased towards the head with a sharp boundary beneath the tentacle formation zone (*HAS-2*, *HAS-7*, *HMP1*, Fig. 2c, d, f), reminiscent of the *SEC-1* positive gland cell subpopulation described by Schmidt & David [24], and of the expression patterns of HEA1 and HEA2 in gland cells of metamorphosing *Hydractinia* polyps [22]. Several astacins (*HAS-5*, *HAS-9-11*) were expressed in a narrow collar demarcating the body region from the head (Fig. 2g–j). Four of the examined genes did not yield a reproducible staining pattern by WISH. *HAS-1* was exceptional in exhibiting a distinct expression in large gland cells in the endoderm that were homogenously distributed along the entire body column (Fig. 2e). This pattern is reminiscent of the Wnt antagonist *HyDkk1/2/4*, which is expressed by a subpopulation of zymogen gland cells [11]. By tracing the astacin genes of the HyWnt3(+) fraction in the *Hydra* single-cell transcriptome database [25] we were able to identify cell-specific expression signatures for each sequence (Additional file 5: Fig. S3a-b). Scatter plots showing the expression profiles along cell types and differentiation trajectories clearly identify i-cell-derived gland cells distributed mostly along the transition zone between head and upper body (zymogen to mucous gland cell transition) as the source of all examined astacins, except *HAS-1*, which was restricted to the zymogen gland cells of the body column. A close-up view of the epithelial layers in the upper gastric region of a *HAS-7* WISH sample confirmed that the mRNA signal is not present in the endodermal epithelial cells closely aligning the central line of the extracellular matrix, called mesoglea (Additional file 5: Fig. S3c). Gland cells in *Hydra* are interspersed singly between the endodermal epithelial cells without direct contact to the mesoglea [24].

In summary, the candidate astacin proteinases identified in our analysis are a product of endodermal i-cell derived gland cells with an overlapping peak expression beneath the head and tentacle region. We focused our further analysis on HAS-7 due to its high protein score in the mass spectrometry analysis and the graded expression pattern reflecting the diminishing HyWnt3-His proteolysis from head to lower body (Fig. 1a). Also, five of the astacins identified in the HyWnt3(+) HL fraction were also contained in the HyWnt3(-) fraction (HAS-1-3, HAS-6, HAS-9), indicating that they are unlikely candidates for acting as HyWnt3-specific proteinases.

### *HAS-7* siRNA knockdown induces double axis formation

**Fig. 3:**
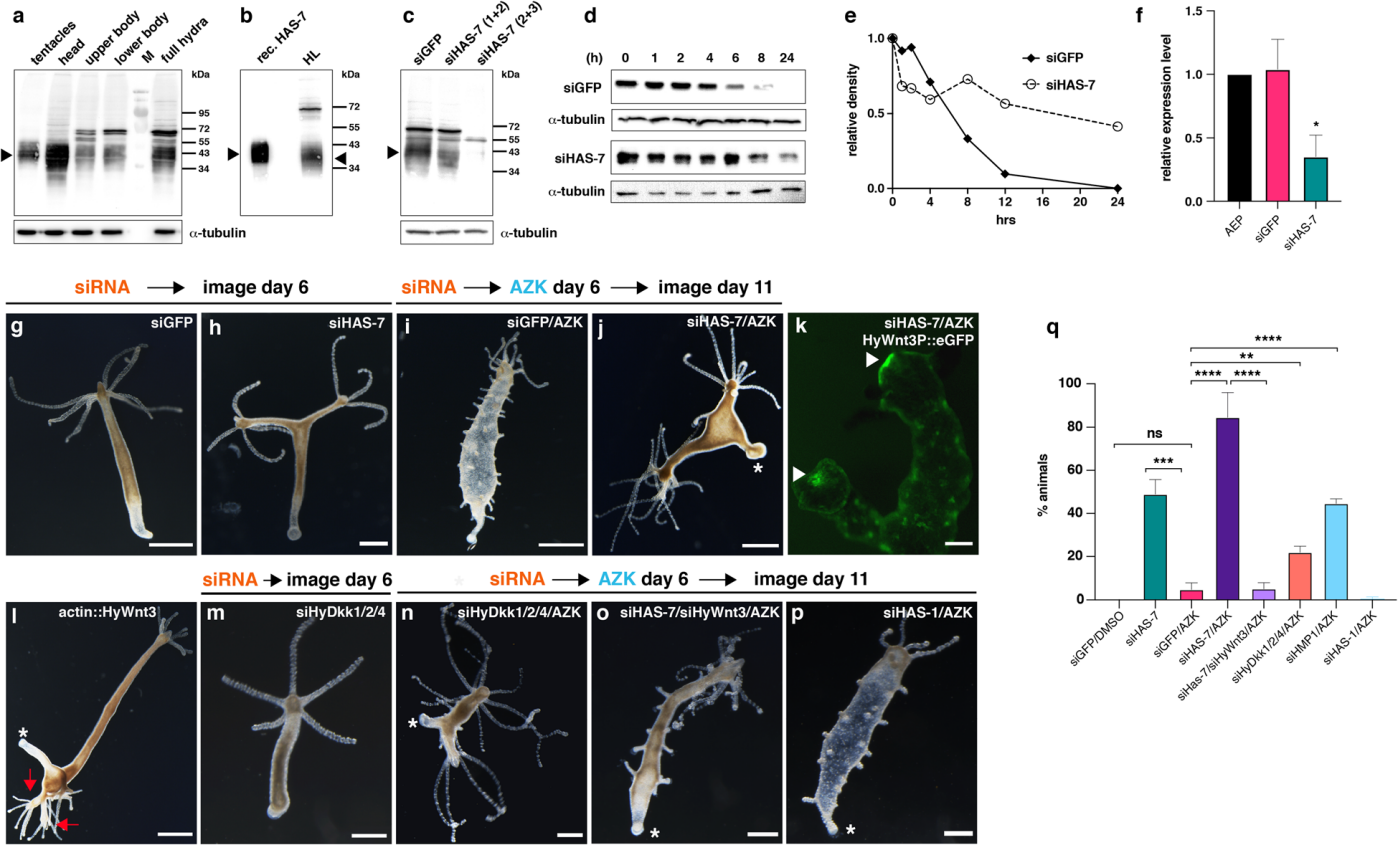
Functional analysis of *HAS-7* knockdown. **a** Detection of HAS-7 protein in body lysate samples using a polyclonal HAS-7 antibody. M = protein marker. Tubulin was used as loading control of the respective hydra lysates. The arrow indicates the position of the HAS-7 protein band, also in **b** and **c**. **b** Recombinant HAS-7 protein expressed in High Five cells compared to native protein in HL as detected by a HAS-7-specific antibody. Note that the slightly higher apparent molecular mass is due to the introduced histidine tag of recombinant HAS-7. **c** Knockdown effect of different siRNA combinations on HAS-7 protein levels was assayed by anti-HAS-7 Western blot analysis of complete hydras treated as indicated. Tubulin was used as loading control of the respective hydra lysates. The distinct band at 70 k Da in a-c likely represents a dimer of processed HAS-7. **d** HyWnt3-His proteolysis is impaired in HL of animals electroporated with siHAS-7 (2 + 3) as compared to siGFP control animals. Head lysates were generated 6 days after electroporation. Tubulin was used as loading control of the respective HL applied for each time point. **e** Relative intensities of the Western blot bands in **d**. **f** Quantitative real-time PCR analysis of HAS-7 expression in head tissues confirms the decreased expression in siHAS-7 treated animals compared to siGFP treated and untreated (steady-state AEP animals) controls. Relative expression levels are given in 2^(-ΔΔCt)^. Results represent mean +/− S.D. from 3 independent experiments, analyzed by *t* tests. *p < 0.05. The individual data values are shown in Additional file 14. **g** siGFP control electroporation without AZK treatment shows normal morphology. Scale bar = 500 μm. **h** siHAS-7 electroporation without AZK treatment shows animals with double axis lacking ectopic tentacles. Scale bar = 200 μm. **i** siGFP control animal showing ectopic tentacle formation after AZK treatment. Scale bar = 200 μm. **j** Double axis phenotype with ectopic tentacles near the head region in hydras treated with AZK after HAS-7 (2 + 3)/GFP siRNA electroporation. The asterisk denotes the peduncle region. Scale bar = 500 μm. **k** Both heads in HyWnt3P::HyWnt3 transgenic animal treated as in j exhibit hypostomal *HyWnt3* expression (arrow). Smaller spots along the body column represent ectopic organizers that usually give rise to ectopic tentacles as in **i**. Scale bar = 200 μm. **l** Ectopic axis phenotype in actin::HyWnt3 transgenic hydra. Red arrows indicate multiple secondary heads. **m** No double axis was observed in hydras after HyDkk1/2/4/GFP siRNA electroporation. Scale bar = 500 μm. **n** Double axis phenotype in hydras after HyDkk1/2/4/GFP siRNA electroporation and AZK treatment. Scale bar = 500 μm. **o** Rescue of double axis phenotype in animals treated with AZK after electroporation with a combination of HAS-7 (2 + 3) and HyWnt3 siRNAs. Scale bar = 200 μm. **p** No double axes were observed in hydras treated with AZK after electroporation with HAS-1/GFP siRNAs. **q** Ratios of double axis phenotypes in hydras after electroporation with siGFP or combinations of siRNAs as indicated. In animals without subsequent AZK treatment double axes were counted 6 days after electroporation. In animals treated additionally with AZK, incubation was started 6 days after electroporation and the numbers of double axes in each group were counted 5 days after AZK removal. Total numbers of animals with double axis phenotype in each group were: siGFP/DMSO = 0/192 (*n*=5), siGFP/AZK = 10/230 (*n*=5), siHAS-7/siGFP = 90/186 (*n*=4), siHAS-7/siGFP/AZK = 203/248 (*n*=6), siHAS-7/siHyWnt3/AZK = 11/203 (*n*=3), siHyDkk1/2/4/siGFP/AZK = 65/290 (*n*=3), siHMP1/siGFP/AZK = 93/204 (*n*=3), siHAS-1/siGFP/AZK = 1/150 (*n*=3). Results from at least three independent experiments are shown. Each column represents the total percentage of one group, bars indicate the mean ± S.E.M. ****P value < 0.0001, ***P value < 0.0005, **P value < 0.001. ns = not significant. The data were analyzed using an unpaired parametric *T* test with Welch’s correction followed by pairwise multiple comparisons of each group with the other groups. The individual data values are shown in Additional file 14

We next asked whether *HAS-7* depletion perturbs normal axis formation as a consequence of diminished HyWnt3 processing. siRNA gene expression knockdown by electroporation of adult polyps has recently been established as a robust method in *Hydra* [12, 26, 27]. We designed siRNAs targeting conserved motifs in the HAS-7 pro-domain (siRNA1), catalytic domain (siRNA2) and a less conserved region in the C-terminal ShKT domain (siRNA3). To be able to monitor the electroporation efficiency in the endoderm we used an endodermal actin::GFP/ecdodermal actin::RFP transgenic strain (reverse watermelon) and applied siGFP in combination with target gene siRNAs [12, 26]. Although the knockdown phenotype for the strongly expressed GFP transgene is largely half-sided, we have evidence from previous studies that for native target genes the effect can be systemic [27]. To monitor the gene knockdown at protein level, we raised a polyclonal antibody against a unique epitope in the HAS-7 primary sequence located between the catalytic domain and the N-terminal ShKT domain (APPTAGPTISPT). A Western blot analysis of the different body part lysates used for the initial proteolytic assay showed a broad band migrating at ~ 40 kDa, which was strongest in the head tissue lysate (Fig. 3a, Additional file 6: Fig. S4a). Upper and lower body tissues showed diminished signals reflecting the decreasing HyWnt3-His proteolytic activity observed in the respective body parts (Fig. 1b). The calculated molecular mass of full-length HAS-7 is 37.9 kDa, the mature proteinase lacking signal and pro-peptide sequences is predicted to have 33.3 kDa. Upper and lower body lysates showed additional bands at 55 and 70 kDa, which might be the result of a cross-reaction with a related epitope sequence, or they represent a dimeric form of mature HAS-7 as described previously for meprins [28]. A recombinant histidine-tagged version of HAS-7 protein expressed in High Five insect cells could be detected by the specific Has-7 antibody (Fig. 3b, Additional file 6: Fig. S4b). The apparent molecular mass of recombinant HAS-7 was slightly higher compared to the main band in the HL reflecting the additional amino acids of the tag sequence (1.7 kDa). The heterogenous band pattern of HAS-7 detected in the HL (See Additional file 6: Fig. S4c for a higher resolution of the band pattern) likely represents a mixture of posttranslationally modified and immature forms of the proteinase [20, 29, 30]. Interestingly, when we repeated the cation exchange chromatography using full hydra tissue lysate, HAS-7 was detected as a band at ~ 40 kDa in the input, but a more prominent band migrating at ~ 30 kDa appeared additionally in the peak fractions, indicating pro-domain processing (Additional file 6: Fig. S4d, upper panel). Cleavage of HyWnt3-His was most effective using the elution fraction that showed the highest HAS-7 band intensity, suggesting that HAS-7 is directly responsible for HyWnt3-His processing (Additional file 6: Fig. S4d, lower panel).

siRNA knockdown with a combination of siRNAs 1/2 led to a moderate reduction of the 40 kDa band intensity as detected in whole body lysates (Fig. 3c). The combination of siRNAs 2/3 completely reduced band signals in this region and at the 70 kDa position. The 55 kDa band showed equal intensity in all conditions suggesting that it likely represents an unspecific protein. When we monitored HyWnt3-His stability in HL of *HAS-7* siRNA(2/3) versus siGFP treated animals, HyWnt3-His processing was significantly impaired after *HAS-7* knockdown (Fig. 3d, e), confirming that the HAS-7 proteinase is responsible for the observed HyWnt3-His cleavage. The siRNA knockdown was additionally verified at the transcriptional level by quantitative RT-PCR analysis using head tissues from siGFP and siHAS-7 treated animals (Fig. 3f).

In steady-state hydras, *HAS-7* mRNA depletion led to the induction of a single ectopic axis with a fully developed and functional head structure (Additional file 7: Fig. S5a-b) in about 50% of the animals (Fig. 3g, h, q). The secondary axis phenotype in these animals, as further illustrated in Additional file 8: Fig. S6, was either symmetric starting with a secondary head near the original head structure (Additional file 8: Fig. S6a) or evolved at the budding region in the lower part of the gastric region (Additional file 8: Fig. S6b-c). In rare cases, the secondary head also induced the formation of an ectopic foot as shown in Additional file 7: Fig. S5a. We conclude from this that the increase of HyWnt3 activity induced by *HAS-7* silencing leads to a secondary axis as a consequence of a larger area of organizer-competent tissue. To confirm that this process is due to an increased Wnt signaling level, we challenged electroporated hydras by treatment with AZK, a glycogen synthase kinase-3β inhibitor that leads to a systemic increase of beta-Catenin activity. This treatment normally induces ectopic tentacles along the gastric region 2-3 days after AZK removal emanating from small spot-like organizers that transiently express *HyWnt3* (Fig. 3i, Additional file 7: Fig. S5c-e) [4]. siHAS-7 electroporated animals that additionally received an AZK pulse showed fewer ectopic tentacles, but formed double axes at a constant rate of 80-90% as compared to siGFP electroporated polyps, indicating that this phenotype dominates under conditions of increased basal levels of beta-Catenin activity (Fig. 3i, j, q, Additional file 8: Fig. S6d-g). Ectopic tentacles in these animals were mostly formed near the existing main tentacles, probably due to inhibitory signals from the secondary organizer subduing further organizing centers in the body column [31, 32]. A transgenic reporter line expressing *eGFP* under control of the *HyWnt3* promotor (HyWnt3P::eGFP) [9] confirmed that the ectopic head induced by *HAS-7* siRNA knockdown harbors a functional head organizer with hypostomal *HyWnt3* expression (Fig. 3k). Of note, small ectopic *HyWnt3* expression spots were detected along the body of the HyWnt3P::GFP reporter animal treated with siHAS-7/AZK, although ectopic tentacles were lacking. Interestingly, a double and often multiple axis phenotype was also observed in a transgenic strain that expresses *HyWnt3* under control of the actin promoter in all endodermal epithelial cells (Fig. 3l, Additional file 8: Fig. S6h-i). Similar to the siHAS-7/AZK treated animals ectopic tentacles were mostly formed near the secondary head region. This observation is in line with findings by Watanabe et al. [33] in which an expanded *HyWnt3* expression domain in the budding zone induced a higher number of head-specific tentacles in newly formed buds. Double axes were also obtained when animals were electroporated with siRNAs targeting the Wnt antagonist *HyDkk1/2/4* (Fig. 3m, n), although animals in this group regularly showed in addition multiple ectopic tentacles along the body column. *HyDkk1/2/4* expression has been shown to be down-regulated in areas of elevated beta-Catenin signaling as in the body region of animals treated with ALP [11]. The observation that in siHyDkk1/2/4/AZK treated animals a mixed phenotype (ectopic tentacles and secondary axis) is obtained might be explained by the fact that *HyDkk1/2/4* silencing increases the responsiveness of target cells to HyWnt3, but does not have an effect on overall HyWnt3 levels, which are supposed to inhibit the tentacle system (see below).

Taken together, depletion by siRNA knockdown efficiently reduced HAS-7 protein levels as well as HyWnt3-His proteolytic activity of the respective HL and resulted in a double axis phenotype that was more pronounced in AZK-treated animals. This is likely a consequence of unphysiologically increased HyWnt3 activity as similar phenotypes were observed by transgenic *HyWnt3* overexpression or *HyDkk1/2/4* depletion. Our notion is further supported by the finding of Vogg et al. that siRNA silencing of the *HyWnt3* transcriptional repressor sp5 was shown to induce a multi-headed phenotype at the budding region [10]. We tested our assumption by combining *HyWnt3* and *HAS-7* siRNAs under AZK conditions, which resulted in a complete reversal of the double axis phenotype (Fig. 3o, q). This is in line with previous findings that high beta-Catenin signaling is responsible for the development of ectopic head structures [10]. Interestingly, *HMP1* siRNA knockdown combined with AZK treatment also resulted in ectopic axis induction, but at a substantially lower level than for *HAS-7* (Fig. 3q), indicating some redundancy among astacins with similar expression patterns. No secondary axes were observed in animals electroporated with *HyDkk* and *HMP1* siRNAs without subsequent AZK treatment. siRNA knockdown of *HAS-1*, which does not exhibit a graded apical-distal expression pattern, did not result in double axis formation after AZK treatment (Fig. 3p, q).

In contrast to sp5 (RNAi) animals described in Vogg et al. [10], hydras bisected after *HAS-7* siRNA electroporation did not regenerate ectopic heads or axes (Additional file 9: Fig. S7a-d). This indicates that HAS-7 is not directly involved in the de novo formation of the organizer, but rather in maintaining the dominance of a single organizer after it has been established (see below). This is further supported by the gene expression profiles of the HyWnt3(+) astacin genes generated from the *Hydra* head regeneration transcriptome [34]. As shown in Additional file 9: Fig. S7e, the majority of the astacin genes are downregulated in the regenerating head tissue and thus excluded from the area of the newly forming head structure, which is dominated by *HyWnt3* expression. HAS-4, -5, -6 are an exception as they follow the kinetics of *HyWnt3/beta-Catenin* expression in the regenerating tip, which might indicate a role in tissue or ECM remodeling [35].

Ectopic axis formation resembles *Hydra*’*s* lateral budding process, with the exception that no separation from the original animal occurs as during asexual reproduction. Previously, Watanabe et al. have shown that the induction of a new head organizer in an asymmetrical pattern is dependent on *nodal-related* (*Ndr*) that is transiently expressed in *Hydra’s* lateral bud anlage [33]. When we silenced *Ndr* together with *HAS-7* by electroporation, AZK treated animals were completely devoid of ectopic axes, but showed a normal ectopic tentacle phenotype (Additional file 10: Fig. S8a, f). This demonstrates that *nodal*/*pitx* signaling is recruited for secondary axis formation but is not involved in the induction of ectopic tentacles by AZK treatment [33]. Our finding provides molecular evidence for the theoretical assumption by Meinhardt that head and bud formation proceed by the same patterning process and are independent of the tentacle system [31, 32]. Silencing of *HAS-7* together with *HyWnt8*, which has been shown to be expressed in the tentacle buds [36], resulted in reduction of ectopic tentacle development after AZK treatment and a retarded secondary axis formation (Additional file 10: Fig. S8b, d, f). Interestingly, in contrast to siHAS-7/AZK treated animals, hydras in this group showed in part multiple secondary axes (~ 10%) (Additional file 10: Fig. S8c, f).

In summary, we propose that *Has-7* depletion increases the source density in the body tissue thereby allowing secondary axis development. The newly formed axes release signals that inhibit the tentacle system distant from the head region [31, 32]. The formation of ectopic tentacles by a global increase of beta-Catenin, on the other hand, seems to be at the expense of the axis-forming potential. This suggests a competition between tentacle and head formation at regions of high positional value as predicted by Meinhardt’s model of pattern formation [31, 32]. The retarded appearance of secondary heads in the siHAS-7/siWnt8/AZK treated group might be a consequence of this competition under conditions of tentacle inhibition, as tentacle formation is supposed to precede hypostome development. As previously shown by Lengfeld [37] this process can be simulated by a prolonged continuous treatment of hydras with a low concentration of ALP (0.2 μM). This treatment does not induce the ectopic tentacle phenotype resulting from a short pulse of high ALP, but instead leads to the formation of multiple secondary axes after ~ 3 weeks (Additional file 10: Fig. S8e).

**Fig. 4:**
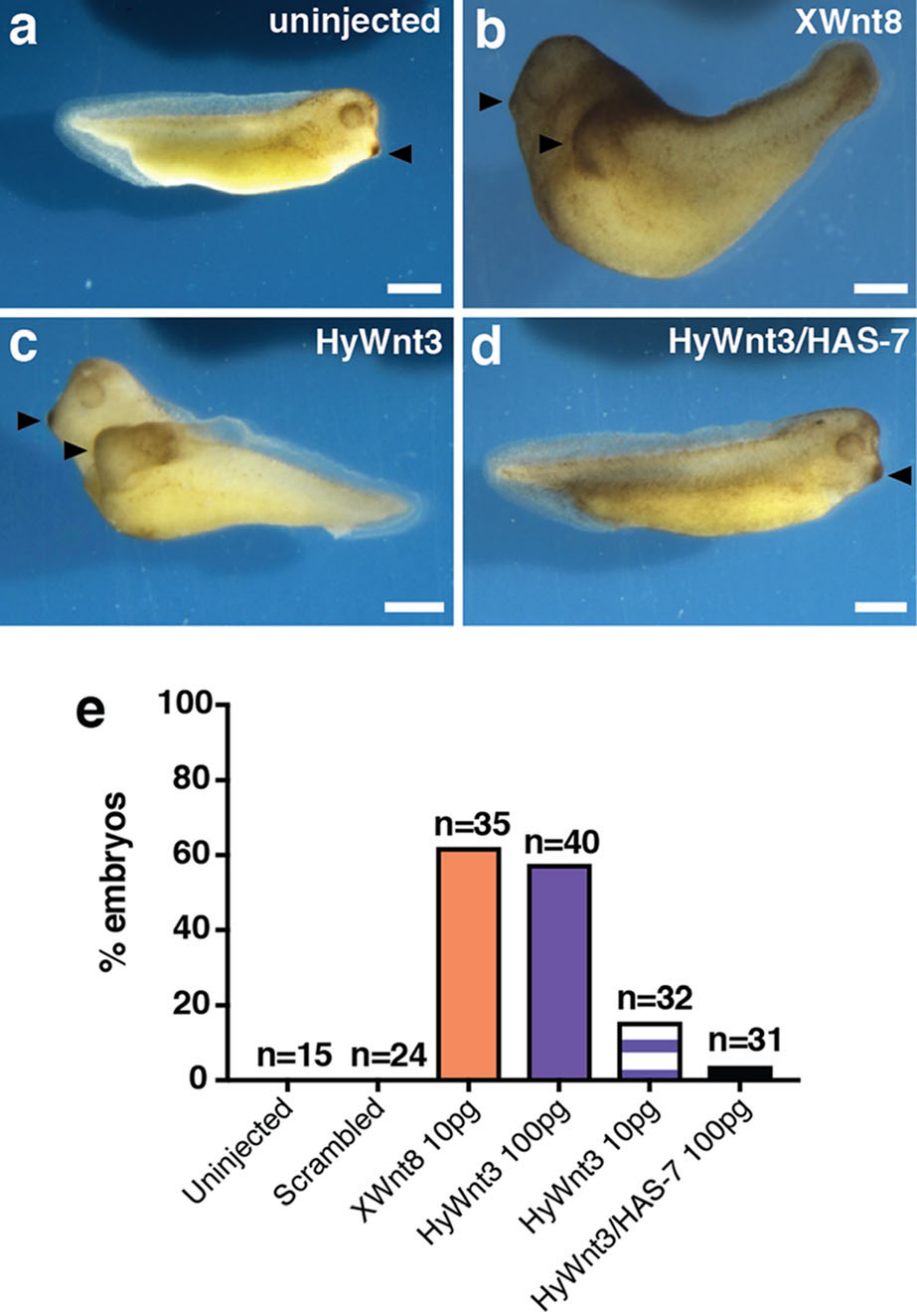
HAS-7 inhibits HyWnt3 induced ectopic axis in *Xenopus laevis* axial duplication assay. **a**, **b** *Xenopus* Wnt8 (XWnt8) mRNA injection induces axis duplication in *Xenopus* embryos compared to uninjected controls. **c** HyWnt3 mRNA induces axis duplication comparable to XWnt8 mRNA. **d** Double injection of HyWnt and HAS-7 mRNAs reverts the axis duplication phenotype. **e** Quantification of embryos showing a double axis phenotype. Injected mRNA doses were as indicated. n indicates the number of embryos analyzed for each experimental condition. Arrows indicate cement glands. Scale bars = 100 μm.

To obtain further evidence that HAS-7 is responsible for HyWnt3 inactivation *in vivo* we made use of the standard axial duplication assay in *Xenopus laevis* (Fig. 4). Injection of 10 pg *XWnt8* mRNA in each blastomere of four-cell embryos induced secondary axis formation in more than 60% of the embryos compared to controls (Fig. 4a, b, e). Although *HyWnt3* injection induced secondary axes as well (Fig. 4c), higher mRNA doses (100 pg) were necessary to reach a comparable level to *XWnt8* (Fig. 4e). Co-injection of 100 pg *HAS-7* mRNA inhibited *HyWnt3*-induced secondary axis formation (Fig. 4d, e), indicating that *HAS-7* is sufficient to antagonize *HyWnt3* function in a heterologous setting.

### beta-Catenin dependency of *HAS-7* expression

**Fig. 5:**
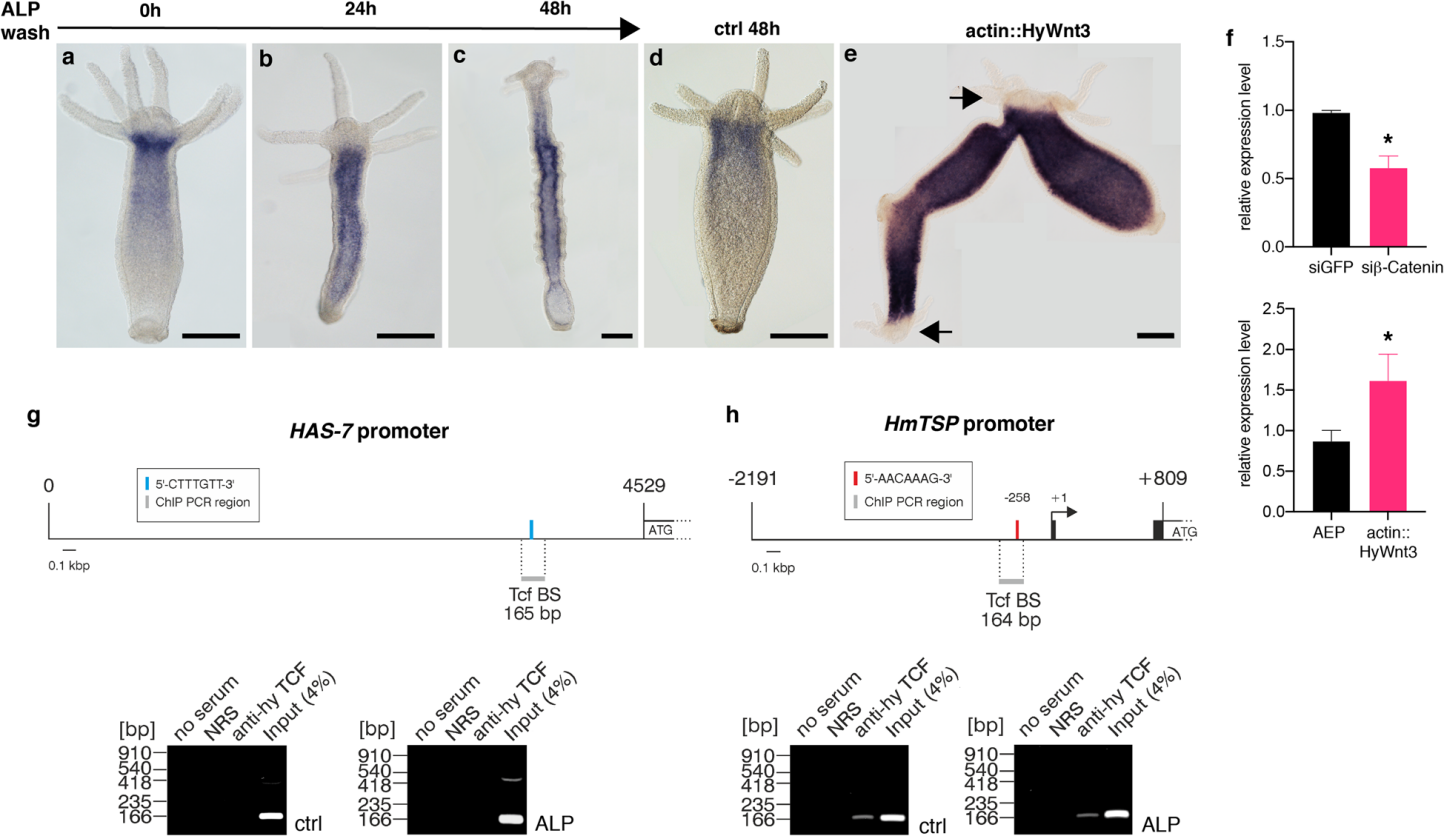
beta-Catenin dependent expression of *HAS-7*. **a**–**c** ISH analysis of *HAS-7* expression after ALP treatment shows a global upregulation after 24 h and a shift towards the developing ectopic organizers along the body column after 48 h as compared to DMSO-treated controls (**d**). At 0 h after ALP wash (**a**) no change of the *HAS-7* expression pattern compared to untreated controls (compare **d** and Fig. 2c) was evident. Scale bars = 150 μm. **e** *HAS-7* expression is globally upregulated in the gastric region of a transgenic actin::HyWnt3 animal with ectopic axis. Scale bar = 250 μm. Arrows indicate head structures. Representatives of 10 hydras examined. **f** Quantitative real-time PCR analysis of *HAS-7* expression confirms the upregulated expression in actin::HyWnt3 animals compared to steady-state AEP animals (lower panel). Inhibition of *beta-Catenin* by siRNA knockdown reduces *HAS-7* expression levels compared to siGFP treated controls (lower panel). Relative expression level is given in 2^(-ΔΔCt)^. Results represent mean +/− S.D. from 3 independent experiments, analyzed by t tests. *p < 0.05. The individual data values are shown in Additional file 14. **g**, **h** No detectable binding of *Hydra* TCF to the *HAS-7* promoter. **g** ChIP analysis of the *Hydra magnipapillata HAS-7* promoter. Upper site: Topography of the *HAS-7* 5′-untranslated region (nt 1 to 4529). The ATG indicates the translation start site. The position of a canonical TCF binding motif (5′-CTTTGTT-3′) is indicated by a blue bar. The localization of the 165-bp DNA segment flanked by the specific ChIP primer pair is visualized with a grey bar. Lower site: ChIP analysis of the *Hydra HAS-7* promoter region using chromatin from untreated whole hydra animals (ctrl), and from animals treated with ALP. A polyclonal antibody directed against *Hydra* TCF was used for precipitation, followed by PCR amplification of the indicated fragment from the *HAS-7* regulatory region. Reactions with normal rabbit serum (NRS) or total chromatin (Input) were used as controls (*n* = 2). PCR products were resolved by agarose gel electrophoresis, and visualized by ethidium bromide staining. **h** ChIP analysis of the *HmTSP* promoter performed under the same conditions as in **g** and used as a positive control. Upper site: Topography of the 3,000-bp *HmTSP* promoter (nt − 2191 to + 809). Black boxes depict the first two exons of the *HmTSP* gene. The arrow indicates the transcription start site of the *HmTSP* mRNA, and ATG the translation start site. The position of the tested canonical TCF binding motif (5′-AACAAAG-3′) is indicated by a red bar. The localization and size of a 164-bp DNA segment flanked by the specific ChIP primer pair is visualized with a grey bar. Lower site: ChIP analysis as described under (**g**) (*n* = 2).

The *HAS-7* expression zone, similar to the one of *HyDkk1/2/4* [11], is distinctly excluded from the region of Wnt expression in the hypostome area. This questions a direct beta-Catenin/TCF-dependent regulation of *HAS-7* as previously demonstrated for *HmTSP* that is expressed by *HyWnt3*-positive cells of the organizer [12]. When *HAS-7* gene expression was monitored by WISH following Alsterpaullone (ALP) treatment, we observed a global increase within the gastric region after 24 h, resulting in a loss of the original apical-distal expression gradient (Fig. 5a, b, d). 48 h after ALP wash, a line of increased *HAS-7* expression was evident close to regions of ectopic tentacle formation (Fig. 5c). In transgenic actin::HyWnt3 *Hydra*, *HAS-7* was strongly upregulated throughout the gastric region (Fig. 5e, f), confirming that the observed increase of *HAS-7* expression induced by ALP is likely a consequence of global beta-Catenin activation. Interestingly, the head region was free of *HAS-7* mRNA in actin::HyWnt3 transgenic animals. When we silenced *beta-Catenin* by siRNA electroporation, *HAS-7* expression was down-regulated as evidenced by quantitative RT-PCR analysis (Fig. 5f). Upon examination of the *HAS-7* promoter region for regulatory elements, we identified a single putative TCF binding element with the conserved sequence motif 5′CTTTGTT3′ (Fig. 5g), similar to those experimentally confirmed in the *HyWnt3* [9] and *HmTSP* [12] promoters (CTTTGWW, W=A or T). To gain evidence whether *HAS-7* is directly regulated by beta-Catenin/TCF, we performed a chromatin immunoprecipitation (ChIP) assay using a *Hydra* TCF-specific antiserum as described previously [12, 38]. Chromatin was taken from control polyps and ALP-treated animals. No PCR product was obtained with primers flanking the identified TCF binding element in the *HAS-7* promoter in control and ALP conditions (Fig. 5g). In contrast, amplification of the TCF binding element in the *HmTSP* promoter yielded the expected PCR product from both samples (Fig. 5h). This suggests that TCF is not bound to the putative DNA binding region in the *HAS-7* promoter both in steady-state polyps and under conditions of global increase in nuclear beta-Catenin. We therefore presume that *HAS-7* is an indirect downstream target of beta-Catenin/TCF, which is regulated as part of the global Wnt signaling response, but is controlled by additional factors that down-regulate its expression in the vicinity of Wnt producing cells.

### Substrate specificity of the HAS-7 proteinase

**Fig. 6:**
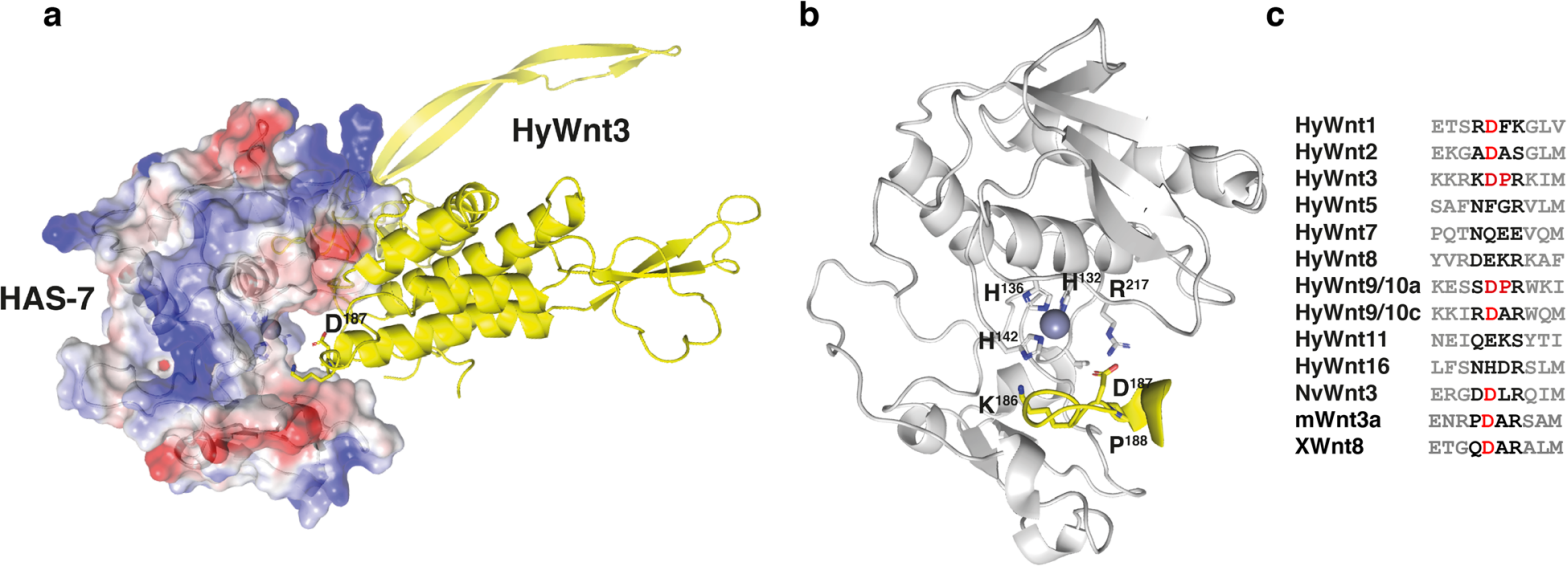
Structural homology model of the putative HyWnt3:HAS-7 complex. **a** Overview of HAS-7 (surface potential) complexed with HyWnt3 (yellow cartoon presentation). The active site Zn-ion of the astacin is shown as pink sphere. **b** Detailed view inside the active site pocket, shown in standard orientation with the Zn-ion located at the bottom of the cleft. **c** Alignment of amino acid sequences in different *Hydra* Wnt proteins encompassing the putative DP cleavage motif in HyWnt3. NvWnt3, *Nematostella vectensis* Wnt3; mWnt3, mouse Wnt3; XWnt8, Xenopus Wnt8

Altogether seven Wnt genes are expressed in the adult hypostome of *Hydra* (HyWnt1, -3, -7, -9/10a, -9/10c, -11, and -16) [7]. The size of their respective expression domains differs, with Wnt1, Wnt3, Wnt9/10a, c and Wnt11 showing a more confined spot-like expression reaching from the tip to the apical half of the hypostome, while Wnt7 and Wnt16 exhibit broader expression domains that extend to the tentacle bases. Wnt9/10b is not expressed in the adult polyp and Wnt5/8 are tentacle-specific [36]. Wnt2 is distinct in exhibiting a transitory expression at the site of bud initiation [7]. During head regeneration, the hypostomal Wnts are expressed in a temporally graded fashion with Wnt3, Wnt9/10c and Wnt11 being at the top of the cascade. Recombinant HyWnt3-His has been shown to induce head-forming capacity in tissue of the gastric region [7], indicating a primary role in initiating head formation. Our data indicate a redundant expression of several astacin proteinases in the sub-tentacle region with possibly similar substrate specificity. We have no evidence whether all head-specific Wnts are degraded by HAS-7 and closely related astacins, but considering that HyWnt3 acts at the top of the signaling cascade, it might serve as the primary substrate to inactivate the generation of a secondary organizer. When we screened the HyWnt3 amino acid sequence for putative cleavage sites for astacins, which were recently shown to have a strong preference for aspartate in the P1’ position (first amino acid C-terminal of the cleavage site) and proline in P2’ or P3’ in substrate peptides [39], only D187 followed by Pro188 fulfilled this criterion in HyWnt3. To evaluate if this is supported by the structural properties of the two proteins we created a homology model of the HyWnt3:HAS-7 complex using X-ray crystal structures of Wnt8 (pdb 4f0a) [40] and pro-meprin ß (pdb 4gwm) [41] as templates (Fig. 6a, b). This docking model was refined by using the coordinates of mature (active) crayfish astacin [42, 43], of mature crayfish astacin complexed with a transition state analogue phosphinate inhibitor (pdb 1qji) [44] and of zebrafish hatching enzyme ZHE-1 (pdb 3lqb) [45], which is the closest relative of HAS-7 with a known 3D structure. The N-terminal pro-domain, which confers protection of the active site, is supposed to be cleaved in order to transition the inactive zymogen into a catalytically potent astacin [20, 29, 30]. As a consequence, the alpha-helical N-terminal domain of HyWnt3 would be able to approach the active site cleft of HAS-7 opposite to the lipid-mediated Frizzled receptor binding site [40] (Fig. 6a). The KDP motif of the putative cleavage site in HyWnt3 is predicted to be positioned where the pro-domain cleavage site would reside. In the model, it is embedded in the deep active-site cleft, which spans the entire catalytic domain like a horizontal trench (Fig. 6b). The catalytic zinc ion is supposed to reside at the bottom of the active site cleft and is complexed with the imidazole moieties of His132, His136, His142, and with the phenolic hydroxyl of Tyr191 including a solvent molecule with respect to the trigonal bipyramidal coordination sphere around the zinc ion in mature astacins. HyWnt3 is predicted to bind to HAS-7 in an extended conformation and to be anchored to the proteinase cleft in an antiparallel manner. Astacins are the only extracellular proteinases with a primary specificity for negatively charged residues (preferably aspartate) in the position C-terminally of the cleaved peptide bond [39] in the protein target and the elongated KKRK*DPRKIM motif would allow for most efficient cleavage. The negatively charged aspartate side chain at the P1’ position is presumably bound by the positively charged arginine (R217) in the S1’ substrate binding site of HAS-7. In addition, a proline in close proximity in P2’ as given in the HyWnt3 sequence is frequently observed in astacin substrates [39, 46]. This type of positive subsite cooperativity induced by proline in P2' has been observed in meprin α [39] and crafish astacin [46]. By contrast, in meprin ß the opposite effect has been observed; there, proline in P2' decreases the abundance of Asp or Glu in P1' [46]. HAS-7 seems to be more similar in specificity to meprin α than to meprin ß. This is supported also by the fact that the latter contains an additional, second arginine residue the S'-region which increases the abundance of cleavages at di-acidic sites [39].

Mutation of the D187-P188 motif in HyWnt3 to alanine led to highly decreased secretion levels of the respective protein, indicative of protein instability or impaired folding in the secretory pathway, which precluded a meaningful cleavage assay. Upon inspection of all *Hydra* Wnt sequences we found the DP motif at this position to be fully conserved only in HyWnt9/10a (Fig. 6c). Interestingly, non-canonical Wnts HyWnt5 and HyWnt8 [36] that are confined to the tentacle region and Wnts that have a broader expression domain in the head, like HyWnt7 and HyWnt16, were less conserved in this region and even lacked the aspartate residue. It is an attractive hypothesis that these Wnts act in confining the expression of HAS-7 and of related astacins to the sub-tentacle region.

### HAS-7 function ensures single organizer dominance in an extended activator-inhibitor model

To investigate the role of HAS-7 during axis and head pattern formation in *Hydra*, we built a mathematical model describing HAS-7-HyWnt3 spatio-temporal dynamics as an extension of the classical activator-inhibitor models of Gierer and Meinhardt [31, 32, 47]. We used the framework of a hypothetical two-component activator-inhibitor concept based on current experimental evidence of HyWnt3 and beta-Catenin/TCF dynamics and employ our experimental findings concerning HAS-7. In particular, we extend the previous models by explicitly distinguishing between beta-Catenin/TCF and HyWnt3 driven pattern formation. In addition, we verify how HAS-7 modulates formation of the body axes or the head in perturbed conditions (such as beta-Catenin/TCF upregulation).

**Fig. 7:**
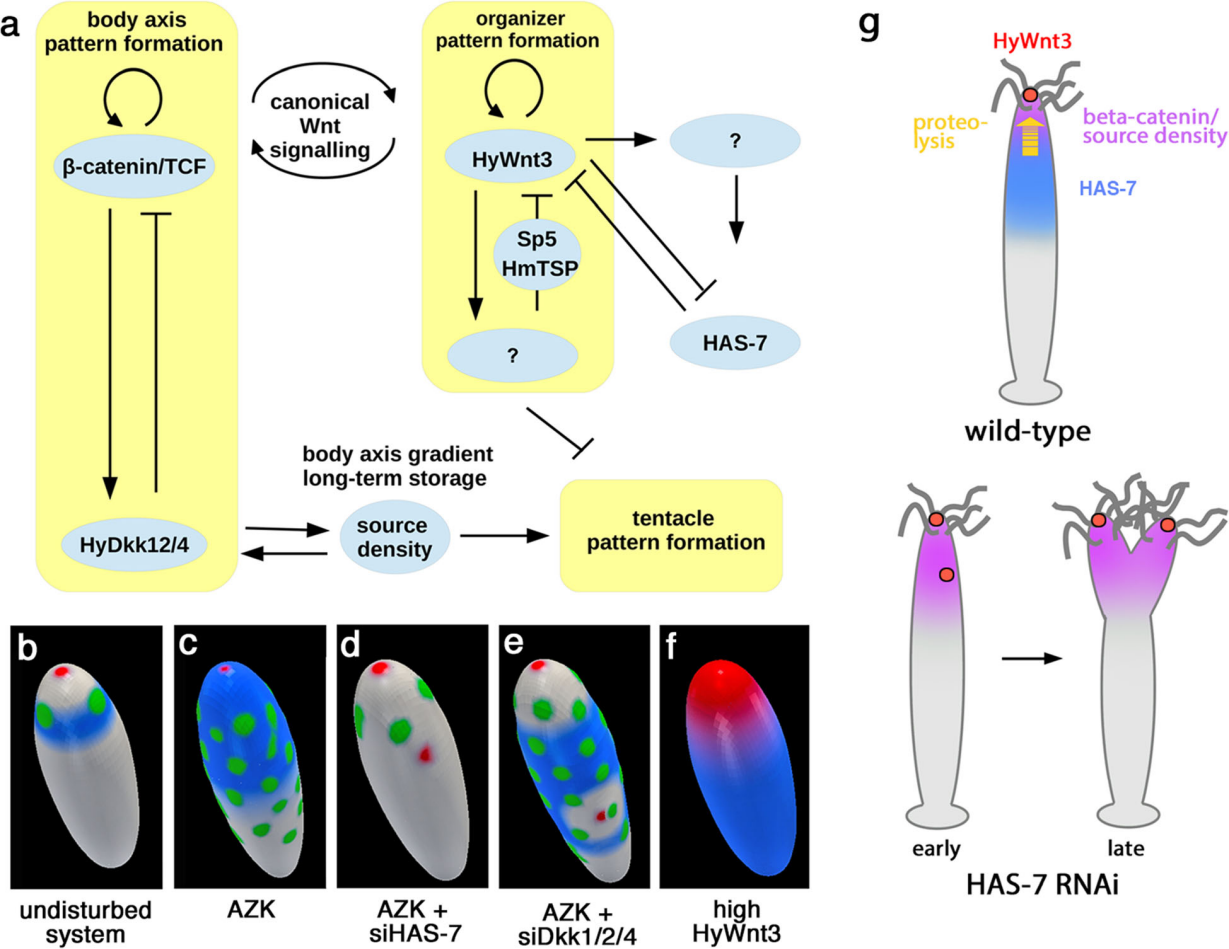
Mathematical model of HAS-7 function. **a** Schematic representation of the model. Yellow blocks depict self-containing pattern formation systems consisting of an activator and an inhibitor. **b**–**f** Numerical simulations of different experimental scenarios. The blue color corresponds to high levels of *HAS-7* expression, the red color to *HyWnt3* expression, and the green color indicates the formation of tentacles. The scale of these expression intensities is similar across all simulation plots. **g** Schematic diagram summarizing the role of HAS-7 in restricting the organizer in steady-state hydras and double axis induction after experimental *HAS-7* gene silencing

A scheme of the HAS-7-Wnt3 model is depicted in Fig. 7a. Based on our findings we postulate a negative feedback loop between HyWnt3 and HAS-7 (see methods for more details), representing one of the most frequent network motifs directing body-axis fine-scale segmentation in animals [48]. Additionally, we assume that *HAS-7* expression is placed downstream of the organizer/HyWnt3, and thus it is only indirectly positively affected by beta-Catenin/TCF. In particular, the HyWnt3-HAS-7 interaction model comprises two subsystems describing the organizer and the axis formation, which act on different spatial scales. These include a small-scale HyWnt3-driven organizer pattern formation vs. a beta-Catenin/TCF signaling controlled large-scale body axis formation, as well as an additional subsystem directing tentacle formation (yellow blocks in Fig. 7a). The latter does not influence HAS-7-Wnt3 interactions. However, because it is affected by the body-axis and head system [31, 32], its integration into the model allows additional model verification using, among others, data obtained under AZK treatment.

As depicted in Fig. 7b, simulating the unperturbed system leads to the formation of a regular ring of tentacles below the hypostome. Furthermore, a single *HyWnt3* expression spot appears at the tip of the hypostome and *HAS-7* expression shows a distinct ring-like pattern. Similar patterns are observed in *HAS-7* siRNA knockdown simulations without AZK treatment as modelling parameters were adjusted to preclude secondary axis formation in the unstimulated state. Simulated AZK treatment assumes a uniform increase of the head forming potential [32]. As the latter is, among others, a multiplier of the production term of the tentacle activator, its increase along the body column allows reaching the threshold necessary for tentacle formation. Existence of such a threshold follows experimental observations while, in the model, it is regulated by the baseline inhibitor production. Consequently, the model predicts multiple ectopic tentacles and elevated *HAS-7* expression in the entire body column without a formation of additional axes (Fig. 7c). In contrast, simulations of the AZK treatment with *HAS-7* depletion (Fig. 7d) results in loss of ectopic tentacles and development of a secondary axis. Furthermore, the model of AZK treatment combined with *HyDkk1/2/4* depletion leads to formation of a secondary organizer including the formation of ectopic tentacles (Fig. 7e) as it does not have an effect on overall HyWnt3 levels, which are supposed to inhibit the tentacle system. Finally, *HyWnt3* overexpression (Fig. 7f) leads to a strong and body-wide expression of *HAS-7*. The only disagreement between simulations and experimental observations is that a simulated *HyWnt3* overexpression does not lead to a multiple axis phenotype but to a broadened *Wnt3* expression domain at the head (Fig. 7f). The reason for this is that we describe each pattern formation system by an activator-inhibitor model, and an inherent mathematical property of this class of models does not allow changes in the activator production to cause an increase in the number of resulting heads. Thus, the applied activator-inhibitor model may be seen as an approximation of a de novo pattern formation mechanism that is still unknown. In this context it is worth mentioning that de novo patterning may be based on not only chemical processes, but may also result from mechanochemical, cellular or bio-electrical processes as well [49–51].

In summary, the proposed model qualitatively reproduces several of our experimental observations, including those which we did not even consider in the modelling process, such as the absence of ectopic tentacles in *HAS-7*-depleted animals treated with AZK. Analyzing the impact of the model assumptions on the simulation results in the context of experimental data, we conclude that the function of HAS-7 is, most likely, to prevent the formation of multiple *HyWnt3* spots. In particular, the dominance of a single organizer is ensured by HAS-7-driven degradation of the canonical Wnt ligands (Fig. 7g). We assume that this task is especially important when beta-Catenin/TCF levels are increased, which may occur in *Hydra* under natural conditions, for example in interaction with the environment [52], or as a response to injury [38].

## Conclusions

Metalloproteinases have a broad spectrum of activities and can be involved in extracellular matrix remodeling but also in processing of signaling molecules in a variety of developmental events. Here, we show that a member of the cnidarian astacin proteinase family acts as a negative feedback regulator of Wnt3 activity by direct morphogen processing, thereby providing a limitation of high canonical Wnt activity to the hypostomal organizer. Using a dense interplay between new experimental data and mathematical models/simulations we present a revised interaction scheme of main molecular players involved in *Hydra* head pattern formation. In particular, our results suggest that metalloproteinase activity prevents the formation of multiple organizers in regions with high head forming competence (Fig. 7g). Given the complex expression pattern of Wnt ligands in *Hydra*’*s* head organizer, it will be interesting to unravel possible fine-tuned substrate specificities of the seemingly redundant astacin proteinase presence insulating the head region. We speculate that individual Wnts might have diverse functions in the patterning process of head and tentacles as suggested by the staggered expression dynamics during head regeneration [7]. The sequence variations in substrate binding regions as well as diverging expression domains of the astacins detected in this study might function as a complementary molecular pattern that evolved to maintain signaling homoeostasis. So far, Turing-type models are solely based on gene expression patterns, but do not integrate protein diffusion ranges. Future work addressing this shortcoming will help to refine the complex activator-inhibitor network of Wnt-regulated patterning in *Hydra.*

## Methods

### Animals and whole mount immunocytochemistry

*Hydra vulgaris* was used for all experiments. For siRNA experiments, a transgenic *Hydra vulgaris* strain expressing endodermal GFP and ectodermal RFP (reverse water melon) provided by Robert Steele’s laboratory was used as described before [26]. The transgenic reporter line expressing *eGFP* under control of the *Hydra* Wnt3 promotor was described by Nakamura et al. [9]. The transgenic vector used to generate the strain expressing *HyWnt3* under control of the actin promotor was produced by cloning the full-length *HyWnt3* sequence between the actin promotor and actin 3’ flanking sequences of the pBSSA-AR vector using NcoI and XbaI sites [9]. Generation of the transgenic actin::HyWnt3 line was performed by microinjection as described before [9]. All animals were maintained in artificial *Hydra* medium (HM, 1 mM CaCl_2_, 0.1 mM MgCl_2_, 0.1 mM KCl,1 mM NaH_2_CO_3_, pH 6.8) at 18 °C in polystyrene dishes (Carl Roth) and fed two to three times per week with freshly hatched *Artemia salina* nauplii, unless indicated otherwise. Media was renewed 3–4 h after feeding and again the following day. Animals were starved for 24 h prior to experiments, unless indicated otherwise. Whole mount immunocytochemistry using a CPP-1-specific antibody was performed as described previously [53].

### Wnt proteolysis assays using tissue lysates

*Hydra* Wnt3 cDNA lacking the native leader peptide was subcloned into the pCEP-Pu mammalian expression vector, which introduces a BM-40 signal sequence and a C-terminal histidine tag as described previously [12]. Recombinant HyWnt3 was expressed using transiently or stably transfected HEK293T cells. For this, cells were seeded in full DMEM Medium (Gibco) in 6-well plates and transfected at 70% confluency using 1 μg of the respective vector construct together with 5 μl transfection reagent (TransIT®-LT1 Reagent, Mirus Bio) in 200 μl serum-free DMEM medium after 20 min incubation time. The cells were maintained in a humidified 5% CO_2_ atmosphere at 37 °C. After 48 h the medium was harvested, followed by Ni-NTA sepharose (Qiagen) batch purification. For 1 ml conditioned medium, 40 μl Ni-NTA beads were added and incubated for 1 h at 4 °C under constant rotation. After a 2-min centrifugation step at 500 rpm the beads were washed two times with PBS. Elution was performed with 250 mM Imidazol for 15 min at RT. The beads were centrifuged at 500 rpm and the supernatant containing the histidine-tagged Wnt protein was instantly frozen in liquid nitrogen and stored at -80 °C. The eluted *Hydra* Wnt3 was verified by Western blotting using 10% SDS-PAGE gels. The proteins were transferred to PVDF membranes by wet blotting. Membranes were blocked for 1 hr at RT in PBS containing 5% BSA and 0.2% Tween-20 (PBST), incubated with mouse Penta His antibody (Qiagen, # 34660) at 1:1000 in 1% BSA at 4 °C overnight, washed 3 × 5 min with PBST, and then incubated with anti-mouse horseradish peroxidase-conjugated antibody (Jackson ImmunoResearch, #115-035-044) at 1:10,000 in PBST containing 5% BSA for 1 hr at RT. The membrane was washed 2 × 5 min with PBST and 2 × 5 min with PBS and blots were developed using a peroxidase substrate for enhanced chemiluminescence.

To generate *Hydra* body part lysates, 100 hydras were cut into four parts using a scalpel. Tentacles, head without tentacles, upper and lower gastric region were immediately transferred on ice, sonicated for 1 min in 250 μl ice cold PBS (Branson sonifier 250, pulsed mode, duty cycle 10%, output 1.5), centrifugated at 10,000 rpm for 1 min at 4 °C, snap frozen and kept in aliquots at -80 °C. Body part lysate concentrations were adjusted to 4 mg/ml protein concentration using a nanodrop photometer and verified by Western blot using alpha-tubulin antibody (Sigma-Aldrich, clone DM1A, T9026) at 1:1000. For the proteolysis assay, ~ 10 ng of purified HyWnt3-His was incubated with 15 μg of the respective tissue lysates in a total volume of 12 μl in PBS for 0, 1, 2, 4, 6, 8, and 24 h at RT. Peak fractions from the ion exchange chromatography were incubated for 6 h. For inhibitor treatments, 1, 10-Phenanthroline (Sigma-Aldrich), Batimastat (Sigma-Aldrich) or EDTA (AppliChem) were added at 200 μM, respectively. Fetuin-B was diluted in PBS and incubated for the given time periods as indicated in Fig. 1f) The incubation was stopped by adding SDS-PAGE sample buffer (10% glycerol, 50 mM Tris-HCl pH 6.8, 0.02% bromophenol blue, 2% SDS, 1% 2-mercaptoethanol) and heating at 96 °C for 5 min. HyWnt3-His band intensities were documented by immunoblotting as described above. Unspecific proteolysis in HL was monitored by adding 15 μg of the lysate to 1 μg of BSA (Roth) in a total volume of 12 μl in PBS and incubation at RT. The reaction was stopped by adding sample buffer and heat denaturation at 96 °C for 5 min after the same time periods as for HyWnt3-His. Samples were analyzed using 10% SDS-PAGE gels and Coomassie staining.

Full-length recombinant HyDkk1/2/4-His protein was expressed in *E. coli* BL21 (DE3) cells from a pET15b vector (Novagen) and purified under native conditions from the supernatant of cell pellets lysed by several freeze/thaw steps in PBS. The cleared supernatant was filtered and purified using Ni-NTA agarose beads (Qiagen). The eluted protein solution was dialyzed against PBS and purity was checked by SDS-PAGE. For the proteolysis assay, 10 ng of purified HyDkk/1/2/4-His were incubated with 15 μg of the respective tissue lysates in a total volume of 12 μl in PBS for 0, 1, 2, 4, 6, 8, and 24 h at RT. HyDkk1/2/4-His band intensities were documented by immunoblotting as described above.

The sequence for the *Hydra* Cadherin CAD1-2 domain (15-239) was amplified by PCR using a large 5′ cDNA fragment of *Hydra* Cadherin as template. The PCR product was cloned into the pET19B vector (Novagen) using NdeI and BamHI sites and the recombinant protein was expressed in *E. coli* BL21 (DE3) cells. Inclusion bodies were solubilized in PBS, 8 M urea, and bound to Ni-NTA agarose beads. Refolding was performed by changing to PBS buffer prior to elution with PBS, 250 mM imidazole, 0.4 M L-arginine (AppliChem). The eluted protein solution was dialyzed against PBS and its purity checked by SDS-PAGE. Ten nanograms of the purified protein were used as substrate with 5 μl of either the HyWnt3(+) and HyWnt3(-) HL fraction. Detection of the *Hydra* cadherin fragment by immunoblotting was performed as described above.

The polyclonal HAS-7 specific antibody was raised against a peptide corresponding to a sequence between the catalytic and ShKT domains (CKGGGNPPTGPPTAPP). For Western blotting, a PVDF membrane was blocked in 5% skimmed milk powder in PBS, 0.2% Tween-20, for 1 h at RT. The primary antibody was applied at 1:1000 in 1% skimmed milk powder in PBST and membranes were incubated overnight at 4 °C. A peroxidase-conjugated anti-rabbit antibody (Jackson ImmunoResearch, #111-035-003) was applied in blocking solution at 1:10,000 for 1 hr at RT. Quantification of Western blot band intensities was performed using the ImageStudioLite software. Uncropped Western blot and gel images are shown in Additional File 11: Fig. S9.

### Ion exchange chromatography and mass spectrometry analysis

A pool of head lysate was generated by decapitating 400 animals closely beneath the head region and removing tentacles using a scalpel. Head tissue pieces were resuspended in ice cold 20 mM Tris-HCl, pH 7.5, and lysed on ice by passing at least 10 times through a 20-gauge needle. The lysate was centrifuged for 15 min (14,000 rpm) at 4 °C and the supernatant was applied to anion exchange chromatography using a MonoQ HR5/5 column (GE healthcare). The eluent buffer contained 1 M NaCl in 20 mM Tris-HCl, pH 7.5. Peak fractions indicated in Additional file 1: Fig. S1 were screened for HyWnt3-His proteolytic activity as described above and frozen at − 20 °C until further use. Pooled HyWnt3(+) and HyWnt3(-) fractions were subjected to mass spectrometry analysis after in solution tryptic digestion. Peptide separation was achieved using a nano Acquity UPLC system (Waters). Protein mass spectrometry analyses were performed as previously described [34]. In short: The nano UPLC system was coupled online to an LTQ OrbitrapXL mass spectrometer (Thermo Fisher Scientific). Data dependent acquisition with Xcalibur 2.0.6 (Thermo Fisher Scientific) was performed by one FTMS scan with a resolution of 60,000 and a range from 370 to 2000 m/z in parallel with six MS/MS scans of the most intense precursor ions in the ion trap. The mgf files processed from the MS raw files were used for protein database searches with the MASCOT search engine (Matrix Science, version 2.2) against NCBI GenBank Proteins (version of October 28, 2015). Domain composition of GenBank protein accessions was analyzed against CDD (NCBI) and InterProScan 5 (EMBL-EBI).

For fractionation of full hydra lysates (Additional file 6: Fig. S4d), 1000 animals were dissolved by sonification on ice in 1 ml HEPES(-) buffer (50 mM HEPES in ddH_2_O pH 7.4). The hydra lysate was cleared at 14,000 rpm for 15 min at 4 °C. Five hundred microliters of the supernatant was applied to anion exchange chromatography as described above. All collected fractions were snap-frozen in liquid nitrogen and stored at − 70 °C.

### Expression and purification of recombinant HAS-7

Expression of HAS-7 occurred in insect cells using the Bac-to-Bac system (Invitrogen, Thermo Fisher Scientific). A synthetic fragment of the HAS-7 full-length cDNA (Biomatik) was subcloned into the pCEP-Pu mammalian expression vector, which introduces a C-terminal histidine tag and then the fragment containing the C-terminal His_(6)_ -tag was inserted into the donor plasmid pFASTBac1 to generate bacmids in *E. coli* DH10Bac cells for transfection of insect cells according to the manufacturer’s manual. Amplification of recombinant baculoviruses occurred in *Spodoptera frugiperda* 9 cells (Sf9 CCLV-RIE 203, Friedrich–Löffler Institute, Greifswald, Germany) growing as adherent monolayer in Grace’s insect medium supplemented with 100 U/ml penicillin and 100 μg/ml streptomycin. For expression of HAS-7-His_(6)_ *Trichoplusia ni* cells (BTI-TN-5B1-4/High Five-cells CCLV-RIE 350, Friedrich–Löffler Institute, Greifswald, Germany) were cultured as suspension in Express Five SFM containing 100 U/ml penicillin, 100 μg/ml streptomycin and 16.4 mM L-Glutamine. Infected High Five cells were incubated for 72 h at 27 °C in Fernbach flasks (shaking incubator Multitron, INFORS). Following centrifugation, proteins were precipitated at 10 °C by step wise addition of solid ammonium sulfate to the supernatant resulting in a 60% ammonium sulfate saturation. After further stirring over night at 10 °C and centrifugation (9000×*g*, 90 min, 10 °C) the harvested pellet was resuspended in 1/10 of the expression volume in 50 mM Tris-HCl pH 7.6, 300 mM NaCl, 20 mM imidazole and dialyzed against the same buffer. The cleared supernatant gained after centrifugation (8000×*g*, 10 min, 4 °C) was applied onto a Ni-NTA column (Qiagen). Following several washing steps with 50 mM Tris-HCl pH 7.6, 300 mM NaCl containing increasing imidazole concentrations (20 mM, 50 mM and 100 mM), the protein was finally eluted in a buffer containing 250 mM imidazole. The elution fractions were pooled, dialyzed against PBS, and concentrated (Millipore Amicon Ultra, 3 K). SDS-PAGE and transfer to PVDF were performed as described previously [54]. The membrane was blocked in 3% BSA in TBS for 2 h, incubated for 1 h with Penta-His antibody at 1:2000 and further incubated for 1 hr with secondary antibody (goat anti-mouse POX 1:7500 in 7.5% skimmed milk powder in TBS). After each antibody treatment, three washing steps were inserted (2x TBST, 1x TBS). The Clarity Western ECL Substrate (Bio-Rad) was used for detection.

### In situ hybridization

Customized LNA digoxygenin-labeled RNA probes were designed and produced by Qiagen corresponding to the antisense strands of the respective astacin cDNAs. The whole-mount ISH procedure was performed as described previously [55]. For hybridization, the LNA probe was added to a final concentration of 1 μM in fresh hybridization solution (1:1 mixture of deionized formamide and buffer containing 5x SSC (750 mM NaCl, 75 mM sodium citrate), 0.2 mg/ml yeast tRNA, 2% of 50x Denhardt’s solution, 0.1 mg/ml heparin, 0.1% Tween-20 and 0.1% CHAPS), and hybridized for ~ 60 h at 55 °C. Digoxygenin-labeled RNA probes for *HAS-1*, *HAS-7*, and *HMP1* corresponding to the sense and antisense strands were prepared using an RNA labeling in vitro transcription kit (Roche). ISH probe sequences covered the predicted full-length mRNA sequence for each gene. The further procedure was carried out as described previously [12]. Samples were finally mounted in PBS containing 90% glycerol, or in Mowiol 4–88 (Carl Roth), and images were acquired with a Nikon Digital Sight DS-U1 camera mounted on Nikon Eclipse 80i and imaging software NIS Elements (3.10, SP3, Hotfix, Build645). Further image processing was performed with Adobe Photoshop CS6 and Fiji. ISH probe and LNA sequences are summarized in Additional file 12: Table S3. t-SNE plots for the respective genes were designed using online tools provided at https://singlecell.broadinstitute.org/single_cell/study/SCP260/stem-cell-differentiation-trajectories-in-hydra-resolved-at-single-cell-resolution#study-visualize [25].

### Alsterpaullone treatment for ISH analysis

Eighty budless hydras, which were fed the day before, were incubated in 5 μM ALP (Sigma-Aldrich) in DMSO in 100 ml HM. The animals were kept in the dark at 18 °C for 24 h. After incubation, the HM was changed every day. Samples were taken for ISH at 24 h, 48 h, and 72 h after ALP treatment. Hydras incubated for 24 h in DMSO and fixed after 72 h served as control. For continuous ALP treatment (Additional file 10: Fig. S8e), the medium was exchanged daily and animals were fed once per week.

### Electroporation with siRNAs

siRNAs (HPLC grade) (see Additional file 13: Table S4 for sequences) were purchased from Qiagen. Electroporation of siRNA was performed as described recently [12] using 3 μM of siGFP (1 μM siGFP and 2 μM scrambled siGFP) or a combination of siGFP, scrambled siGFP and target siRNAs. Scrambled siGFP was omitted when two target siRNAs were used. Immediately after the pulse, 500 μl restoration medium consisting of 80% HM and 20% hyperosmotic dissociation medium (6 mM CaCl_2_, 1.2 mM MgSO_4_, 3.6 mM KCl, 12.5 mM TES, 6 mM sodium pyruvate, 6 mM sodium citrate, 6 mM glucose and 50 mg/l rifampicin, 100 mg/l streptomycin, 50 mg/l kanamycin, pH 6.9) was added to the cuvette, the animals were transferred to Petri dishes containing restoration medium and allowed to recover for one day. Viable polyps were transferred to new dishes containing HM and maintained under standard culture conditions. For AZK treatment, animals were incubated at 6 days post-electroporation either with 0.1% DMSO or 50 nM AZK in HM for 16 h. Thereafter, they were rinsed in HM several times and cultured under standard conditions. Five days after AZK treatment, the animals were anesthetized in 1 mM Linalool (Sigma-Aldrich) [56] using a Nikon SMZ25 stereomicroscope equipped with Nikon DS-Ri2 high-definition color camera. Note that AZK instead of ALP was used in siRNA experiments allowing a reduction of the total inhibitor concentration applied on the electroporated animals. Fifty nanomolar AZK induces the same grade of ectopic tentacles as 5 μM ALP when applied in the standard assay published previously [36]. Double axes counted in these experiments were defined morphologically to have a duplication of the head structure with fully developed and functional tentacles and to show a clearly detectable split of the body column along two independent axes. Mostly, these animals were Y- or L-shaped sharing the same lower gastric region and foot region. In contrast to buds, secondary axes do not separate after reaching a certain growth limit.

### Chromatin immunoprecipitation

Chromatin immunoprecipitation analysis was carried out as described recently by using sheared extracts from formaldehyde-treated *Hydra* animals, which have been treated without or with 5 μM ALP (48 h) and an antiserum directed against a recombinant *Hydra* TCF protein [12, 38]. PCR of precipitated DNA was done using specific primers flanking the potential TCF binding sites in the 5′-regulatory regions of the *Hydra HAS-7* or the *HmTSP* gene (Fig. 5g, h). PCR primer sequences: TCF binding motif in *HAS-7* (5′-GCTGTTATCTGTCCGCTTTC-3′/5′-CCATATAGAGGCCACACACC-3′), and the proximal TCF binding motif in *HmTSP* (5′-TTGAAGGCATTTAACAACTTGC-3′/5′-TGCCCAAATGTAAAGTTCTGTG-3′).

### Real-time quantitative PCR

The RNA isolation was performed as described previously [34]. Sixty hydra heads per condition were isolated either from steady state AEP polyps or siRNA treated transgenic reverse water melon animals by removing the tentacles and gastric region using a scalpel. The heads were immediately transferred into 200 μl TRIzol and stored at − 20 °C. For siRNA (siGFP or siHAS-7) treated hydras, head samples were isolated 6 days after electroporation. Complete hydras were used for *beta-Catenin* RNAi treated and transgenic actin::HyWnt3 animals including the respective controls. cDNA synthesis was performed using the SensiFAST cDNA Synthesis Kit (Bioline) according to the manufacturer’s instructions. RT-qPCR was carried out with a StepOnePlus™ instrument (Applied Biosystems, Thermo Fisher Scientific) using the SensiFAST SYBR-Hi-ROX Kit (Bioline) according to the manufacturer’s instructions. The transcript level analysis was done by the ΔΔC(T)-Method with Elongation Factor 1-α (EF-1α) as a house keeping gene for normalization. Three biological replicates were performed for each experiment and triplicate measurements were made for each sample in each experiment. The no-template conditions served as negative controls. The data are presented as relative quantity (RQ) by 2^(-ΔΔC(T)) calculation. qPCR primer sequences are given in Additional file 13: Table S4.

### *Xenopus* experiments

In vitro fertilization, embryo culture, and culture of explants were carried out as described [57]. Staging was done according to Nieuwkopp [58]. mRNA was produced with the mMessage mMachine SP6 trancription Kit (Ambion) from the *HyWnt3*, *XWnt8*, *HAS-7*, and flag-tagged *GFP* (control mRNA) ORFs in the respective linearized pCS2+ vectors. mRNA was purified with a phenol/chloroform extraction and a subsequent isopropanol precipitation. Injections were done into the marginal zone of both ventral cells of the 4-cell stage. Total amounts of each injected mRNA were *XWnt8* (10 pg), *HyWnt3* (10 or 100 pg), *HyWnt3*/*HAS-7* (100 pg each), and scrambled RNA (100 pg).

### Structural modeling

Protein-Protein docking experiments were performed in ClusPro2 [59] using the crystal structures of *Xenopus* Wnt8 (pdb-code:4F0A) and promeprin ß (pdb-code: 4GWM). The propetide E25-G66 blocking access to the active site cleft and disordered loop segments in Wnt8 were removed to allow for proper structural analysis. Targeted search matrices were chosen by selecting the zinc-binding site of promeprin ß (H132-H136-H142) and the putative cleavage site of HyWnt3 (K186-D187-P188) as attractive search targets. Based on Lennart-Jones potentials, distance metrices, and electrostatic evaluations the best 100 docking hits were subject to gradient energy minimization in the Crystallography and NMR system. 14 The lowest energy structures were further subject to 500 cycles of unrestrained Powell minimization. Harmonic restraints were imposed on the target molecule (2 kcal/mol Å^2^) with increased weight (25 kcal/mol Å^2^). Protein structure and model assessment tools were used to verify the quality of the modeled structure. Additionally, HAS-7 modeling was refined using Modeller [60] implemented in Chimera [61] with astacin complexed to a transition state analog inhibitor (pdb-code: 1QJI) and zebrafish hatching enzyme (3LQB), the latter being the most closely HAS-7-related astacin with known structure to date.

### Phylogenetic analysis

Amino acid sequence alignments and phylogenetic trees were computed using the SEAVIEW package (http://doua.prabi.fr/software/seaview) [62, 63]. Alignments were performed by CLUSTAL omega embedded in SEAVIEW [64] using the default adjustments. Refinements of the S1’ regions were arranged based on overlays of the X-ray crystal structures of crayfish astacin (pdb-codes 1ast, 1qji), and zebrafish hatching enzyme 1 (ZHE1, pdb-code 3lqb). Phylogenetic tree calculations were performed using the maximum likelihood approach provided by the PhyML tool implemented in SEAVIEW [65].

### Mathematical model

To obtain mechanistic insights into the regulatory function of HAS-7, we propose a mathematical model given in terms of reaction-diffusion equations that model signaling processes. To account for a realistic geometry of the *Hydra* tissue bilayer, we adopt a mechano-chemical modeling approach coupling the morphogen dynamics with the evolution of infinitely thin deforming tissue [66, 67]. The mechanical part is given by a 4th order partial differential equation (PDE) model based on the minimization of the Helfrich-type energy. It allows describing small tissue deformations induced by patterns of gene expression, such as the initial stage of tentacle development.

The point of departure for the molecular interaction model is the HyWnt3/beta-Catenin model recently proposed in [68], describing three separate (but interacting) pattern formation systems for the body axis (including beta-Catenin/TCF), the head organizer (including HyWnt3), and the tentacle system. In this paper, we extend the HyWnt3/beta-Catenin model to account for experimentally investigated HyWnt3-HAS-7 interactions.

The core of the model accounts for the dynamics of Wnt3 and beta-Catenin/TCF signaling that are coupled through the canonical Wnt signaling pathway [6, 69]. Although it is frequently assumed that beta-Catenin/TCF and Wnt3 molecules act in the confines of the same pattern formation system to coordinate body axis and head formation, e.g., [10, 31, 70, 71], we distinguish between them in the model and describe the dynamics of Wnt3 and beta-Catenin using two model variables, respectively, each controlled by Turing-type activator-inhibitor loops [29, 30]. Although accounting explicitly for HyWnt3 and beta-Catenin/TCF, the proposed model involves hypothetical inhibitors that might be linked to HyDkk1/2/4 [68, 72], the Sp5 transcription factor [10], or Thrombospondin (HmTSP) [12]. Since Sp5 does not diffuse, it is not a HyWnt3 inhibitor in the sense required by the classical activator-inhibitor model. An additional model of the molecular mechanism, possibly including a hypothetical long-range factor locally activating Sp5 is beyond the scope of this work. Therefore, we phenomenologically summarize HyWnt3 inhibition by a diffusive HyWnt3 inhibitor possibly including Sp5 and HmTSP. Similar dynamics may result from multistability in the intracellular signaling [73–75], or a negative feedback loop stemming from mechano-chemical interactions [66, 76]. The reduced mathematical representation of the underlying mechanisms is sufficient for the purpose of this paper, since we focus on the role of HAS-7 in the pattern formation process. Specifically, we clarify its function in pattern selection (localization of the Wnt3 spot) that does not depend on a particular molecular agent of the Wnt3/beta-Catenin pattern formation mechanism. The modular structure of the model suggests robustness of the underlying mechanisms that is controlled in a stepwise process.

The HAS-7 function is modeled by coupling the HyWnt3/beta-Catenin model to the dynamics of HAS-7 that is positively regulated by beta-Catenin. The indirect regulation is modeled as a transcriptional HAS-7 activation by head-specific molecules downstream of the organizer (some candidates of the latter are presented in Ref. [7]). The assumption is motivated by (1) our HAS-7 promoter analysis, (2) the HAS-7 expression patterns after AZK treatment presented in this study, and (3) the natural mechanistic assumption that HAS-7 should be activated as soon as a head is established in order to suppress the formation of additional heads. Furthermore, we assume that HAS-7 degrades Wnt3 ligands and that Wnt3 negatively regulates HAS-7. The latter is motivated by (1) our observation that HAS-7 transcript is absent from the upper hypostome, and (2) the natural mechanistic assumption that HAS-7 suppresses organizer formation in the surroundings but not of the existing organizer itself. It is not known if the local negative regulation of HAS-7 by the hypostome is governed directly by Wnt3 or by other molecules downstream of Wnt3. Nevertheless, accounting for such modification of the regulatory mechanism does not influence the results of the model. Hence, for the sake of simplicity, we assume a local negative regulation of HAS-7 by Wnt3.

Model variables are biologically defined as follows: *β_cat*: Nuclear beta-Catenin/TCF; *β_cat*_*ant*_: *β_cat* antagonist, probably involving Dickkopf1/2/4-C [32, 72]; *Wnt*3: HyWnt3; *Wnt*3_*ant*_: HyWnt3 antagonist (probably Sp5 [10] and HmTSP [12] involved); *Head:* Head-related factors downstream of HyWnt3 (e.g., multiple Wnts [7]); *HAS*: HAS-7; *SD*: Source density (long-term storage of the head forming potential); *Tent*: Tentacle activator (probably HyAlx [77], Wnt8 [36], BMB5-8b [78] involved); *Tent*_*ant*_ Tentacle activator antagonist (unknown). Model equations are presented below.

1 $$ {\partial}_t\beta \_ ca t={a}_1{\Delta}^{\Gamma}\beta \_ ca t+{b}_1\cdot \left(1.0+{c}_1\cdot Wnt3\right)\cdot SD\cdot \frac{0.05+\beta \_{cat}^2}{\beta \_ ca{t}_{ant}}-{d}_1\cdot \beta \_ ca t $$

2 $$ {\partial}_t\beta \_{cat}_{ant}={a}_2{\Delta}^{\Gamma}\beta \_{cat}_{ant}+{b}_2\cdot \left(1.0+{c}_2\cdot Wnt3\right)\cdot SD\cdot \beta \_{cat}^2-{d}_2\cdot \beta \_{cat}_{ant} $$

3 $$ {\partial}_t Wnt3={a}_3{\Delta}^{\Gamma} Wnt3+{b}_3\cdot \beta \_{cat}_{ant}\cdot \frac{0.05+ Wnt{3}^2}{Wnt{3}_{ant}\cdot \left(1.0+{c}_3\cdot Wnt{3}^2\right)}-{d}_3\cdot \left(1.0+{e}_3\cdot HAS\right)\cdot Wnt3 $$

4 $$ {\partial}_t Wnt{3}_{ant}={a}_4{\Delta}^{\Gamma} Wnt{3}_{ant}+{b}_4\cdot \beta \_{cat}_{ant}\cdot \frac{0.005+ Wnt{3}^2}{1.0+{c}_4\cdot Wnt{3}^2}+0.035-{d}_4\cdot Wnt{3}_{ant} $$

5 $$ {\partial}_t Head={a}_5{\Delta}^{\Gamma} Head+{b}_5 Wnt3-{d}_5 Head $$

6 $$ {\partial}_t HAS={a}_6{\Delta}^{\Gamma} HAS+\frac{b_6 Head}{1.0+{c}_6\cdot Wnt3}-{d}_6 HAS $$

7 $$ {\partial}_t Tent={a}_7{\Delta}^{\Gamma} Tent+\frac{b_7\cdot SD\cdot \left(0.005+{ Ten t}^2\right)}{Ten{t}_{ant}\cdot \left(1+{c}_7\cdot { Ten t}^2\right)\cdot \left(1+{e}_7\cdot {Head}_{ant}\right)}-{d}_7\cdot Tent $$

8 $$ {\partial}_t{Tent}_{ant}={a}_8{\Delta}^{\Gamma}{Tent}_{ant}+\frac{b_8\cdot SD\cdot \left(0.005+{Tent}^2\right)}{\left(1+{c}_8\cdot {Tent}^2\right)\cdot \left(1+{e}_8\cdot {Head}_{ant}\right)}+0.014-{d}_8\cdot {Tent}_{ant} $$

9 $$ {\partial}_t SD={a}_9{\Delta}^{\Gamma} SD+b9\cdot \beta \_ cat+0.00003-{d}_9 SD $$

We performed numerical simulations of the model to obtain insights into the dependence of the model results on specific choices of parameters. Our study suggests that most of the parameters involved in Eq. (1)–(9) do not influence critically the qualitative HAS-related simulation results (Fig. 7a). In particular, most of them control specific properties of one of the three interplaying de novo pattern formation systems, such as spatial scaling of the pattern (size of expression domain), spacing between the maxima of the pattern, or a condition for de novo patterning. Following these simulation results, we fixed most of the parameters to values taken from Ref. [31, 32]. This allowed focusing on parameters accounting for the novel aspects of the model such as (i) interactions between beta-Catenin and Wnt3 that govern pattern formation on different spatial scales, and (ii) a feedback loop between HAS-7 and Wnt3. In general, changing the corresponding parameters, we observed robust model dynamics (qualitatively the same pattern formation). The only discrepancy was observed in simulations of siHAS-7/AZK animals, where the number of ectopic axes in model simulations depends on the strength of HAS-7 dependent degradation of Wnt3. This observation suggests that there might be an additional mechanism (possibly involving other members of the HAS family) ensuring an experimentally observed robustness with respect to the number of organizers.

For simulations of the unperturbed system, we applied the following parameters following Ref. model [31, 32, 68]):

$$ {a}_1=9\times 1{0}^{-5},{b}_1=3\times 1{0}^{-3},{c}_1=3\times 1{0}^{-2},{d}_1=3\times 1{0}^{-3}, $$

$$ {a}_2=11\times 1{0}^{-3},{b}_2=3\times 1{0}^{-3},{c}_2=3\times 1{0}^{-2},{d}_2=4\times 1{0}^{-3}, $$

$$ {a}_3=6\times 1{0}^{-5},{b}_3=7\times 1{0}^{-3},{c}_3=3\times 1{0}^{-3},{d}_3=12\times 1{0}^{-2},{e}_3=1\times 1{0}^2, $$

$$ {a}_4=24\times 1{0}^{-3},{b}_4=1\times 1{0}^{-2},{c}_4=3\times 1{0}^{-3},{d}_4=18\times 1{0}^{-2}, $$

$$ {a}_5=25\times 1{0}^{-3},{b}_5=1\times 1{0}^0,{d}_5=1\times 1{0}^{-2}, $$

$$ {a}_6=25\times 1{0}^{-3},{b}_6=1\times 1{0}^{-1},{c}_6=1\times 1{0}^1,{d}_6=5\times 1{0}^{-2}, $$

$$ {a}_7=25\times 1{0}^{-5},{b}_7=2\times 1{0}^{-3},{c}_7=12\times 1{0}^{-2},{e}_7=3\times 1{0}^{-2},{d}_7=2\times 1{0}^{-2}, $$

$$ {a}_8=27\times 1{0}^{-3},{b}_8=3\times 1{0}^{-3},{c}_8=12\times 1{0}^{-2},{e}_8=3\times 1{0}^{-2},{d}_8=3\times 1{0}^{-2}, $$

$$ {a}_9=11\times {1}^{-5},{b}_9=3\times 1{0}^{-5},{d}_9=3\times 1{0}^{-5}. $$

To approximate the geometry of the *Hydra* tissue, initial conditions for *X*_1_, *X*_2_, and *X*_3_ are parametrized over a closed 2D unit-sphere *S*^2^ embedded in 3D space with *X*_1_(*t* = 0) *≡ X*_2_(*t* = 0) *≡* 0 and *X*_3_(*t* = 0) = 4 *· s*_3_, thus, leading to a stretch into the direction of *s*_3_ (given that *s*_1_*, s*_2_*, s*_3_ are Eulerian coordinates of the *S*^2^-surface). For biological molecules, we used a stochastic initial distribution based on the standard random generator provided by C++. The source density is modeled using an initial gradient given by *SD*(*t* = 0) = 4*.*0 *·* (*exp*(*s*_3_)*/exp*(1))*.* Thus, in all simulations, only the geometric and chemical body axis gradient are initially prescribed.

In simulation of the AZK treatment, we modified the initial conditions for the source density adding an offset by *SD*(*t* = 0) = 2*.*0 + 4*.*0 *·* (*exp*(*s*_3_)*/exp*(1))*.* To simulate HAS knockdown, we set *b*_6_ = 0. For Dkk knockdown, we modeled a reduction of Dkk activity by increasing *d*_2_ by the two-fold. In the model, a complete deactivation of *beta_cat*_*ant*_ prevents creation of any pattern, since the body-scale system is described by just two components that are a minimal set required for pattern formation. Finally, Wnt3 overexpression was simulated by adding the constant *c* = 0*.*1 to the production.

## Supplementary Information

**Additional file 1: Fig. S1.** Ion exchange chromatogram of hydra head lysate pool. (a) 7 fractions of 0.5 ml exceeding an absorption unit threshold of 0.175 were collected as indicated. The cut-off was chosen to provide a critical total protein concentration (> 80 μg) for the subsequent proteome analysis. (b) Peak fractions from (a) were re-screened for HyWnt3-His processing activity. A fragment of *Hydra* cadherin extracellular domain comprising the first two N-terminal cadherin repeats (HmCadherin1-2) was used as control substrate to monitor unspecific matrix metalloproteinase activity. Accordingly, fractions 4-5 were pooled and analyzed by mass spectrometry as HyWnt3-His(+) sample, fractions 6-7 as HyWnt3-His(-) sample.

**Additional file 2: Table S1.** (a) Secretome of *Hydra* HL HyWnt3 (+) fraction. (b) Secretome of *Hydra* HL HyWnt3 (-) fraction.

**Additional file 3: Table S2.** Complete proteome data of HyWnt3(+) and HyWnt3(-) HL fractions.

**Additional file 4: Fig. S2.** Phylogenetic tree of astacin metalloproteinases established by PhyLM 3.0 (SEAVIEW package) and based on an alignment of the catalytic domains only, omitting pro-sequences and multiple C-terminal domains. Numbers indicate probability values (in %) obtained from 100 bootstrap replications. Protein abbreviations from bottom: fAST, flavastacin (*Flavobacterium meningosepticum*, i.e. *Chryseobacterium meningosepticum*, i.e. *Elisabethkingia meningoseptica*, Q47899, used as outgroup); HEA-1, *Hydractinia echinata* astacin-1 (Q2MCX9); HEA-3, *H. echinata* astacin-3 (Q2MCX7); HEA-4, *H. echinata* astacin-4 (Q2MCX6); HMP1, *Hydra vulgaris* metalloproteinase-1 (NP_001296695.1), AST, astacin (*Astacus astacus*, P07584); ALV, alveolin (*Oryzias latipes*, Q9IBE7); ZHE-1, zebrafish hatching enzyme-1 (*Danio rerio*, Q1LW01); HCE-1, high choreolytic enzyme-1 (*O. latipes*, P31580); zNEP, nephrosin (*Danio rerio*, A2VD22); cNEP, nephrosin (*Cyprinus carpio*, O42326); hOVA, ovastacin (*H. sapiens*, Q6HA08); mOVA, ovastacin (*Mus musculus*, Q6HA09); TLD, tolloid (*Drosophila melanogaster*, P25723); TLL1, tolloid-like 1 (*H. sapiens*, O43897); TLL2, tolloid-like 2 (*H. sapiens*, Q9Y6L7); BMP1, bone morphogenetic protein-1 (*H. sapiens*, P13497); NAS-35, nematode astacin-35 (*C. elegans*, P98060); SPAN, *Strongylocentrotus purpuratus* astacin (P98068); hMEPβ, meprin β (*H. sapiens*, Q16820); mMEPβ, meprin β (*M. musculus*, Q61847); zMEPβ, meprin β (*D. rerio*, B8JKC0); hMEPα, meprin α (*H. sapiens*, Q16819), mMEPα, meprin α (*M. musculus*, P28825); zMEPa1, meprin α1 (*D. rerio*, Q5RHM1); zMEPα2, meprin α2 (*D. rerio*, F1QRQ5); HMP2, *H. vulgaris* metalloproteinase 2, NP_001296695.1); HAS-9, *H. vulgaris* astacin-9 (XP_002161766.1); HAS-8, *H. vulgaris* astacin-8 (XP_002153855.1); HAS-10, *H. vulgaris* astacin-10 (XP_002159980.2); HAS-7, *H. vulgaris* astacin-7 (XP_012560086.1); HAS-11, *H. vulgaris* astacin-11 (XP_012561076.1); HAS-1, *H. vulgaris* astacin-1 (XP_012565441.1); HEA-2, *H. echinata* astacin-2 (Q2MCX8); PMP1, *Podecoryne carnica* metalloproteinase-1 (O62558); HAS-4, *H. vulgaris* astacin-4 (XP_002162738.1); HAS-2, *H. vulgaris* astacin-2 (XP_002162822.1); HAS-6, *H. vulgaris* astacin-6 (XP_002157397.2); HAS-5, *H. vulgaris* astacin-5 (XP_002164800.1); HAS-3, *H. vulgaris* astacin-3 (XP_002166229.3).

**Additional file 5: Fig. S3.** Expression of *HAS* genes in the interstitial stem cell cluster. (a) t-SNE representation of interstitial cells with clusters labeled by cell state as presented in [25]. (b) Interstitial cell cluster annotation of *HyDkk1/2/4* and ataxin genes identified in HyWnt3(+) head lysate fraction. The cells in the t-SNE plots were colored based on expression levels for the respective gene. The transcript IDs are as follows: *HMP1*: t1098aep, *HAS-1*: t20535aep, *HAS-2*: t18494aep, *HAS-3*: t22149aep, *HAS-4*: t11453aep, *HAS-5*: t596aep, *HAS-6*: t19593aep, *HAS-7*: t16296aep, *HAS-8*: t22154aep, *HAS-9*: t3416aep, *HAS-10*: t10258aep, *HAS-11*: t19316aep. *HyDkk1/2/4*: t8678aep. Cluster label abbreviation key: bat: battery cell, fmgl: female germ-line, gc: gland cell, gmgc: granular mucous gland cell, hyp: hypostome, id: integration doublet, mgl: male germline, nb: nematoblast, nc: neuronal cell, nem: nematocyte, nurse: nurse cells prog: progenitor, SC: stem cell, smgc: spumous mucous gland cell, zmg: zymogen gland cell. Numbers indicate different cell populations within a cluster. (c) Microscopic image showing the epithelial bilayer of the upper gastric region of *Hydra*. *HAS-7* WISH marks gland cells interspersed between the endodermal epithelial cells that are aligned to the central mesoglea (M) separating endo (En)- and ectoderm (Ec). Bar = 20 μM.

**Additional file 6: Fig. S4.** Detection of HAS-7 by Western blot. (a) Antigenic peptide competition demonstrates the specificity of the HAS-7 antibody. A Western blot for tissue lysates as in Fig. 3a was performed using primary antibody solution with (right panel) or without (left panel) 1 mg/ml of the antigenic peptide used for generating the HAS-7 antibody. The HAS-7 peptide effectively reduces the detection of specific bands at ~ 40 and 70 kDa. (b) Ni-NTA affinity purified recombinant HAS-7. Separation by 12% SDS-PAGE was followed by staining with Coomassie brilliant blue (left) or transfer to PVDF and immunodetection (right) using the Penta-His-antibody as described above. For each lane 1.8 μg of recombinant HAS-7 protein eluted with 250 mM imidazole were applied. M, marker proteins as indicated. (c) Dilution series of recombinant HAS-7 and native HAS-7 protein in HL detected with anti-HAS-7 antibody show a double band at 42 kDa for recombinant HAS-7 while in the HL two double bands migrating at 38 kDa and 42 kDa, respectively, are detectable. This heterogenous pattern likely represents a mixture of immature and posttranslationally modified forms of HAS-7 proteins in the tissue lysate. (d) Upper panel: Western blot detection of HAS-7 in full hydra lysate and elution fractions after cation exchange chromatography as indicated. Fractions 15-21 were pooled as they showed a subcritical protein concentration (< 1 mAU). The prominent band at about 30 kDa detected in fraction 22 likely represents the mature protease lacking the pro-domain. Lower panel: detection of HyWnt3-His incubated for 6 hours with the indicated fractions shows highest proteolytic activity for fraction 22.

**Additional file 7: Fig. S5.** Evidence for normal function and morphology of ectopic heads and tentacles. (a-b) Both heads in a *HAS-7* siRNA treated animal with a double axis are able to capture and feed on artemia. The arrow denotes an ectopic foot induced by the secondary head. Scale bars = 500 μm. (c-e) Ectopic tentacles induced by ALP treatment show anatomic and molecular features of functional tentacles as demonstrated by immunocytochemistry using a nematocyst-specific antibody (anti-CPP-1) [53]. CPP-1 is a structural component of mature nematocysts in battery cells of tentacles. (c) Overview of CPP-1-stained hydra with ectopic tentacles. Scale bar = 200 μm. (d-e) Enlargement from boxed are in c shows mature stenotele type nematocysts in the ectodermal epithelial layer of the tentacle as visualized by differential interference contrast (d) and corresponding fluorescence image (e). Arrows indicate stenoteles. Scale bars = 20 μm.

**Additional file 8: Fig. S6.** Representative images of knockdown and transgenic phenotypes. Representative images of *HAS-7* siRNA treated (a-c), *HAS-7* siRNA/AZK treated (d-g) or transgenic actin::HyWnt3 (h-i) animals. Scale bars: 200 μm. The inset in Fig. S6a shows an early stage of ectopic axis formation recorded 4 days after electroporation. Red arrows indicate the hypostome areas of the two heads.

**Additional file 9: Fig. S7.** Function of HAS-7 in regeneration. Animals bisected after *HAS-7* siRNA electroporation do not show axis duplication in head (a-b) or foot (c-d) regenerates. Animals were bisected at 50% of body length at day 6 after electroporation and documented at day 0 (a, c) and day 4 (b, d) after bisection. Representatives of 25 bisected hydras examined. Scale bars: 200 μm. (e) Heat map showing the dynamics of transcript levels for HyWnt3(+) astacin genes compared to *HyWnt3* and *beta-Catenin*. Only components that were significantly differentially expressed (*P* < 0.05) at one or more time points are shown. The analysis is based on head regeneration transcriptome data published in Petersen et al. [34]. Transcript IDs were obtained by BLAST searches of the *Hydra* head regeneration transcriptome assemblies on https://research.nhgri.nih.gov/hydra (HAS-1: comp9275_c0_seq1; HAS-2: comp23661_c0_seq1; HAS-3: comp16657_c0_seq1; HAS-4: comp9917_c0_seq1; HAS-5: comp19153_c0_seq1; HAS-6: comp21556_c0_seq1; HAS-7: comp23282_c0_seq1; HAS-8: comp23009_c0_seq1; HAS-9: comp16628_c0_seq1; HAS-10: comp22419_c0_seq1; HAS-11: comp8104_c0_seq1; HMP1: comp18584_c0_seq1; HyWnt3: comp17763_c0_seq; beta-Catenin: comp12447_c0_seq1).

**Additional file 10: Fig. S8.** Developmental balance between ectopic structures. (a) siHAS-7/siNdr electroporation blocks ectopic axis formation after subsequent AZK treatment. (b-c) siHAS-7/Wnt8 electroporation and AZK treatment reduces ectopic tentacle development in double axis animals (b) and leads to multiple secondary axis formation in a fraction of the treated animals (c). Red arrows denote secondary axes. The asterisk denotes the peduncle region. (d) Ectopic tentacle inhibition is clearly evident in animals electroporated with siWnt8 followed by AZK treatment. Note that few residual ectopic tentacles are detectable in c and d mostly on the side not directly hit by the electroporation pulse. (e) Continuous treatment with a low concentration of ALP (0.2 μM) leads to secondary axis development after about 3 weeks. Note that as in siHAS-7/Wnt8 treated animals, few residual ectopic tentacles are present. Representatives of at least 7 hydras examined. Scale bars = 200 μm. (f) Ratios of double and multiple axis phenotypes in hydras after electroporation with siGFP or combinations of siRNAs as indicated. AZK incubation was started 6 days after electroporation and the numbers of ectopic axes in each group were counted 12 days after AZK removal. Total numbers of animals with ectopic axis phenotype in each group were: siGFP/AZK = 0/38 (*n*=3), siHAS-7/siNdr/AZK = 0/63 (n=3), siHAS-7/siWnt8/AZK = 22/61 (multiple axis = 7) (n=3), Results from at least three independent experiments are shown. Each column represents the total percentage of one group, bars indicate the mean ± S.E.M. **P value < 0.05. ns = not significant. The data were analyzed using an unpaired T-tests with Welch’s correction. The individual data values are shown in Additional file 14.

**Additional file 11: Fig. S9.** Uncropped Western blot and gel images

**Additional file 12: Table S3.** LNA and RNA probe sequences used for WISH.

**Additional file 13: Table S4.** siRNA and qPCR primer sequences.

**Additional file 14:** The individual data values for Figs. 3f and 5f.

## Data Availability

All data generated or analyzed during this study are included in this published article (and its supplementary information files). Raw data can be found in Additional file 14.

## Abbreviations

ALP: Alsterpaullone
AZK: 1-Azakenpaullone
BMP: Bone morphogenetic protein
ChIP: Chromatin immunoprecipitation
DFG: Deutsche Forschungsgemeinschaft
Dkk: Dickkopf
DMEM: Dulbecco’s modified Eagle’s medium
DMSO: Dimethylsulfoxid
FTMS: Fourier transform mass spectrometry
GFP: Green fluorescent protein
GSK-3: Glycogen synthase kinase 3
HAS: *Hydra* astacin
HPLC: High-performance liquid chromatography
HL: Head lysate
Hm: *Hydra magnipapillata*
HM: Hydra medium
HMP: Hydra metalloproteinase
iCRT: Inhibitor of beta-Catenin-responsive transcription
ISH: In situ hybridization
LNA: Locked nucleic acid
ORF: Open reading frame
PBS: Phosphate-buffered saline
PCR: Polymerase chain reaction
PDB: Protein data bank
RFP: Red fluorescent protein
RNAi: RNA interference
RT: Room temperature
SEC: Secretome
ShkT: Stichodactyla toxin
siRNA: Short interfering RNA
TCF: T cell factor
TES: 2-[Tris-(hydroxymethyl)methylamino]-1-ethane sulfonic acid
TSP: Thrombospondin
Wnt: Wingless/integrated
UPLC: Ultra-high-performance liquid chromatography
WISH: Whole mount in situ hybridization
X: *Xenopus*
