# Supplementary figures and images for: The Wnt-specific astacin proteinase HAS-7 restricts head organizer formation in Hydra
Source: BMC Biol. 2021 Jun 9;19:120. doi: 10.1186/s12915-021-01046-9 (PMC8191133; doi:10.1186/s12915-021-01046-9)

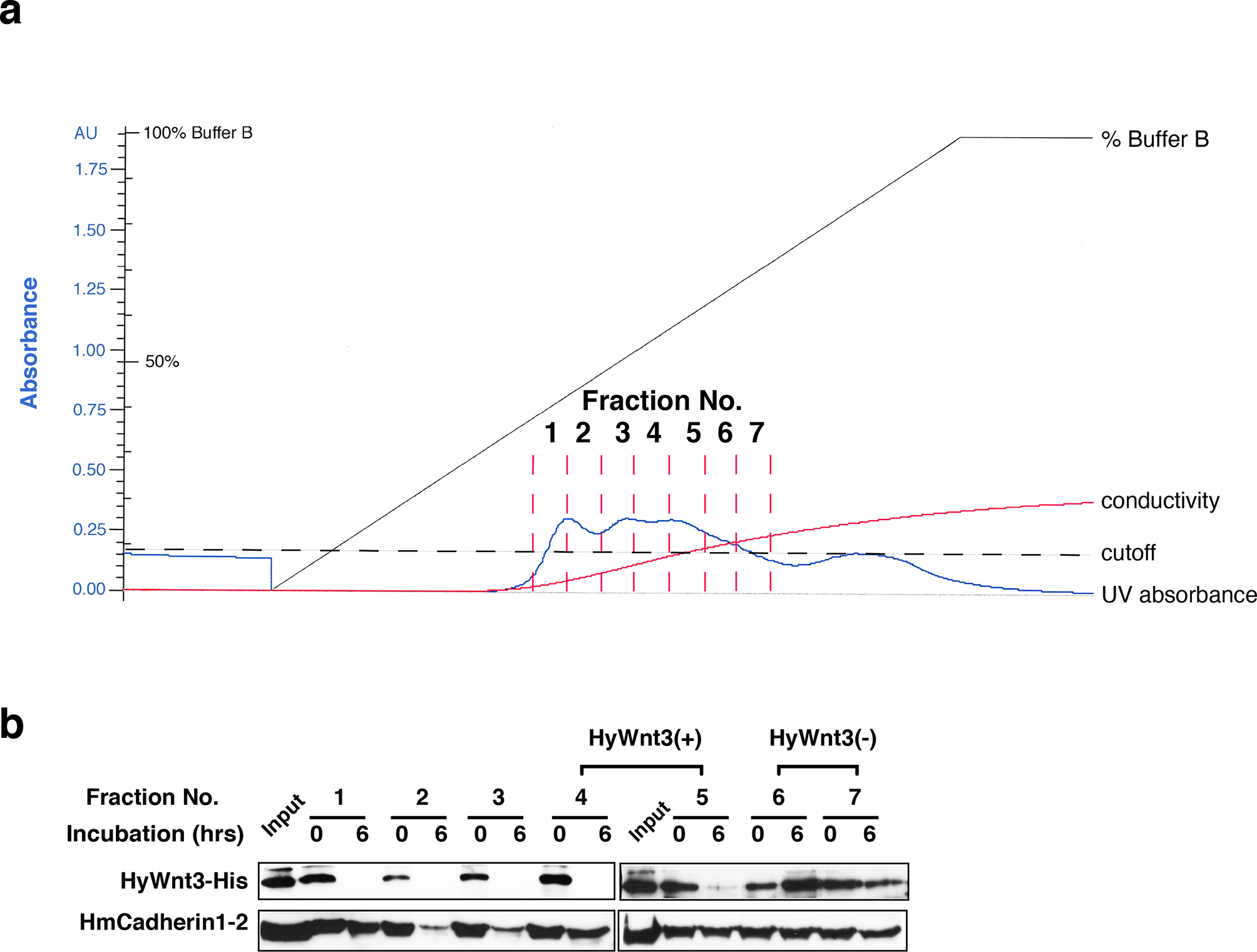

Supplement: Supplementary file 1 — Additional file 1: Fig. S1. Ion exchange chromatogram of hydra head lysate pool. (a) 7 fractions of 0.5 ml exceeding an absorption unit threshold of 0.175 were collected as indicated. The cut-off was chosen to provide a critical total protein concentration (> 80 μg) for the subsequent proteome analysis. (b) Peak fractions from (a) were re-screened for HyWnt3-His processing activity. A fragment of Hydra cadherin extracellular domain comprising the first two N-terminal cadherin repeats (HmCadherin1-2) was used as control substrate to monitor unspecific matrix metalloproteinase activity. Accordingly, fractions 4-5 were pooled and analyzed by mass spectrometry as HyWnt3-His(+) sample, fractions 6-7 as HyWnt3-His(-) sample. [file 12915_2021_1046_MOESM1_ESM.png]

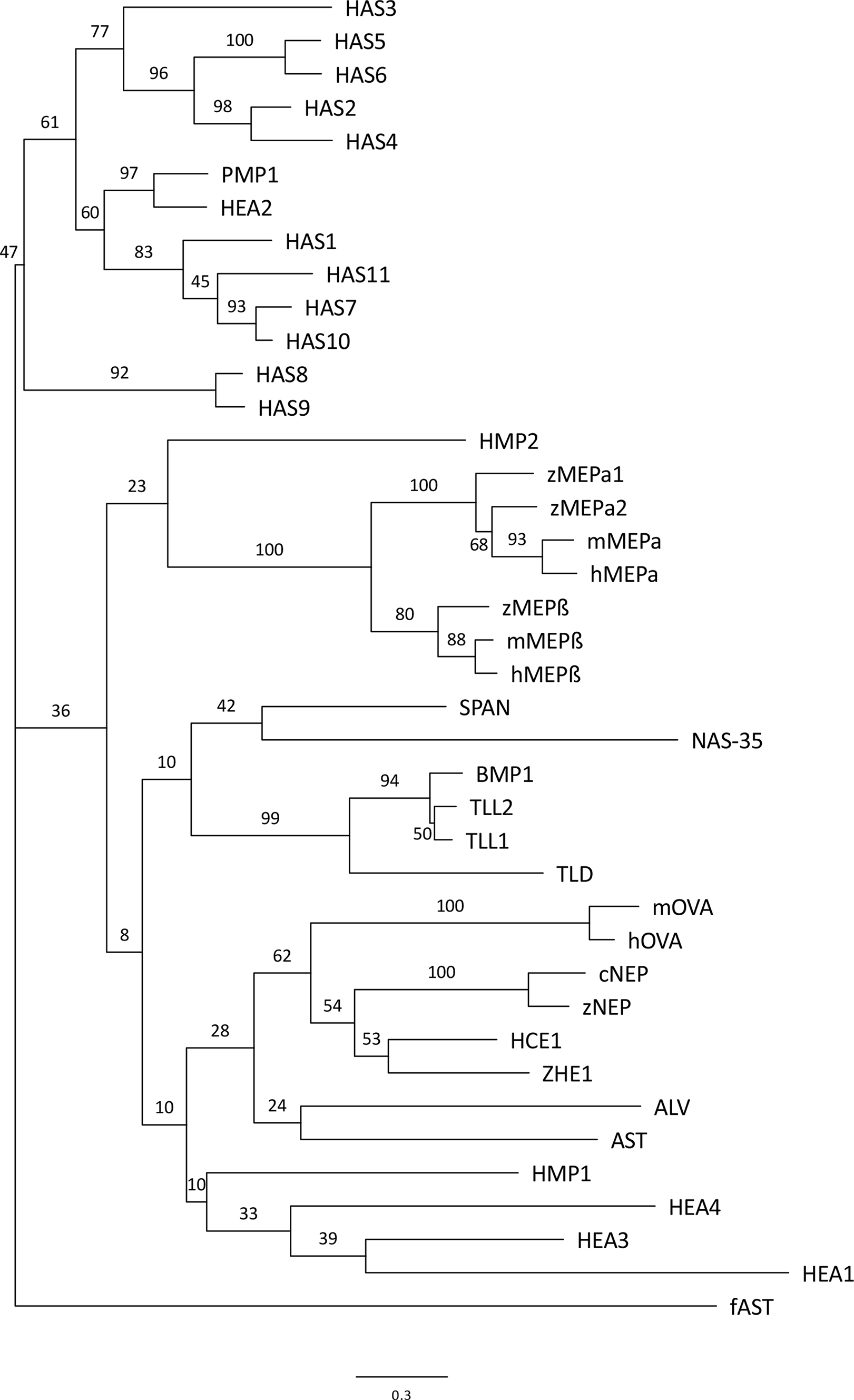

Supplement: Supplementary file 4 — Additional file 4: Fig. S2. Phylogenetic tree of astacin metalloproteinases established by PhyLM 3.0 (SEAVIEW package) and based on an alignment of the catalytic domains only, omitting pro-sequences and multiple C-terminal domains. Numbers indicate probability values (in %) obtained from 100 bootstrap replications. Protein abbreviations from bottom: fAST, flavastacin (Flavobacterium meningosepticum, i.e. Chryseobacterium meningosepticum, i.e. Elisabethkingia meningoseptica, Q47899, used as outgroup); HEA-1, Hydractinia echinata astacin-1 (Q2MCX9); HEA-3, H. echinata astacin-3 (Q2MCX7); HEA-4, H. echinata astacin-4 (Q2MCX6); HMP1, Hydra vulgaris metalloproteinase-1 (NP_001296695.1), AST, astacin (Astacus astacus, P07584); ALV, alveolin (Oryzias latipes, Q9IBE7); ZHE-1, zebrafish hatching enzyme-1 (Danio rerio, Q1LW01); HCE-1, high choreolytic enzyme-1 (O. latipes, P31580); zNEP, nephrosin (Danio rerio, A2VD22); cNEP, nephrosin (Cyprinus carpio, O42326); hOVA, ovastacin (H. sapiens, Q6HA08); mOVA, ovastacin (Mus musculus, Q6HA09); TLD, tolloid (Drosophila melanogaster, P25723); TLL1, tolloid-like 1 (H. sapiens, O43897); TLL2, tolloid-like 2 (H. sapiens, Q9Y6L7); BMP1, bone morphogenetic protein-1 (H. sapiens, P13497); NAS-35, nematode astacin-35 (C. elegans, P98060); SPAN, Strongylocentrotus purpuratus astacin (P98068); hMEPβ, meprin β (H. sapiens, Q16820); mMEPβ, meprin β (M. musculus, Q61847); zMEPβ, meprin β (D. rerio, B8JKC0); hMEPα, meprin α (H. sapiens, Q16819), mMEPα, meprin α (M. musculus, P28825); zMEPa1, meprin α1 (D. rerio, Q5RHM1); zMEPα2, meprin α2 (D. rerio, F1QRQ5); HMP2, H. vulgaris metalloproteinase 2, NP_001296695.1); HAS-9, H. vulgaris astacin-9 (XP_002161766.1); HAS-8, H. vulgaris astacin-8 (XP_002153855.1); HAS-10, H. vulgaris astacin-10 (XP_002159980.2); HAS-7, H. vulgaris astacin-7 (XP_012560086.1); HAS-11, H. vulgaris astacin-11 (XP_012561076.1); HAS-1, H. vulgaris astacin-1 (XP_012565441.1); HEA-2, H. echinata astacin-2 (Q2MCX8); PMP1, Podecor [file 12915_2021_1046_MOESM4_ESM.png]

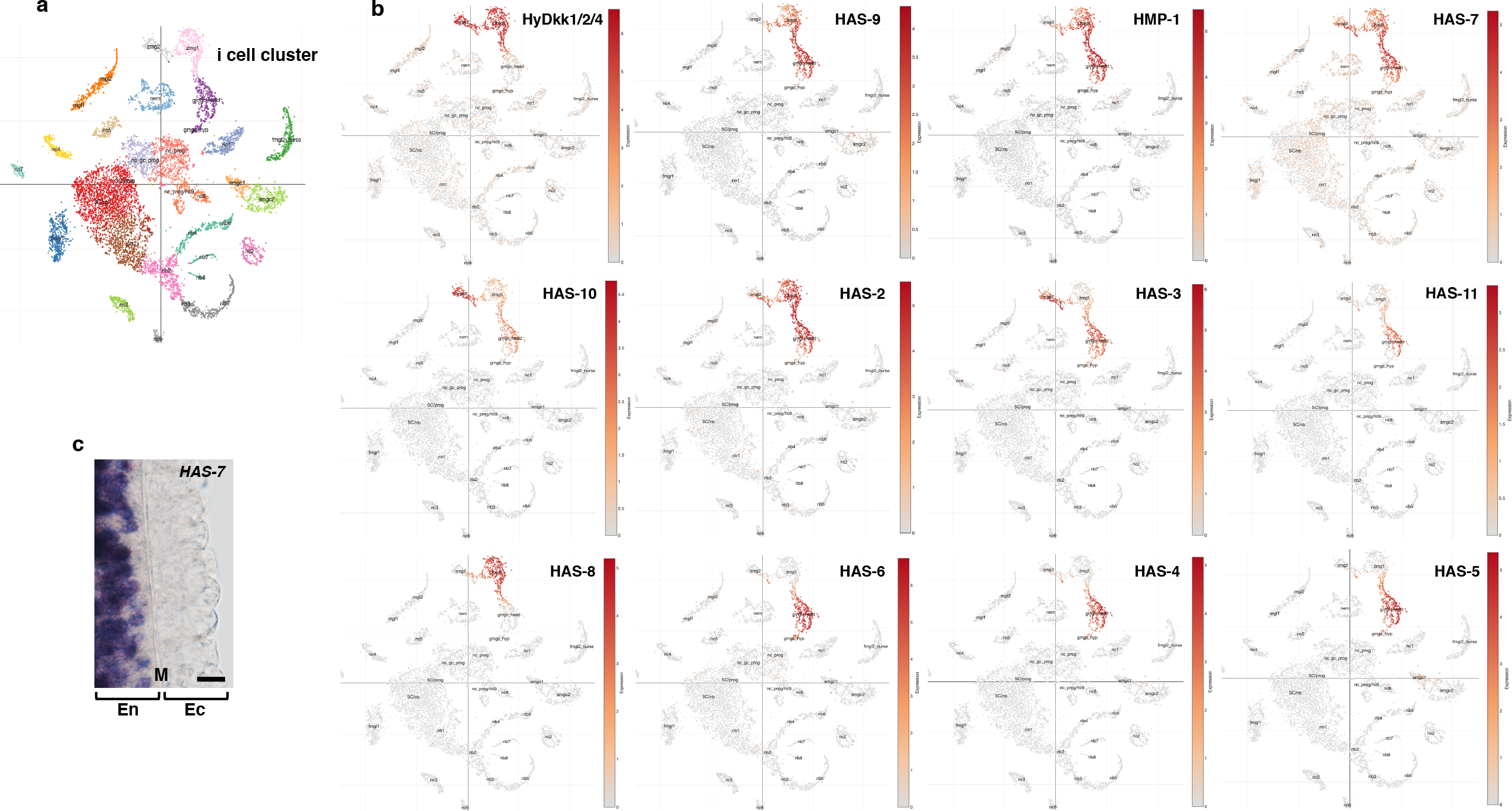

Supplement: Supplementary file 5 — Additional file 5: Fig. S3. Expression of HAS genes in the interstitial stem cell cluster. (a) t-SNE representation of interstitial cells with clusters labeled by cell state as presented in [25]. (b) Interstitial cell cluster annotation of HyDkk1/2/4 and ataxin genes identified in HyWnt3(+) head lysate fraction. The cells in the t-SNE plots were colored based on expression levels for the respective gene. The transcript IDs are as follows: HMP1: t1098aep, HAS-1: t20535aep, HAS-2: t18494aep, HAS-3: t22149aep, HAS-4: t11453aep, HAS-5: t596aep, HAS-6: t19593aep, HAS-7: t16296aep, HAS-8: t22154aep, HAS-9: t3416aep, HAS-10: t10258aep, HAS-11: t19316aep. HyDkk1/2/4: t8678aep. Cluster label abbreviation key: bat: battery cell, fmgl: female germ-line, gc: gland cell, gmgc: granular mucous gland cell, hyp: hypostome, id: integration doublet, mgl: male germline, nb: nematoblast, nc: neuronal cell, nem: nematocyte, nurse: nurse cells prog: progenitor, SC: stem cell, smgc: spumous mucous gland cell, zmg: zymogen gland cell. Numbers indicate different cell populations within a cluster. (c) Microscopic image showing the epithelial bilayer of the upper gastric region of Hydra. HAS-7 WISH marks gland cells interspersed between the endodermal epithelial cells that are aligned to the central mesoglea (M) separating endo (En)- and ectoderm (Ec). Bar = 20 μM. [file 12915_2021_1046_MOESM5_ESM.png]

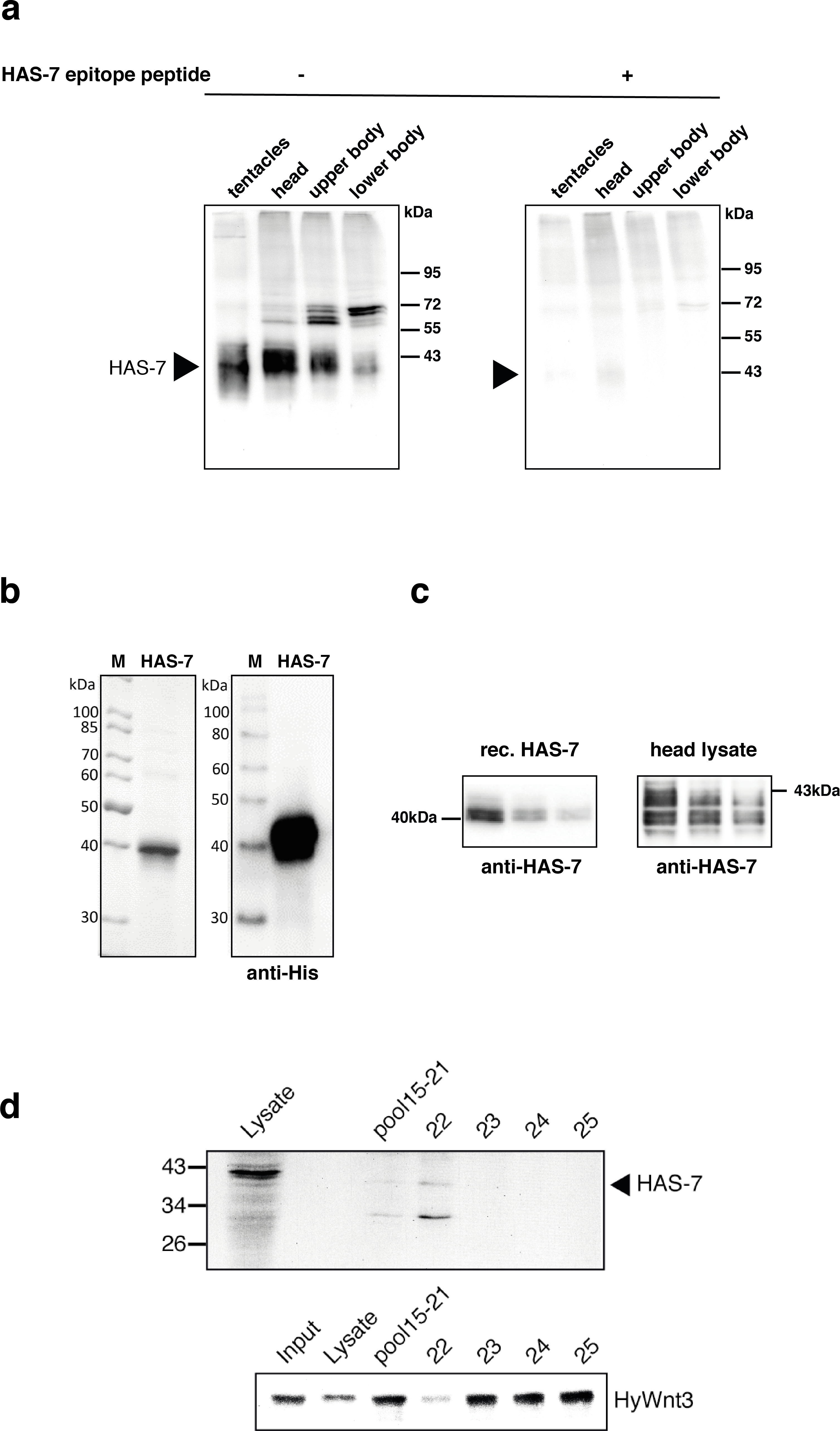

Supplement: Supplementary file 6 — Additional file 6: Fig. S4. Detection of HAS-7 by Western blot. (a) Antigenic peptide competition demonstrates the specificity of the HAS-7 antibody. A Western blot for tissue lysates as in Fig. 3a was performed using primary antibody solution with (right panel) or without (left panel) 1 mg/ml of the antigenic peptide used for generating the HAS-7 antibody. The HAS-7 peptide effectively reduces the detection of specific bands at ~ 40 and 70 kDa. (b) Ni-NTA affinity purified recombinant HAS-7. Separation by 12% SDS-PAGE was followed by staining with Coomassie brilliant blue (left) or transfer to PVDF and immunodetection (right) using the Penta-His-antibody as described above. For each lane 1.8 μg of recombinant HAS-7 protein eluted with 250 mM imidazole were applied. M, marker proteins as indicated. (c) Dilution series of recombinant HAS-7 and native HAS-7 protein in HL detected with anti-HAS-7 antibody show a double band at 42 kDa for recombinant HAS-7 while in the HL two double bands migrating at 38 kDa and 42 kDa, respectively, are detectable. This heterogenous pattern likely represents a mixture of immature and posttranslationally modified forms of HAS-7 proteins in the tissue lysate. (d) Upper panel: Western blot detection of HAS-7 in full hydra lysate and elution fractions after cation exchange chromatography as indicated. Fractions 15-21 were pooled as they showed a subcritical protein concentration (< 1 mAU). The prominent band at about 30 kDa detected in fraction 22 likely represents the mature protease lacking the pro-domain. Lower panel: detection of HyWnt3-His incubated for 6 hours with the indicated fractions shows highest proteolytic activity for fraction 22. [file 12915_2021_1046_MOESM6_ESM.png]

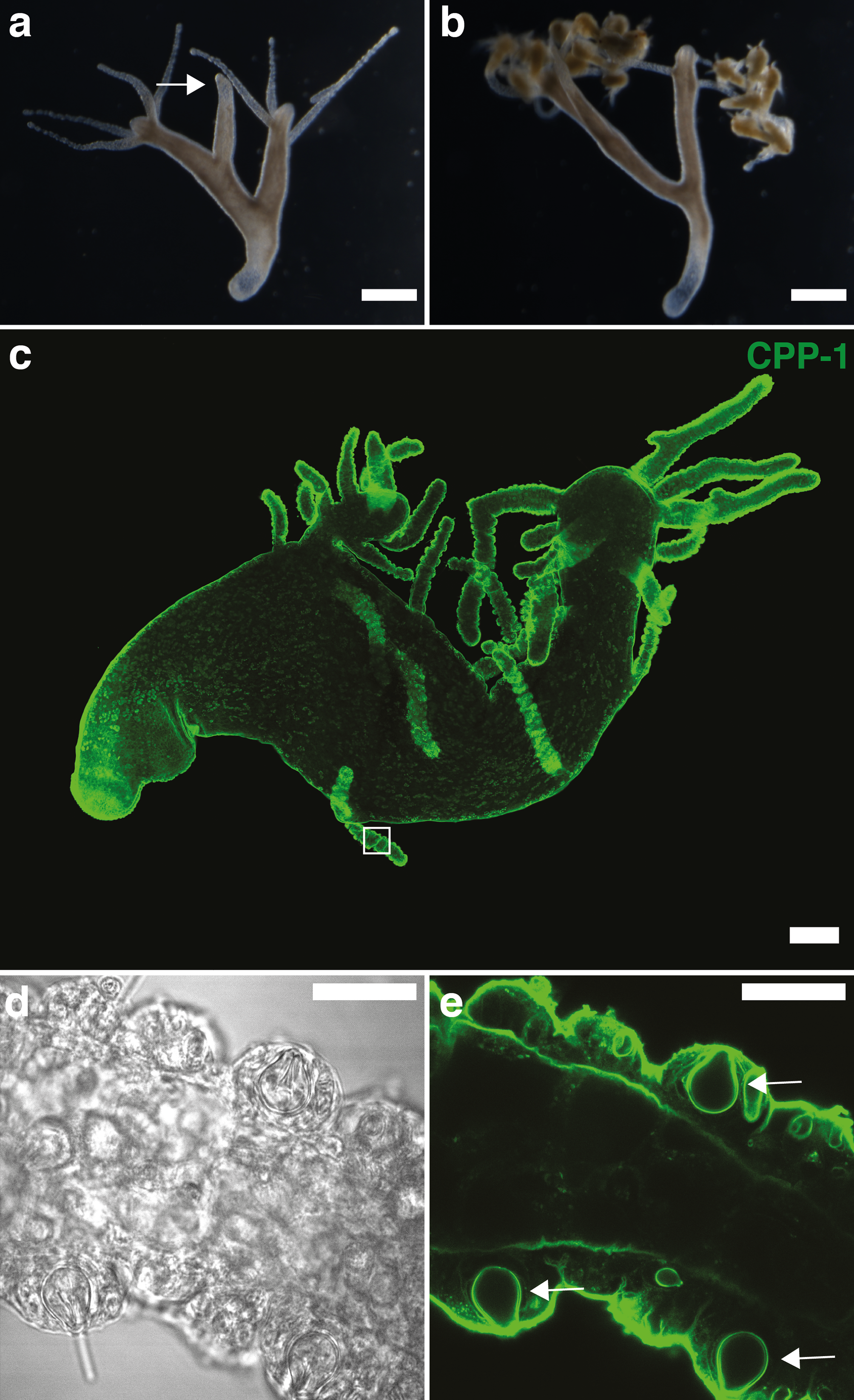

Supplement: Supplementary file 7 — Additional file 7: Fig. S5. Evidence for normal function and morphology of ectopic heads and tentacles. (a-b) Both heads in a HAS-7 siRNA treated animal with a double axis are able to capture and feed on artemia. The arrow denotes an ectopic foot induced by the secondary head. Scale bars = 500 μm. (c-e) Ectopic tentacles induced by ALP treatment show anatomic and molecular features of functional tentacles as demonstrated by immunocytochemistry using a nematocyst-specific antibody (anti-CPP-1) [53]. CPP-1 is a structural component of mature nematocysts in battery cells of tentacles. (c) Overview of CPP-1-stained hydra with ectopic tentacles. Scale bar = 200 μm. (d-e) Enlargement from boxed are in c shows mature stenotele type nematocysts in the ectodermal epithelial layer of the tentacle as visualized by differential interference contrast (d) and corresponding fluorescence image (e). Arrows indicate stenoteles. Scale bars = 20 μm. [file 12915_2021_1046_MOESM7_ESM.png]

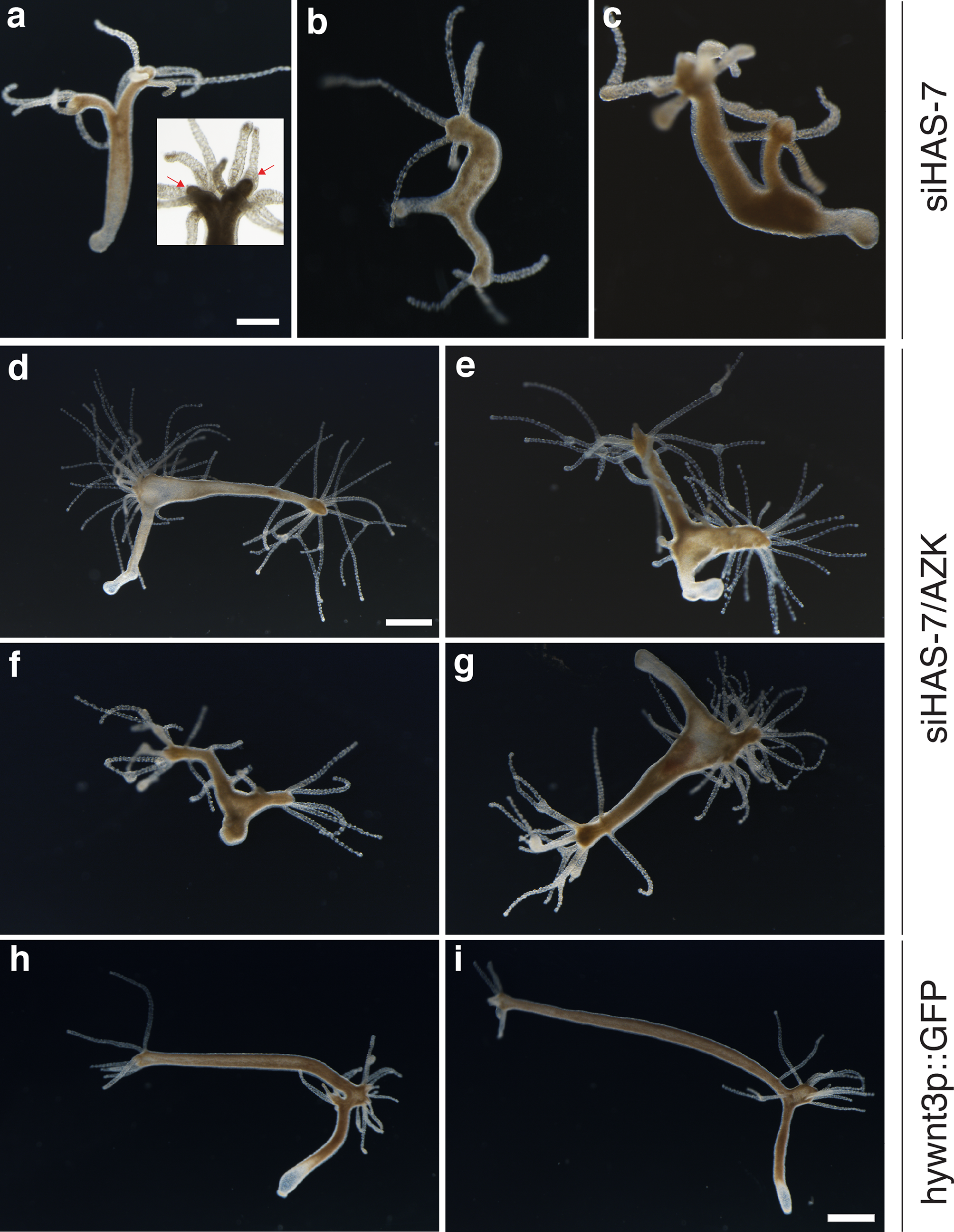

Supplement: Supplementary file 8 — Additional file 8: Fig. S6. Representative images of knockdown and transgenic phenotypes. Representative images of HAS-7 siRNA treated (a-c), HAS-7 siRNA/AZK treated (d-g) or transgenic actin::HyWnt3 (h-i) animals. Scale bars: 200 μm. The inset in Fig. S6a shows an early stage of ectopic axis formation recorded 4 days after electroporation. Red arrows indicate the hypostome areas of the two heads. [file 12915_2021_1046_MOESM8_ESM.png]

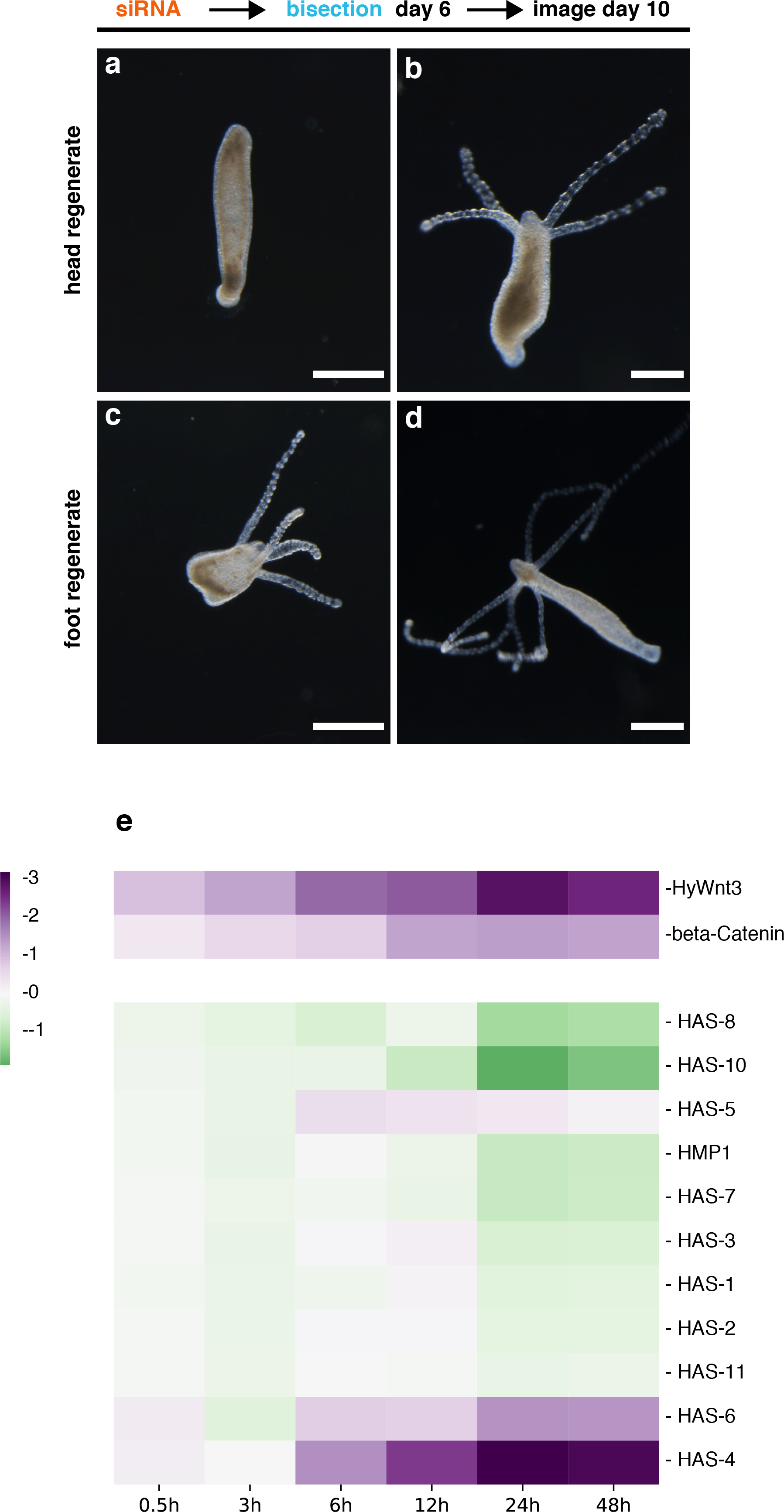

Supplement: Supplementary file 9 — Additional file 9: Fig. S7. Function of HAS-7 in regeneration. Animals bisected after HAS-7 siRNA electroporation do not show axis duplication in head (a-b) or foot (c-d) regenerates. Animals were bisected at 50% of body length at day 6 after electroporation and documented at day 0 (a, c) and day 4 (b, d) after bisection. Representatives of 25 bisected hydras examined. Scale bars: 200 μm. (e) Heat map showing the dynamics of transcript levels for HyWnt3(+) astacin genes compared to HyWnt3 and beta-Catenin. Only components that were significantly differentially expressed (P < 0.05) at one or more time points are shown. The analysis is based on head regeneration transcriptome data published in Petersen et al. [34]. Transcript IDs were obtained by BLAST searches of the Hydra head regeneration transcriptome assemblies on https://research.nhgri.nih.gov/hydra (HAS-1: comp9275_c0_seq1; HAS-2: comp23661_c0_seq1; HAS-3: comp16657_c0_seq1; HAS-4: comp9917_c0_seq1; HAS-5: comp19153_c0_seq1; HAS-6: comp21556_c0_seq1; HAS-7: comp23282_c0_seq1; HAS-8: comp23009_c0_seq1; HAS-9: comp16628_c0_seq1; HAS-10: comp22419_c0_seq1; HAS-11: comp8104_c0_seq1; HMP1: comp18584_c0_seq1; HyWnt3: comp17763_c0_seq; beta-Catenin: comp12447_c0_seq1). [file 12915_2021_1046_MOESM9_ESM.png]

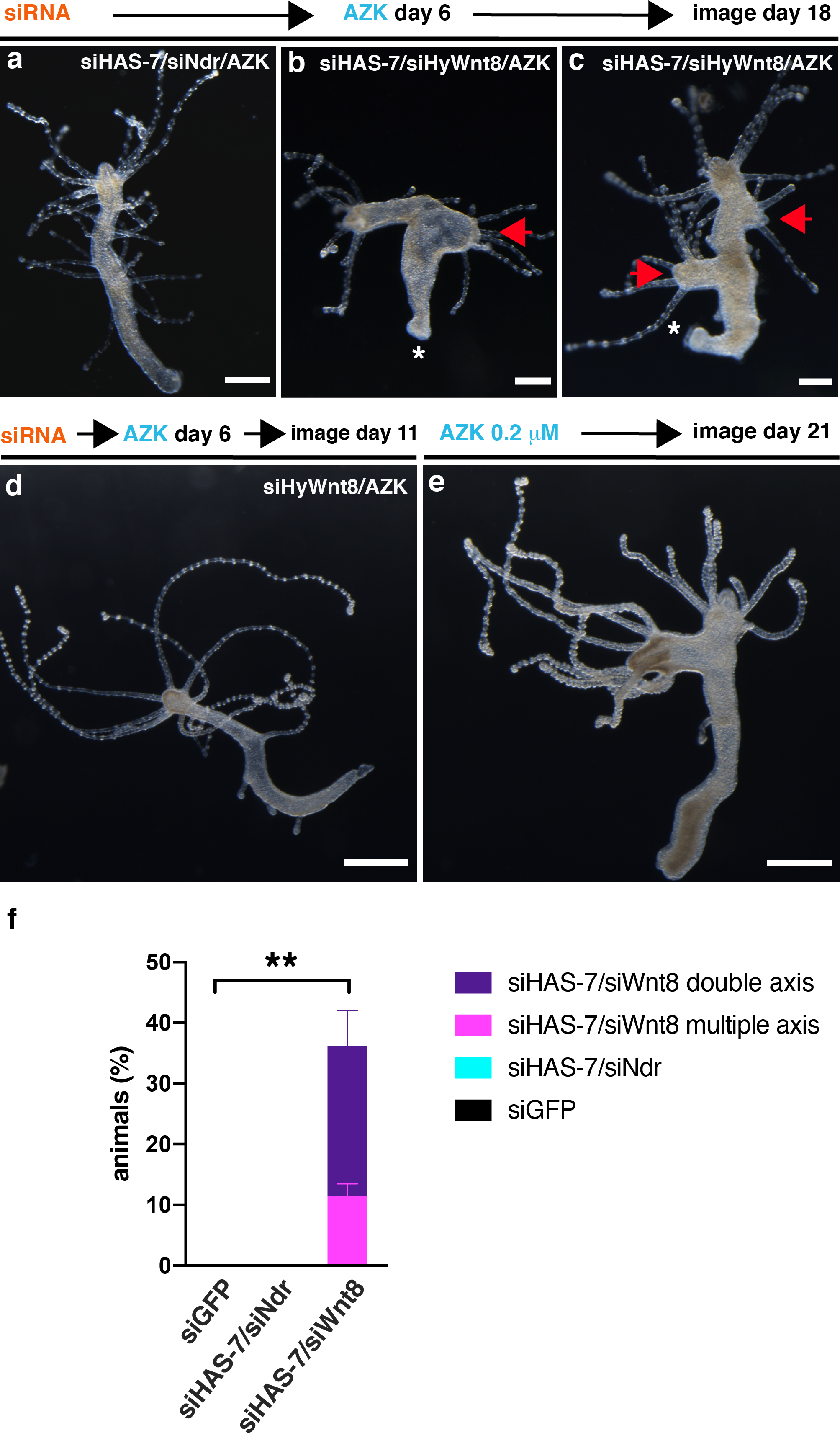

Supplement: Supplementary file 10 — Additional file 10: Fig. S8. Developmental balance between ectopic structures. (a) siHAS-7/siNdr electroporation blocks ectopic axis formation after subsequent AZK treatment. (b-c) siHAS-7/Wnt8 electroporation and AZK treatment reduces ectopic tentacle development in double axis animals (b) and leads to multiple secondary axis formation in a fraction of the treated animals (c). Red arrows denote secondary axes. The asterisk denotes the peduncle region. (d) Ectopic tentacle inhibition is clearly evident in animals electroporated with siWnt8 followed by AZK treatment. Note that few residual ectopic tentacles are detectable in c and d mostly on the side not directly hit by the electroporation pulse. (e) Continuous treatment with a low concentration of ALP (0.2 μM) leads to secondary axis development after about 3 weeks. Note that as in siHAS-7/Wnt8 treated animals, few residual ectopic tentacles are present. Representatives of at least 7 hydras examined. Scale bars = 200 μm. (f) Ratios of double and multiple axis phenotypes in hydras after electroporation with siGFP or combinations of siRNAs as indicated. AZK incubation was started 6 days after electroporation and the numbers of ectopic axes in each group were counted 12 days after AZK removal. Total numbers of animals with ectopic axis phenotype in each group were: siGFP/AZK = 0/38 (n=3), siHAS-7/siNdr/AZK = 0/63 (n=3), siHAS-7/siWnt8/AZK = 22/61 (multiple axis = 7) (n=3), Results from at least three independent experiments are shown. Each column represents the total percentage of one group, bars indicate the mean ± S.E.M. **P value < 0.05. ns = not significant. The data were analyzed using an unpaired T-tests with Welch’s correction. The individual data values are shown in Additional file 14. [file 12915_2021_1046_MOESM10_ESM.png]
