# Supplementary material for: The Wnt-specific astacin proteinase HAS-7 restricts head organizer formation in Hydra
Source: BMC Biol. 2021 Jun 9;19:120. doi: 10.1186/s12915-021-01046-9 (PMC8191133; doi:10.1186/s12915-021-01046-9)
Supplement: Supplementary file 2 — Additional file 2: Table S1. (a) Secretome of Hydra HL HyWnt3 (+) fraction. (b) Secretome of Hydra HL HyWnt3 (-) fraction. [file 12915_2021_1046_MOESM2_ESM.docx]

**Table S1a.** Secretome of *Hydra* HL HyWnt3(+) fraction.

| **No.** | **Accession No.** | **Protein description** | **Protein**  **Score** | **Peptide**  **matches** | **Astacin**  **Protease** | **Other**  **Protease** |
| --- | --- | --- | --- | --- | --- | --- |
| 1 | gi\|828197727 | Fibronectin type III domain-containing protein-like | 1454 | 54 |  |  |
| 2 | gi\|828194560 | Contactin-associated protein-like 2 | 967 | 26 |  |  |
| 3 | gi\|526117389 | Peroxidase PPOD1-like precursor | 817 | 21 |  |  |
| 4 | gi\|449667373 | Peptidyl-prolyl cis-trans isomerase B-like | 793 | 42 |  |  |
| 5 | gi\|221114999 | Chitinase-3-like protein 1 | 792 | 20 |  |  |
| 6 | gi\|828217704 | Collagen alpha-6(VI) chain-like | 788 | 16 |  |  |
| 7 | gi\|828232304 | Blastula protease 10-like / Hydra Astacin 1 (HAS-1) | 668 | 18 | **✄** |  |
| 8 | gi\|15072473 | Peroxidase ppod2 | 575 | 26 |  |  |
| 9 | gi\|221113405 | Chymotrypsin-like elastase family member 3B | 573 | 29 |  | **✄** |
| 10 | gi\|449682831 | Chitinase-3-like protein 1 | 509 | 15 |  |  |
| 11 | gi\|526117401 | PPOD2 peroxidase-like precursor | 475 | 13 |  |  |
| 12 | gi\|828219566 | Chitotriosidase-1-like | 472 | 11 |  |  |
| 13 | gi\|828215752 | Zinc metalloproteinase nas-4-like / Hydra Astacin 7 (HAS-7) | 459 | 17 | **✄** |  |
| 14 | gi\|221130733 | Astacin-like metalloprotease toxin 5 / Hydra Astacin 2 (HAS-2) | 415 | 14 | **✄** |  |
| 15 | gi\|828204323 | Hemicentin-2-like isoform X1 | 408 | 12 |  |  |
| 16 | gi\|221121571 | Zinc metalloproteinase nas-15-like / Hydra Astacin 9 (HAS-9) | 372 | 14 | **✄** |  |
| 17 | gi\|221129013 | Protein PRY1-like | 341 | 12 |  |  |
| 18 | gi\|221119142 | Zinc carboxypeptidase-like | 333 | 11 |  | **✄** |
| 19 | gi\|830260228 | Matrix metalloproteinase-14-like precursor | 318 | 8 |  | **✄** |
| 20 | gi\|828234415 | Carbonic anhydrase 7-like | 313 | 8 |  |  |
| 21 | gi\|830260307 | HMP-1 | 311 | 12 | **✄** |  |
| 22 | gi\|449671849 | Protein disulfide-isomerase A3-like | 305 | 7 |  |  |
| 23 | gi\|828218801 | Zinc metalloproteinase nas-13-like / Hydra Astacin 11 (HAS-11) | 278 | 11 | **✄** |  |
| 24 | gi\|828208094 | Protein DD3-3-like | 254 | 5 |  |  |
| 25 | gi\|828191663 | Neogenin-like | 249 | 5 |  |  |
| 26 | gi\|828194030 | Uncharacterized protein LOC100200589 | 249 | 5 |  |  |
| 27 | gi\|828195809 | Astacin-like metalloprotease toxin 5 / Hydra Astacin 3 (HAS-3) | 213 | 5 | **✄** |  |
| 28 | gi\|221126057 | Antistasin-like | 212 | 12 |  |  |
| 29 | gi\|828203414 | Protein DD3-3-like | 210 | 5 |  |  |
| 30 | gi\|221113429 | Zinc metalloproteinase nas-4-like / Hydra Astacin 8 (HAS-8) | 199 | 7 | **✄** |  |
| 31 | gi\|221111801 | Uncharacterized protein LOC100215485 | 194 | 4 |  |  |
| 33 | gi\|526117507 | Kazal-type serine protease inihibitor 2 precursor | 181 | 6 |  |  |
| 34 | gi\|449690619 | Low choriolytic enzyme-like / Hydra Astacin 10 (HAS-10) | 173 | 4 | **✄** |  |
| 35 | gi\|828226352 | Glutathione peroxidase-like | 169 | 3 |  |  |
| 36 | gi\|221130731 | Protein Span-like / Hydra Astacin 4 (HAS-4) | 161 | 3 | **✄** |  |
| 37 | gi\|449666332 | Zinc metalloproteinase nas-6-like / Hydra Astacin 6 (HAS-6) | 160 | 6 | **✄** |  |
| 38 | gi\|221124062 | Heme-binding protein 1-like | 145 | 5 |  |  |
| 39 | gi\|449677685 | Ferritin heavy chain-like | 143 | 6 |  |  |
| 40 | gi\|221132488 | Uncharacterized protein LOC100213474 | 140 | 3 |  |  |
| 41 | gi\|828215949 | Alpha-L-fucosidase-like isoform X1 | 135 | 3 |  |  |
| 42 | gi\|449664802 | Epididymal secretory protein E1-like | 133 | 2 |  |  |
| 43 | gi\|221130772 | Carboxypeptidase B-like | 121 | 3 |  | **✄** |
| 44 | gi\|828206799 | Probable G-protein coupled receptor 112 | 113 | 5 |  |  |
| 45 | gi\|828224549 | Uncharacterized protein LOC100205745 | 111 | 3 |  |  |
| 46 | gi\|449686976 | Protein disulfide-isomerase A6-like | 108 | 3 |  |  |
| 47 | gi\|221125481 | Protein Span-like / Hydra Astacin 5 (HAS-5) | 94 | 3 | **✄** |  |
| 48 | gi\|828227729 | Protein DD3-3-like, partial | 86 | 2 |  |  |
| 49 | gi\|828225443 | Thrombospondin type-1 domain-containing protein 7A-like | 82 | 2 |  |  |
| 50 | gi\|828196752 | Probable G-protein coupled receptor 112 isoform X1 | 81 | 2 |  |  |
| 51 | gi\|526117631 | Cysteine-rich BMP regulator 2 precursor | 79 | 2 |  |  |

Unique protein hits resulting from the Orbitrap mass spectrometry analysis are listed in descending order according to their MASCOT protein score. The table comprises proteins selected for having a leader peptide. The complete list of protein hits for the HL HyWnt3(+) is given in Table S2.

**Table S1b.** Secretome of *Hydra* HL HyWnt3(-) fraction.

| **No.** | **Accession No.** | **Protein description** | **Protein**  **Score** | **Peptide**  **matches** | **Astacin**  **Protease** | **Other**  **Protease** |
| --- | --- | --- | --- | --- | --- | --- |
| 1 | gi\|828198642 | Uncharacterized protein LOC100198704, partial | 1262 | 32 |  |  |
| 2 | gi\|449671849 | Protein disulfide-isomerase A3-like | 1196 | 37 |  |  |
| 3 | gi\|828197727 | Fibronectin type III domain-containing protein-like | 777 | 28 |  |  |
| 4 | gi\|828201587 | Probable protein disulfide-isomerase A6 | 740 | 23 |  |  |
| 5 | gi\|449667373 | Peptidyl-prolyl cis-trans isomerase B-like | 498 | 16 |  |  |
| 6 | gi\|221132017 | 78 kda glucose-regulated protein-like | 409 | 10 |  |  |
| 8 | gi\|146271914 | Thrombospondin type 1 repeat-containing protein 2 precursor | 402 | 11 |  |  |
| 9 | gi\|221113405 | Chymotrypsin-like elastase family member 3B | 395 | 13 |  | **✄** |
| 10 | gi\|449667073 | Acidic mammalian chitinase-like | 390 | 12 |  |  |
| 11 | gi\|526117559 | Four-domain proteases inhibitor-like precursor | 386 | 11 |  |  |
| 12 | gi\|828217704 | Collagen alpha-6(VI) chain-like | 368 | 7 |  |  |
| 13 | gi\|828220687 | Protein disulfide-isomerase A4-like | 365 | 7 |  |  |
| 14 | gi\|449686976 | Protein disulfide-isomerase A6-like | 354 | 7 |  |  |
| 15 | gi\|828202697 | Peroxiredoxin-4-like | 339 | 10 |  |  |
| 16 | gi\|449690552 | Chymotrypsin-like elastase family member 3B | 325 | 11 |  | **✄** |
| 17 | gi\|221114999 | Chitinase-3-like protein 1 | 313 | 8 |  |  |
| 18 | gi\|221121571 | Zinc metalloproteinase nas-15-like / Hydra Astacin 9 (HAS-9) | 312 | 7 | **✄** |  |
| 19 | gi\|828199374 | Probable protein disulfide-isomerase A4 | 310 | 7 |  |  |
| 20 | gi\|828234415 | Carbonic anhydrase 7-like | 299 | 9 |  |  |
| 21 | gi\|221132488 | Uncharacterized protein LOC100213474 | 297 | 8 |  |  |
| 22 | gi\|828194030 | Uncharacterized protein LOC100200589 | 294 | 7 |  |  |
| 23 | gi\|221118599 | Dolichyl-diphosphooligosaccharide--protein glycosyltransferase subunit 1-like | 293 | 6 |  |  |
| 24 | gi\|828225443 | Thrombospondin type-1 domain-containing protein 7A-like | 291 | 7 |  |  |
| 25 | gi\|449685905 | Acid ceramidase-like | 277 | 8 |  |  |
| 26 | gi\|221119142 | Zinc carboxypeptidase-like | 275 | 8 |  | **✄** |
| 27 | gi\|828218618 | Putative phospholipase B-like 2 | 256 | 4 |  |  |
| 28 | gi\|221129013 | Protein PRY1-like | 249 | 5 |  |  |
| 29 | gi\|828224104 | Beta-glucuronidase-like | 218 | 6 |  |  |
| 30 | gi\|449666857 | Lysosomal aspartic protease-like | 212 | 6 |  | **✄** |
| 31 | gi\|828215949 | Alpha-L-fucosidase-like isoform X1 | 201 | 5 |  |  |
| 32 | gi\|828213548 | Uncharacterized protein LOC105845774 | 193 | 4 |  |  |
| 33 | gi\|828197619 | Lysosomal alpha-mannosidase-like | 175 | 4 |  |  |
| 34 | gi\|449684402 | Endochitinase 1-like isoform X1 | 162 | 3 |  |  |
| 35 | gi\|449678353 | Bandaporin-like (pore forming toxin) | 161 | 4 |  |  |
| 36 | gi\|221126057 | Antistasin-like | 154 | 11 |  |  |
| 37 | gi\|828191663 | Neogenin-like (receptor) | 145 | 4 |  |  |
| 38 | gi\|221090861 | Cathepsin L1-like | 141 | 3 |  | **✄** |
| 39 | gi\|449666332 | Zinc metalloproteinase nas-6-like / Hydra Astacin 6 (HAS-6) | 136 | 4 | **✄** |  |
| 40 | gi\|526117401 | PPOD2 peroxidase-like precursor | 135 | 2 |  |  |
| 41 | gi\|449667021 | Zinc metalloproteinase nas-14-like | 135 | 3 | **✄** |  |
| 42 | gi\|221124062 | Heme-binding protein 1-like | 134 | 5 |  |  |
| 43 | gi\|526117507 | Kazal-type serine protease inihibitor 2 precursor | 134 | 5 |  |  |
| 44 | gi\|828232304 | Blastula protease 10-like / Hydra Astacin 1 (HAS-1) | 129 | 3 | **✄** |  |
| 45 | gi\|15072473 | Peroxidase ppod2 | 119 | 3 |  |  |
| 46 | gi\|449670322 | Dipeptidyl peptidase 1-like | 112 | 3 |  | **✄** |
| 47 | gi\|221130733 | Astacin-like metalloprotease toxin 5 / Hydra Astacin 2 (HAS-2) | 108 | 2 | **✄** |  |
| 48 | gi\|449670247 | Probable inactive purple acid phosphatase 2 | 104 | 2 |  |  |
| 49 | gi\|449665331 | Contactin-associated protein-like 5 | 102 | 3 |  |  |
| 50 | gi\|526117489 | Kazal-type serine protease inihibitor 3 precursor | 102 | 5 |  |  |
| 51 | gi\|828196768 | Calsequestrin-2-like | 101 | 3 |  |  |
| 52 | gi\|449680876 | Endochitinase 4-like | 98 | 2 |  |  |
| 53 | gi\|221121832 | Multiple inositol polyphosphate phosphatase 1-like | 96 | 2 |  |  |
| 54 | gi\|449687197 | Golgi-associated plant pathogenesis-related protein 1-like | 92 | 3 |  |  |
| 55 | gi\|828204323 | Hemicentin-2-like isoform X1 | 92 | 2 |  |  |
| 56 | gi\|449682262 | Zinc metalloproteinase nas-13-like | 87 | 3 | **✄** |  |
| 57 | gi\|221113277 | Uncharacterized protein LOC100214198 | 86 | 4 |  |  |
| 58 | gi\|828212124 | MAM and LDL-receptor class A domain-containing protein 1-like | 79 | 2 |  |  |
| 59 | gi\|449679397 | Uncharacterized protein LOC100197967 | 75 | 3 |  |  |
| 60 | gi\|221124690 | Endoplasmin-like | 71 | 2 |  |  |
| 61 | gi\|828195809 | Astacin-like metalloprotease toxin 5 / Hydra Astacin 3 (HAS-3) | 67 | 2 | **✄** |  |
| 62 | gi\|828231348 | Transmembrane 9 superfamily member 2-like | 67 | 2 |  |  |

Unique protein hits resulting from the Orbitrap mass spectrometry analysis are listed in descending order according to their MASCOT protein score. The table comprises proteins selected for having a leader peptide. The complete list of protein hits for the HL HyWnt3(-) is given in Table S2.
