# Supplementary material for: The Wnt-specific astacin proteinase HAS-7 restricts head organizer formation in Hydra
Source: BMC Biol. 2021 Jun 9;19:120. doi: 10.1186/s12915-021-01046-9 (PMC8191133; doi:10.1186/s12915-021-01046-9)
Supplement: Supplementary file 3 — Additional file 3: Table S2. Complete proteome data of HyWnt3(+) and HyWnt3(-) HL fractions. [file 12915_2021_1046_MOESM3_ESM.pdf]

Table S2. Complete proteome data of HyWnt3(+) and HyWnt3(-) HL fractions.

HyWnt3(+) Fraction

Hit No.	Accession No.	Protein Description	Score	Mass [Da]	Prot Matches	sign Prot Matches	Prot Sequences	sign Prot Sequences	Coverage [%]
1	gi|47132620	keratin, type II cytoskeletal 2 epidermal [Homo sapiens]	2873	65678	117	39	41	21	75,6
2	gi|11935049	keratin 1 [Homo sapiens]	2763	66198	174	51	45	19	64,1
3	gi|21961605	Keratin 10 [Homo sapiens]	2268	59020	116	32	32	15	51,9
4	gi|795247621	PREDICTED: keratin, type I cytoskeletal 16 isoform X1 [Mandrillus leucophaeu:	2232	92585	88	17	41	11	49,1
5	gi|332206121	PREDICTED: keratin, type II cytoskeletal 2 epidermal isoform X1 [Nomascus leu	2108	66822	92	30	31	15	42,2
6	gi|795536398	PREDICTED: keratin, type II cytoskeletal 1 [Cercocebus atys]	2040	65879	141	42	35	15	47,2
7	gi|435476	cytokeratin 9 [Homo sapiens]	1984	62320	95	22	31	12	60,4
8	gi|908801	keratin type II [Homo sapiens]	1907	60448	74	17	35	10	53,2
9	gi|5031839	keratin, type II cytoskeletal 6C [Homo sapiens]	1905	60293	75	17	35	10	53,2
10	gi|1195531	type I keratin 16 [Homo sapiens]	1882	51548	76	15	33	9	68,5
11	gi|155969697	keratin, type II cytoskeletal 6C [Homo sapiens]	1877	60273	74	17	35	10	53,2
12	gi|635065484	PREDICTED: keratin, type II cytoskeletal 6A [Chlorocebus sabaeus]	1848	60236	74	16	34	9	48,4
13	gi|795360291	PREDICTED: keratin, type II cytoskeletal 6A [Colobus angolensis palliatus]	1812	60225	79	20	33	10	46,8
14	gi|908803	keratin type II [Homo sapiens]	1805	60472	72	16	34	9	53,2
15	gi|395744310	PREDICTED: keratin, type II cytoskeletal 6B [Pongo abelii]	1685	60398	72	16	31	9	45,7
16	gi|18999435	Keratin 5 [Homo sapiens]	1572	62568	62	16	31	10	41,7
17	gi|828197727	PREDICTED: fibronectin type III domain-containing protein-like [Hydra vulgari	1454	152931	54	9	26	9	25,3
18	gi|803121007	PREDICTED: keratin, type I cytoskeletal 14 isoform X1 [Ovis aries]	1287	49277	54	9	23	6	48,5
19	gi|676264984	Keratin, type II cytoskeletal 1 [Fukomys damarensis]	1202	159046	63	21	20	8	11,4
20	gi|821004446	PREDICTED: keratin, type II cytoskeletal 1 [Nomascus leucogenys]	1149	32860	81	26	20	7	48,8
21	gi|927195534	PREDICTED: keratin, type I cytoskeletal 17 isoform X2 [Sus scrofa]	1146	50320	43	9	20	6	38,4
22	gi|4557701	keratin, type I cytoskeletal 17 [Homo sapiens]	1110	48361	39	7	20	6	41,2
23	gi|828194560	PREDICTED: contactin-associated protein-like 2 [Hydra vulgaris]	967	138908	26	4	20	4	21,7
24	gi|558156247	PREDICTED: keratin, type II cytoskeletal 1 [Myotis lucifugus]	933	63621	60	20	14	7	17,6
25	gi|221108525	PREDICTED: malate dehydrogenase, mitochondrial-like [Hydra vulgaris]	866	36471	23	7	16	4	52,9
26	gi|1351907	RecName: Full=Serum albumin; AltName: Full=BSA; AltName: Allergen=Bos d 6	828	71244	29	7	17	4	32,9
27	gi|526117389	peroxidase PPOD1-like precursor [Hydra vulgaris]	817	32270	21	5	15	4	58,6
28	gi|301775745	PREDICTED: keratin, type II cytoskeletal 2 epidermal isoform X1 [Ailuropoda m	801	65009	35	13	12	6	16
29	gi|449667373	PREDICTED: peptidyl-prolyl cis-trans isomerase B-like [Hydra vulgaris]	793	22428	42	13	12	5	69,2
30	gi|221114999	PREDICTED: chitinase-3-like protein 1 [Hydra vulgaris]	792	52131	20	3	17	3	42,6
31	gi|344257799	Keratin, type II cytoskeletal 1b [Cricetulus griseus]	790	93670	53	12	14	5	12,8
32	gi|828217704	PREDICTED: collagen alpha-6(VI) chain-like [Hydra vulgaris]	788	68564	16	6	13	5	26,7
33	gi|449690552	PREDICTED: chymotrypsin-like elastase family member 3B [Hydra vulgaris]	764	29089	43	6	14	5	66,5
34	gi|828208682	PREDICTED: filamin-A-like [Hydra vulgaris]	698	294642	15	2	15	2	6,8
35	gi|828232304	PREDICTED: blastula protease 10-like [Hydra vulgaris]	668	27019	18	5	10	4	58
36	gi|828203690	PREDICTED: hemicentin-1-like [Hydra vulgaris]	667	85973	15	4	13	4	19,6
37	gi|221122379	PREDICTED: staphylococcal nuclease domain-containing protein 1-like [Hydra	647	100759	19	4	11	4	15,6
38	gi|828229911	PREDICTED: 2-amino-3-ketobutyrate coenzyme A ligase, mitochondrial-like [H	645	46963	17	4	12	3	33,8
39	gi|221102389	PREDICTED: protein PRY1-like [Hydra vulgaris]	599	20363	48	5	12	3	74,9
40	gi|828224124	PREDICTED: catalase-like [Hydra vulgaris]	578	57689	18	1	14	1	33,3
41	gi|15072473	peroxidase ppod2 [Hydra vulgaris]	575	33192	26	1	12	1	38,3
42	gi|221113405	PREDICTED: chymotrypsin-like elastase family member 3B [Hydra vulgaris]	573	29175	29	4	10	3	52,1
43	gi|829991204	PREDICTED: desmoplakin isoform X1 [Microcebus murinus]	565	335112	14	2	14	2	5,7
44	gi|828218611	PREDICTED: nucleoside diphosphate kinase A-like [Hydra vulgaris]	520	25885	22	2	11	2	53,2
45	gi|449682831	PREDICTED: chitinase-3-like protein 1 [Hydra vulgaris]	509	52203	15	1	11	1	29,8

46	gi|198139671	fructose 1,6-bisphosphate aldolase [Artemia franciscana]	506	25865	12	7	8	6	43,5
47	gi|697450140	Keratin, type II cytoskeletal 75 [Charadrius vociferus]	502	63147	35	12	9	2	12,5
48	gi|526117401	PPOD2 peroxidase-like precursor [Hydra vulgaris]	475	32661	13	1	11	1	38,4
49	gi|449669348	PREDICTED: gamma-glutamyltranspeptidase 1-like [Hydra vulgaris]	475	63187	17	1	12	1	23,6
50	gi|828219566	PREDICTED: chitotriosidase-1-like [Hydra vulgaris]	472	27976	11	2	9	2	38,9
51	gi|221115109	PREDICTED: profilin-like [Hydra vulgaris]	467	14833	20	6	8	3	75,5
52	gi|221130743	PREDICTED: uncharacterized protein LOC100211495 [Hydra vulgaris]	461	13958	24	6	7	3	52,4
53	gi|828215752	PREDICTED: zinc metalloproteinase nas-4-like [Hydra vulgaris]	459	38857	17	1	10	1	33,1
54	gi|260223405	hypothetical protein Csp_B21540 [Curvibacter putative symbiont of Hydra ma	435	35777	7	5	7	5	33,2
55	gi|31074631	keratin 1b [Homo sapiens]	430	62049	27	8	8	2	12,6
56	gi|449662449	PREDICTED: polyubiquitin-B [Hydra vulgaris]	424	42564	13	2	8	2	20,8
57	gi|221130733	PREDICTED: astacin-like metalloprotease toxin 5 [Hydra vulgaris]	415	27816	14	4	6	3	33,6
58	gi|828204323	PREDICTED: hemicentin-2-like isoform X1 [Hydra vulgaris]	408	158275	12	1	9	1	7,9
59	gi|3318722	Chain E, Leech-Derived Tryptase InhibitorTRYPSIN COMPLEX	399	24142	37	3	6	1	26
60	gi|194373749	unnamed protein product [Homo sapiens]	390	62862	14	1	8	1	14,2
61	gi|221121571	PREDICTED: zinc metalloproteinase nas-15-like [Hydra vulgaris]	372	40888	14	0	9	0	28,5
62	gi|829966121	PREDICTED: LOW QUALITY PROTEIN: polyubiquitin-B [Microcebus murinus]	359	25839	10	0	8	0	33,6
63	gi|646697674	Fructose-bisphosphate aldolase [Zootermopsis nevadensis]	343	39761	8	5	6	4	15,4
64	gi|221129013	PREDICTED: protein PRY1-like [Hydra vulgaris]	341	18775	12	2	7	2	56,2
65	gi|565324183	nucleoside diphosphate kinase-like [Hydra vulgaris]	340	17048	12	1	7	1	58,9
66	gi|449667021	PREDICTED: zinc metalloproteinase nas-14-like [Hydra vulgaris]	335	20331	12	1	7	1	42,3
67	gi|221119142	PREDICTED: zinc carboxypeptidase-like [Hydra vulgaris]	333	47718	11	1	9	1	29
68	gi|830260228	matrix metalloproteinase-14-like precursor [Hydra vulgaris]	318	55390	8	2	8	2	16,1
69	gi|449661907	PREDICTED: low choriolytic enzyme-like [Hydra vulgaris]	317	33953	12	2	6	1	22,3
70	gi|828195674	PREDICTED: gamma-glutamyltranspeptidase 1-like [Hydra vulgaris]	316	67827	7	1	7	1	14,3
71	gi|828234415	PREDICTED: carbonic anhydrase 7-like [Hydra vulgaris]	313	36918	8	0	8	0	29,1
72	gi|830260307	zinc metalloproteinase nas-15-like precursor [Hydra vulgaris]	311	33203	12	1	6	1	30,5
73	gi|449671849	PREDICTED: protein disulfide-isomerase A3-like [Hydra vulgaris]	305	55425	7	0	7	0	15,7
74	gi|242025190	Fructose-bisphosphate aldolase, putative [Pediculus humanus corporis]	298	40383	6	4	5	3	11,5
75	gi|725586596	PREDICTED: polyubiquitin-like [Saimiri boliviensis boliviensis]	297	18868	8	0	6	0	41,2
76	gi|828209655	PREDICTED: thymocyte nuclear protein 1-like [Hydra vulgaris]	278	29264	9	0	8	0	30,8
77	gi|449682262	PREDICTED: zinc metalloproteinase nas-13-like [Hydra vulgaris]	278	58069	11	1	7	1	18,3
78	gi|159163108	Chain A, Solution Structure Of The Designed Hydrophobic Core Mutant Of Ubi	277	8580	8	0	6	0	64,5
79	gi|449678439	PREDICTED: peptidyl-prolyl cis-trans isomerase 5-like [Hydra vulgaris]	266	25229	9	1	6	1	34,1
80	gi|499801699	membrane protein [Polaromonas sp. JS666]	259	41227	4	3	4	3	15,8
81	gi|221131289	PREDICTED: methylmalonyl-CoA epimerase, mitochondrial-like [Hydra vulgari	259	18258	9	1	5	1	33,9
82	gi|828224553	PREDICTED: delta-1-pyrroline-5-carboxylate dehydrogenase, mitochondrial-lil	258	28510	6	1	6	1	29,2
83	gi|697423976	Keratin, type II cytoskeletal 4, partial [Tinamus guttatus]	256	59243	12	0	5	0	7
84	gi|828208094	PREDICTED: protein DD3-3-like [Hydra vulgaris]	254	69972	5	1	5	1	10,7
85	gi|33304714	ubiquitin/actin fusion protein 2 [Bigelowiella natans]	253	51325	8	0	6	0	14,4
86	gi|828191663	PREDICTED: neogenin-like [Hydra vulgaris]	249	209395	5	1	5	1	3,5
87	gi|828194030	PREDICTED: uncharacterized protein LOC100200589 [Hydra vulgaris]	249	86226	5	0	5	0	8,2
88	gi|676246284	Keratin, type I cytoskeletal 15 [Balearica regulorum gibbericeps]	248	48549	11	0	6	0	10,8
89	gi|828214768	PREDICTED: delta-1-pyrroline-5-carboxylate dehydrogenase, mitochondrial-lil	247	40600	7	1	6	1	20,3
90	gi|449675219	PREDICTED: uncharacterized protein LOC101234497 [Hydra vulgaris]	240	27697	6	1	6	1	28,9
91	gi|33346945	ubiquitin/actin fusion protein [Gymnochlora stellata]	236	49580	7	0	5	0	13,3
92	gi|30506	desmoglein type 1 [Homo sapiens]	230	114670	7	0	6	0	7,3
93	gi|16751921	dermcidin isoform 1 preproprotein [Homo sapiens]	229	11391	8	1	4	1	35,5

94	gi|762885	Plakoglobin [Homo sapiens]	225	82381	5	0	5	0	8,7
95	gi|828195809	PREDICTED: astacin-like metalloprotease toxin 5 [Hydra vulgaris]	213	28949	5	0	5	0	19,8
96	gi|221126057	PREDICTED: antistasin-like [Hydra vulgaris]	212	26072	12	1	5	1	27,4
97	gi|828217561	PREDICTED: zinc metalloproteinase nas-15-like, partial [Hydra vulgaris]	211	31952	4	1	4	1	16,5
98	gi|828203414	PREDICTED: protein DD3-3-like [Hydra vulgaris]	210	76301	5	0	5	0	13
99	gi|28336	mutant beta-actin (beta'-actin) [Homo sapiens]	208	42128	6	0	6	0	18,4
100	gi|830260001	tight junction protein ZO-2-like [Hydra vulgaris]	204	191163	3	2	3	2	2,8
101	gi|221113429	PREDICTED: zinc metalloproteinase nas-4-like [Hydra vulgaris]	199	41939	7	0	5	0	14,2
102	gi|221111801	PREDICTED: uncharacterized protein LOC100215485 [Hydra vulgaris]	194	19588	4	1	3	1	26,7
103	gi|828221194	PREDICTED: fibronectin type III domain-containing protein-like [Hydra vulgari	193	143336	5	0	5	0	5,2
104	gi|828198152	PREDICTED: glycogenin-1-like [Hydra vulgaris]	192	39767	4	1	4	1	14,1
105	gi|740383561	membrane protein [Xenophilus azovorans]	191	41199	3	2	3	2	12,1
106	gi|586632450	Purine-binding protein precursor [Hydrogenophaga sp. T4]	190	31703	3	2	3	2	15,5
107	gi|84402	glutathione transferase (EC 2.5.1.18) - fluke (Schistosoma japonicum) (fragmer	190	25834	5	1	4	1	20,1
108	gi|449689369	PREDICTED: protein NipSnap homolog 1-like [Hydra vulgaris]	188	32344	5	0	5	0	21,5
109	gi|21755908	unnamed protein product [Homo sapiens]	183	57583	4	0	4	0	7,9
110	gi|526117507	kazal-type serine protease inihibitor 2 precursor [Hydra vulgaris]	181	19400	6	0	6	0	33,9
111	gi|828203917	PREDICTED: aminomethyltransferase, mitochondrial-like [Hydra vulgaris]	178	44995	4	0	4	0	11
112	gi|449690619	PREDICTED: low choriolytic enzyme-like [Hydra vulgaris]	173	38696	4	0	4	0	15,2
113	gi|828226352	PREDICTED: glutathione peroxidase-like [Hydra vulgaris]	169	26056	3	1	3	1	20,9
114	gi|828222174	PREDICTED: 3-hydroxyacyl-CoA dehydrogenase type-2-like [Hydra vulgaris]	168	26807	3	1	3	1	13,4
115	gi|828206951	PREDICTED: uncharacterized protein LOC100208668 isoform X1 [Hydra vulga	166	253566	5	0	5	0	2,9
116	gi|221130731	PREDICTED: protein SpAN-like [Hydra vulgaris]	161	35171	3	1	3	1	10,3
117	gi|449666332	PREDICTED: zinc metalloproteinase nas-6-like [Hydra vulgaris]	160	33291	6	0	4	0	19,1
118	gi|221124062	PREDICTED: heme-binding protein 1-like [Hydra vulgaris]	145	29921	5	0	4	0	14,8
119	gi|449677685	PREDICTED: ferritin heavy chain-like [Hydra vulgaris]	143	27387	6	0	5	0	25,9
120	gi|221132488	PREDICTED: uncharacterized protein LOC100213474 [Hydra vulgaris]	140	27971	3	1	3	1	14,7
121	gi|449678564	PREDICTED: lysosome-associated membrane glycoprotein 1-like [Hydra vulgari	137	21879	5	0	3	0	15,7
122	gi|828215949	PREDICTED: alpha-L-fucosidase-like isoform X1 [Hydra vulgaris]	135	56266	3	0	3	0	5,4
123	gi|449664802	PREDICTED: epididymal secretory protein E1-like [Hydra vulgaris]	133	16839	2	1	2	1	18,7
124	gi|828216609	PREDICTED: salivary glue protein Sgs-3-like, partial [Hydra vulgaris]	129	34483	2	1	2	1	11,2
125	gi|221132768	PREDICTED: RNA polymerase II transcriptional coactivator-like [Hydra vulgaris	129	13128	3	0	3	0	27
126	gi|395826450	PREDICTED: keratin, type I cytoskeletal 28 [Otolemur garnettii]	129	51136	13	0	3	0	5,4
127	gi|449683097	PREDICTED: peptidyl-prolyl cis-trans isomerase-like [Hydra vulgaris]	125	17530	3	0	3	0	24,2
128	gi|828222429	PREDICTED: AP-2 complex subunit alpha-2-like [Hydra vulgaris]	122	103472	4	0	4	0	4,2
129	gi|526117746	flp protein [Hydra vulgaris]	121	14995	4	0	3	0	26,2
130	gi|221130772	PREDICTED: carboxypeptidase B-like [Hydra vulgaris]	121	51584	3	0	3	0	5,8
131	gi|46621276	CEP152 protein, partial [Homo sapiens]	118	68324	2	1	2	1	7,4
132	gi|58005	aprotinin [synthetic construct]	116	7011	4	1	2	1	47,5
133	gi|62122917	filaggrin-2 [Homo sapiens]	114	249296	3	1	2	1	1
134	gi|828206799	PREDICTED: probable G-protein coupled receptor 112 [Hydra vulgaris]	113	98355	5	0	3	0	3,8
135	gi|828224549	PREDICTED: uncharacterized protein LOC100205745 [Hydra vulgaris]	111	70074	3	0	3	0	5,5
136	gi|449686976	PREDICTED: protein disulfide-isomerase A6-like [Hydra vulgaris]	108	48142	3	0	2	0	8,2
137	gi|221121838	PREDICTED: endothelin-converting enzyme 1-like [Hydra vulgaris]	105	88293	3	0	2	0	2,7
138	gi|828209711	PREDICTED: protein DD3-3-like [Hydra vulgaris]	105	62180	2	0	2	0	3,6
139	gi|828235608	PREDICTED: cytosol aminopeptidase-like [Hydra vulgaris]	104	56366	3	0	3	0	7,5
140	gi|828197350	PREDICTED: contactin-2-like [Hydra vulgaris]	100	93069	2	1	2	1	2,2
141	gi|828202743	PREDICTED: uncharacterized protein LOC100202739 isoform X1 [Hydra vulga	98	869663	3	0	3	0	0,4

142	gi|223130	fibrinogen betaB 1-118	97	12891	5	2	1	1	11,9
143	gi|449690428	PREDICTED: nascent polypeptide-associated complex subunit alpha-like [Hydr	96	24795	2	0	2	0	11,8
144	gi|449687420	PREDICTED: uncharacterized protein LOC100205832 isoform X1 [Hydra vulgar	96	24618	2	0	2	0	8,4
145	gi|449674503	PREDICTED: uncharacterized protein LOC101239382 [Hydra vulgaris]	95	18169	2	1	2	1	17,4
146	gi|221125481	PREDICTED: protein SpAN-like [Hydra vulgaris]	94	33407	3	0	2	0	10
147	gi|28557150	hornerin [Homo sapiens]	92	48797	3	0	2	0	8,1
148	gi|828218801	PREDICTED: zinc metalloproteinase nas-13-like [Hydra vulgaris]	91	57813	3	0	2	0	4,8
149	gi|449680259	PREDICTED: glutathione peroxidase 2-like [Hydra vulgaris]	89	23459	5	0	3	0	15,3
150	gi|170582740	cyclophilin-type peptidyl-prolyl cis-trans isomerase-15, Bmcyp-5 [Brugia mala	88	22456	2	0	2	0	10,8
151	gi|493257619	azurin [Achromobacter piechaudii]	87	16082	1	1	1	1	10,7
152	gi|828227729	PREDICTED: protein DD3-3-like, partial [Hydra vulgaris]	86	54925	2	0	2	0	4
153	gi|449668124	PREDICTED: CUGBP Elav-like family member 2 isoform X3 [Hydra vulgaris]	86	55769	1	1	1	1	3,1
154	gi|828200742	PREDICTED: ADP-ribosyl cyclase/cyclic ADP-ribose hydrolase-like [Hydra vulgar	86	35219	1	1	1	1	3,6
155	gi|828225443	PREDICTED: thrombospondin type-1 domain-containing protein 7A-like [Hydr	82	41599	2	0	2	0	6,5
156	gi|113531039	chitinase 2 [Hydractinia echinata]	81	48377	1	1	1	1	2,8
157	gi|828196752	PREDICTED: probable G-protein coupled receptor 112 isoform X1 [Hydra vulga	81	130717	2	0	2	0	1,9
158	gi|4757756	annexin A2 isoform 2 [Homo sapiens]	80	38808	2	0	2	0	6,2
159	gi|828223895	PREDICTED: myoferlin [Hydra vulgaris]	80	231950	2	0	2	0	1,4
160	gi|526117631	cysteine rich BMP regulator 2 precursor [Hydra vulgaris]	79	132361	2	0	2	0	2,1
161	gi|31645	glyceraldehyde-3-phosphate dehydrogenase [Homo sapiens]	79	36202	2	0	2	0	8,7
162	gi|828189807	PREDICTED: cysteine and glycine-rich protein 1-like [Hydra vulgaris]	76	11681	2	0	2	0	10,2
163	gi|736817112	DNA polymerase III subunit beta [[Eubacterium] nodatum]	74	41468	3	0	2	0	2,2
164	gi|847168677	PREDICTED: LOW QUALITY PROTEIN: keratin-3, type I cytoskeletal 51 kDa-like [	73	52538	3	0	2	0	3,2
165	gi|449683115	PREDICTED: astacin-like, partial [Hydra vulgaris]	73	15801	4	0	2	0	11,6
166	gi|221131483	PREDICTED: uncharacterized protein LOC100199100 [Hydra vulgaris]	72	20190	2	0	2	0	10,9
167	gi|449661942	PREDICTED: arginase-1-like [Hydra vulgaris]	71	36923	1	1	1	1	2,7
168	gi|3891470	Chain A, Crystal Structure Of Human Galectin-7 In Complex With Galactosami	70	14992	1	1	1	1	8,1
169	gi|449673266	PREDICTED: alkyl/aryl-sulfatase BDS1-like [Hydra vulgaris]	70	67246	2	0	2	0	4,8
170	gi|302673269	hypothetical protein SCHCODRAFT_238605 [Schizophyllum commune H4-8]	70	151449	3	0	2	0	0,7
171	gi|27806789	transthyretin precursor [Bos taurus]	69	15831	2	0	2	0	15,6
172	gi|780839549	hypothetical protein VR70_05230 [Rhodospirillaceae bacterium BRH_c57]	68	122394	2	1	1	1	1
173	gi|828209114	PREDICTED: uncharacterized protein LOC101235124 isoform X1 [Hydra vulgar	68	54646	1	1	1	1	3,8
174	gi|47227198	unnamed protein product [Tetraodon nigroviridis]	67	29312	2	0	2	0	7,3
175	gi|4204211	actin-binding protein ABP-280, partial [Hydra vulgaris]	64	24207	1	0	1	0	8,2
176	gi|449661938	PREDICTED: single-stranded DNA-binding protein, mitochondrial-like [Hydra v	64	18793	1	0	1	0	8,4

HyWnt3(-) Fraction

Hit No.	Accession No.	Protein Description	Score	Mass [Da]	Prot Matches	sign Prot Matches	Prot Sequences	sign Prot Sequences	Coverage [%]
1	gi|828208682	PREDICTED: filamin-A-like [Hydra vulgaris]	3228	294642	84	19	59	17	25,7
2	gi|11935049	keratin 1 [Homo sapiens]	1555	66198	46	16	26	12	35,7
3	gi|375314779	keratin 1 [Homo sapiens]	1536	66197	44	14	26	12	35,7
4	gi|28317	unnamed protein product [Homo sapiens]	1282	59720	32	8	23	7	42,3
5	gi|828198642	PREDICTED: uncharacterized protein LOC100198704, partial [Hydra vulgaris]	1262	162069	32	7	24	6	19,9
6	gi|435476	cytokeratin 9 [Homo sapiens]	1225	62320	31	9	21	7	34,2
7	gi|449671849	PREDICTED: protein disulfide-isomerase A3-like [Hydra vulgaris]	1196	55425	37	13	20	9	44,3
8	gi|181402	epidermal cytokeratin 2 [Homo sapiens]	1119	66110	27	8	21	7	39,7
9	gi|2392071	Chain A, Crystal Structure Of The Annexin Xii Hexamer	1082	35070	34	7	20	5	57,5
10	gi|828195098	PREDICTED: 60 kDa heat shock protein, mitochondrial-like [Hydra vulgaris]	1036	61302	21	6	19	6	40,2
11	gi|828224124	PREDICTED: catalase-like [Hydra vulgaris]	923	57689	23	5	19	4	37,6
12	gi|221108525	PREDICTED: malate dehydrogenase, mitochondrial-like [Hydra vulgaris]	896	36471	22	5	16	5	51,8
13	gi|828203840	PREDICTED: betaine--homocysteine S-methyltransferase 1-like [Hydra vulgaris]	803	44885	32	8	13	7	28,7
14	gi|449672141	PREDICTED: glutamate dehydrogenase, mitochondrial-like [Hydra vulgaris]	796	59949	21	7	15	6	34,1
15	gi|828197727	PREDICTED: fibronectin type III domain-containing protein-like [Hydra vulgari	777	152931	28	4	16	3	14,7
16	gi|828201587	PREDICTED: probable protein disulfide-isomerase A6 [Hydra vulgaris]	740	27489	23	5	14	4	53,9
17	gi|221115947	PREDICTED: fructose-bisphosphate aldolase A-like [Hydra vulgaris]	721	39494	15	7	11	7	37,1
18	gi|221122379	PREDICTED: staphylococcal nuclease domain-containing protein 1-like [Hydra	693	100759	18	6	13	5	16,3
19	gi|449679909	PREDICTED: transketolase-like protein 2 [Hydra vulgaris]	682	68714	15	4	13	4	31,4
20	gi|828194560	PREDICTED: contactin-associated protein-like 2 [Hydra vulgaris]	668	138908	18	3	15	3	14
21	gi|38640805	cathepsin L-associated protein [Artemia franciscana]	650	34918	19	6	12	4	49,4
22	gi|565324183	nucleoside diphosphate kinase-like [Hydra vulgaris]	642	17048	31	4	13	3	84,1
23	gi|828230018	PREDICTED: alcohol dehydrogenase [NADP(+)]-like [Hydra vulgaris]	630	35493	16	4	11	4	56,5
24	gi|1703135	RecName: Full=Actin, cytoskeletal 3A; AltName: Full=Actin, cytoskeletal IIIA; Fl	551	42162	14	3	12	2	36,2
25	gi|828223684	PREDICTED: transaldolase-like [Hydra vulgaris]	528	36670	13	2	11	2	29,2
26	gi|9739163	keratin 5 [Homo sapiens]	527	62651	16	2	13	2	21,4
27	gi|221115109	PREDICTED: profilin-like [Hydra vulgaris]	520	14833	25	7	8	3	75,5
28	gi|449667373	PREDICTED: peptidyl-prolyl cis-trans isomerase B-like [Hydra vulgaris]	498	22428	16	3	9	3	51,7
29	gi|198139671	fructose 1,6-bisphosphate aldolase [Artemia franciscana]	493	25865	10	6	7	5	29,3
30	gi|449667063	PREDICTED: ADP-ribose pyrophosphatase, mitochondrial-like isoform X2 [Hyd	491	36625	13	3	9	3	34,7
31	gi|828229950	PREDICTED: coadhesin-like, partial [Hydra vulgaris]	487	64136	12	3	10	3	23,3
32	gi|828235386	PREDICTED: serine/threonine-protein phosphatase 6 regulatory ankyrin repea	478	105630	12	2	9	2	10,1
33	gi|1351907	RecName: Full=Serum albumin; AltName: Full=BSA; AltName: Allergen=Bos d 6	466	71244	10	3	9	3	16,3
34	gi|33346945	ubiquitin/actin fusion protein [Gymnochlora stellata]	430	49580	15	2	9	1	23,5
35	gi|828223535	PREDICTED: golgin subfamily B member 1-like [Hydra vulgaris]	424	545146	12	0	12	0	2,5
36	gi|221112786	PREDICTED: Na(+)/H(+) exchange regulatory cofactor NHE-RF1-like [Hydra vulga	414	37612	12	2	7	2	22,1
37	gi|828234048	PREDICTED: lupus La protein homolog A-like [Hydra vulgaris]	414	44610	10	3	7	2	22,9
38	gi|221103278	PREDICTED: fumarate hydratase, mitochondrial-like [Hydra vulgaris]	411	54261	11	2	8	2	20,5
39	gi|221129526	PREDICTED: radixin-like [Hydra vulgaris]	411	66644	15	1	9	1	14,2
40	gi|221132017	PREDICTED: 78 kDa glucose-regulated protein-like [Hydra vulgaris]	409	74120	10	1	8	1	13,1
41	gi|146271914	thrombospondin type 1 repeat-containing protein 2 precursor [Hydra vulgaris	402	102458	11	1	10	1	11,8
42	gi|828210115	PREDICTED: glutathione S-transferase Mu 1-like [Hydra vulgaris]	398	13022	11	3	7	2	57,1
43	gi|221113405	PREDICTED: chymotrypsin-like elastase family member 3B [Hydra vulgaris]	395	29175	13	2	8	2	40,3
44	gi|449667073	PREDICTED: acidic mammalian chitinase-like [Hydra vulgaris]	390	48553	12	1	8	1	25,1
45	gi|828209655	PREDICTED: thymocyte nuclear protein 1-like [Hydra vulgaris]	390	29264	19	1	10	1	37,2

46	gi|828192702	PREDICTED: WD repeat-containing protein 1-A-like isoform X1 [Hydra vulgaris	387	68092	11	1	10	1	17,2
47	gi|526117559	four-domain proteases inhibitor-like precursor [Hydra vulgaris]	386	19425	11	3	8	2	54,2
48	gi|449662397	PREDICTED: fumarylacetoacetase-like [Hydra vulgaris]	385	46438	10	1	9	1	24,5
49	gi|449689369	PREDICTED: protein NipSnap homolog 1-like [Hydra vulgaris]	383	32344	11	0	9	0	33,3
50	gi|4204211	actin-binding protein ABP-280, partial [Hydra vulgaris]	375	24207	17	4	8	1	35,9
51	gi|291406077	PREDICTED: keratin, type I cytoskeletal 13 [Oryctolagus cuniculus]	375	44693	11	0	8	0	16,2
52	gi|449692316	PREDICTED: aldose reductase-like [Hydra vulgaris]	374	13333	11	3	7	2	76,1
53	gi|221091687	PREDICTED: triosephosphate isomerase-like [Hydra vulgaris]	370	27190	8	3	7	3	37,3
54	gi|221130743	PREDICTED: uncharacterized protein LOC100211495 [Hydra vulgaris]	370	13958	13	4	7	3	52,4
55	gi|828217704	PREDICTED: collagen alpha-6(VI) chain-like [Hydra vulgaris]	368	68564	7	2	6	2	12,6
56	gi|2724046	beta-actin [Mustela putorius furo]	367	36099	9	2	8	1	34,1
57	gi|3318722	Chain E, Leech-Derived Tryptase InhibitorTRYPSIN COMPLEX	365	24142	18	2	6	2	26
58	gi|828202745	PREDICTED: uncharacterized protein LOC100202739 isoform X2 [Hydra vulga	365	801426	7	2	7	2	1,2
59	gi|828220687	PREDICTED: protein disulfide-isomerase A4-like [Hydra vulgaris]	365	72268	7	3	7	3	12,9
60	gi|926716364	PREDICTED: keratin, type I cytoskeletal 10 [Capra hircus]	362	52798	10	1	7	1	13,5
61	gi|449692014	PREDICTED: glutathione S-transferase Mu 3-like [Hydra vulgaris]	357	17181	13	3	7	2	53,4
62	gi|449686976	PREDICTED: protein disulfide-isomerase A6-like [Hydra vulgaris]	354	48142	7	2	7	2	19,9
63	gi|221115097	PREDICTED: glutathione S-transferase-like [Hydra vulgaris]	343	24049	13	4	6	2	24,3
64	gi|828202697	PREDICTED: peroxiredoxin-4-like [Hydra vulgaris]	339	27499	10	1	8	1	30,7
65	gi|449662449	PREDICTED: polyubiquitin-B [Hydra vulgaris]	335	42564	15	2	8	2	17,6
66	gi|828199466	PREDICTED: adenylyl cyclase-associated protein-like [Hydra vulgaris]	334	53995	9	3	6	2	16,9
67	gi|828205217	PREDICTED: pirin-like protein [Hydra vulgaris]	333	25924	9	0	8	0	37,9
68	gi|828192654	PREDICTED: myosin heavy chain, embryonic smooth muscle isoform-like [Hyd	330	68182	6	1	6	1	11,3
69	gi|449690552	PREDICTED: chymotrypsin-like elastase family member 3B [Hydra vulgaris]	325	29089	11	2	7	2	31,9
70	gi|697450140	Keratin, type II cytoskeletal 75 [Charadrius vociferus]	325	63147	11	4	6	2	10,2
71	gi|221109840	PREDICTED: glucose-6-phosphate isomerase-like, partial [Hydra vulgaris]	320	15636	7	1	7	1	53,4
72	gi|828218611	PREDICTED: nucleoside diphosphate kinase A-like [Hydra vulgaris]	319	25885	11	1	7	1	39,6
73	gi|828229911	PREDICTED: 2-amino-3-ketobutyrate coenzyme A ligase, mitochondrial-like [H	316	46963	9	2	6	1	16,8
74	gi|221114999	PREDICTED: chitinase-3-like protein 1 [Hydra vulgaris]	313	52131	8	2	7	2	21,3
75	gi|646697674	Fructose-bisphosphate aldolase [Zootermopsis nevadensis]	312	39761	9	4	5	3	12,4
76	gi|221121571	PREDICTED: zinc metalloproteinase nas-15-like [Hydra vulgaris]	312	40888	7	1	6	1	19,6
77	gi|194772468	GF20391 [Drosophila ananassae]	311	21231	8	2	6	1	33,3
78	gi|828199374	PREDICTED: probable protein disulfide-isomerase A4 [Hydra vulgaris]	310	127080	7	1	7	1	7,7
79	gi|386848	keratin [Homo sapiens]	310	51916	11	1	7	1	13,6
80	gi|567757496	guanine nucleotide-binding protein subunit beta-like protein [Hydra vulgaris]	307	35804	9	0	7	0	20,2
81	gi|828234415	PREDICTED: carbonic anhydrase 7-like [Hydra vulgaris]	299	36918	9	1	7	1	22,3
82	gi|221132488	PREDICTED: uncharacterized protein LOC100213474 [Hydra vulgaris]	297	27971	8	1	7	1	34,9
83	gi|828205187	PREDICTED: disks large homolog 1-like [Hydra vulgaris]	297	87452	8	1	8	1	12,9
84	gi|514683975	heat shock protein 60 [Salpingoeca rosetta]	296	61289	6	2	6	2	8
85	gi|828222429	PREDICTED: AP-2 complex subunit alpha-2-like [Hydra vulgaris]	296	103472	7	1	7	1	8,4
86	gi|828194030	PREDICTED: uncharacterized protein LOC100200589 [Hydra vulgaris]	294	86226	7	1	7	1	10,5
87	gi|221118599	PREDICTED: dolichyl-diphosphooligosaccharide--protein glycosyltransferase s	293	68116	6	1	6	1	11,2
88	gi|828225443	PREDICTED: thrombospondin type-1 domain-containing protein 7A-like [Hydr	291	41599	7	1	6	1	18,7
89	gi|828228866	PREDICTED: branched-chain-amino-acid aminotransferase, cytosolic-like [Hyd	288	45736	9	1	8	1	24,1
90	gi|828201601	PREDICTED: probable methylmalonate-semialdehyde dehydrogenase [acylatin	277	77319	6	0	6	0	9,1
91	gi|449685905	PREDICTED: acid ceramidase-like [Hydra vulgaris]	277	42798	8	1	6	1	20,1
92	gi|829966121	PREDICTED: LOW QUALITY PROTEIN: polyubiquitin-B [Microcebus murinus]	275	25839	14	0	7	0	27,5
93	gi|221119142	PREDICTED: zinc carboxypeptidase-like [Hydra vulgaris]	275	47718	8	2	5	2	13,5

94	gi|828213638	PREDICTED: heterogeneous nuclear ribonucleoprotein A0-like [Hydra vulgaris]	272	33657	6	1	6	1	22,1
95	gi|828210482	PREDICTED: branched-chain-amino-acid aminotransferase-like [Hydra vulgaris	272	39139	9	1	7	1	25,4
96	gi|449673266	PREDICTED: alkyl/aryl-sulfatase BDS1-like [Hydra vulgaris]	270	67246	7	0	6	0	15,7
97	gi|449662275	PREDICTED: thioredoxin-like [Hydra vulgaris]	267	11812	9	1	5	1	43,8
98	gi|449676410	PREDICTED: cdc42-interacting protein 4 homolog [Hydra vulgaris]	264	57919	7	1	6	1	13,8
99	gi|828206617	PREDICTED: kinectin-like [Hydra vulgaris]	264	113168	7	0	7	0	7,4
100	gi|565324171	high mobility group-T protein-like [Hydra vulgaris]	261	20500	7	1	6	1	29,9
101	gi|221102389	PREDICTED: protein PRY1-like [Hydra vulgaris]	260	20363	8	1	5	1	32,8
102	gi|221111162	PREDICTED: peroxiredoxin-2-like [Hydra vulgaris]	258	22270	8	1	5	1	20,8
103	gi|221125749	PREDICTED: putative acetyltransferase DDB_G0275913 [Hydra vulgaris]	257	24335	9	1	6	1	33,9
104	gi|828218618	PREDICTED: putative phospholipase B-like 2 [Hydra vulgaris]	256	63709	4	2	4	2	8,9
105	gi|828214833	PREDICTED: major vault protein-like [Hydra vulgaris]	255	96304	7	1	6	1	8,5
106	gi|3046400	actin 1 [Schmidtea polychroa]	251	8257	6	1	5	1	53,8
107	gi|221129013	PREDICTED: protein PRY1-like [Hydra vulgaris]	249	18775	5	1	5	1	32,5
108	gi|828198329	PREDICTED: uncharacterized protein LOC100198634 [Hydra vulgaris]	247	166979	6	1	6	1	4
109	gi|221116483	PREDICTED: translation elongation factor 2-like [Hydra vulgaris]	244	95554	7	0	7	0	8,3
110	gi|221131022	PREDICTED: glucosamine-6-phosphate isomerase 1-like [Hydra vulgaris]	243	30402	4	1	4	1	22,1
111	gi|828234147	PREDICTED: cystatin-A-like [Hydra vulgaris]	242	11384	6	2	4	2	52,5
112	gi|544430751	PREDICTED: uncharacterized protein LOC102118097 [Macaca fascicularis]	238	61529	7	0	6	0	10,4
113	gi|449678439	PREDICTED: peptidyl-prolyl cis-trans isomerase 5-like [Hydra vulgaris]	235	25229	5	2	5	2	24,9
114	gi|315439538	vitellogenin-superoxide dismutase fusion protein [Artemia parthenogenetica]	235	249350	6	0	6	0	3,4
115	gi|830260001	tight junction protein ZO-2-like [Hydra vulgaris]	232	191163	6	2	4	1	2,9
116	gi|1330252	translation elongation factor 1 alpha [Hydra vulgaris]	231	51200	5	0	5	0	10,5
117	gi|449669260	PREDICTED: eukaryotic translation initiation factor 3 subunit B-like [Hydra vul	231	80964	6	1	4	1	6,9
118	gi|449670492	PREDICTED: elongation factor 1-gamma-like [Hydra vulgaris]	228	49912	6	0	5	0	11,5
119	gi|828201918	PREDICTED: glyceraldehyde-3-phosphate dehydrogenase [Hydra vulgaris]	226	36793	8	1	5	1	18,2
120	gi|449665286	PREDICTED: nucleoredoxin-like protein 2 [Hydra vulgaris]	224	16201	4	1	4	1	35,9
121	gi|828230255	PREDICTED: glucose-6-phosphate isomerase-like, partial [Hydra vulgaris]	220	39603	5	0	5	0	22,6
122	gi|828224104	PREDICTED: beta-glucuronidase-like [Hydra vulgaris]	218	73149	6	0	6	0	11
123	gi|828213796	PREDICTED: probable aminopeptidase NPEPL1 [Hydra vulgaris]	217	54416	5	0	5	0	11,2
124	gi|449666857	PREDICTED: lysosomal aspartic protease-like [Hydra vulgaris]	212	42777	6	1	4	1	12,7
125	gi|828218104	PREDICTED: myosin-10-like [Hydra vulgaris]	202	225409	5	0	5	0	2,7
126	gi|221121754	PREDICTED: very long-chain specific acyl-CoA dehydrogenase, mitochondrial-l	202	68420	6	1	6	1	10,2
127	gi|828215949	PREDICTED: alpha-L-fucosidase-like isoform X1 [Hydra vulgaris]	201	56266	5	1	5	1	9,4
128	gi|828228888	PREDICTED: stress-induced-phosphoprotein 1-like [Hydra vulgaris]	197	61796	4	0	4	0	8,2
129	gi|828189807	PREDICTED: cysteine and glycine-rich protein 1-like [Hydra vulgaris]	196	11681	5	1	3	1	25,9
130	gi|449669354	PREDICTED: oxygen-dependent coproporphyrinogen-III oxidase-like [Hydra vu	194	39728	5	1	5	1	14,4
131	gi|828213548	PREDICTED: uncharacterized protein LOC105845774 [Hydra vulgaris]	193	19785	4	0	4	0	27,7
132	gi|221125639	PREDICTED: alanine--glyoxylate aminotransferase 2, mitochondrial-like [Hydra	192	55680	6	0	6	0	12,6
133	gi|221132111	PREDICTED: hypoxia up-regulated protein 1-like [Hydra vulgaris]	191	112944	4	0	4	0	4,2
134	gi|449684469	PREDICTED: stress-70 protein, mitochondrial-like [Hydra vulgaris]	190	72978	6	1	4	1	7,5
135	gi|449669580	PREDICTED: peptidyl-prolyl cis-trans isomerase FKBP9-like [Hydra vulgaris]	189	30984	7	0	6	0	23,1
136	gi|828221194	PREDICTED: fibronectin type III domain-containing protein-like [Hydra vulgari	188	143336	4	1	4	1	4
137	gi|74483475	elongation factor 1 alpha [Ceratinia neso]	187	45127	4	0	4	0	9,7
138	gi|221114449	PREDICTED: nuclear polyadenylated RNA-binding protein 4-like [Hydra vulgari	185	43864	6	1	4	1	10,3
139	gi|221111160	PREDICTED: peroxiredoxin-1-like [Hydra vulgaris]	184	26936	7	1	4	1	15,5
140	gi|828220681	PREDICTED: macrophage migration inhibitory factor-like [Hydra vulgaris]	182	13896	4	2	3	2	18,7
141	gi|321460290	cytosolic malate dehydrogenase [Daphnia pulex]	181	35988	4	0	4	0	15,3

142	gi|209156284	Heat shock 70 kDa protein [Salmo salar]	175	71189	6	0	5	0	6,5
143	gi|828197619	PREDICTED: lysosomal alpha-mannosidase-like [Hydra vulgaris]	175	49198	4	0	4	0	10,2
144	gi|449678000	PREDICTED: S-methyl-5'-thioadenosine phosphorylase-like [Hydra vulgaris]	173	30317	2	2	2	2	11,4
145	gi|221132295	PREDICTED: glutathione S-transferase-like [Hydra vulgaris]	166	23892	6	0	4	0	12
146	gi|828192764	PREDICTED: aminopeptidase N-like [Hydra vulgaris]	166	104641	4	0	4	0	4,4
147	gi|148229965	heat shock 70kDa protein 2 [Xenopus laevis]	166	69803	4	0	4	0	5,2
148	gi|221126616	PREDICTED: THO complex subunit 4-like [Hydra vulgaris]	166	26671	7	0	5	0	17,6
149	gi|754342395	heat shock protein 70 [Capsaspora owczarzaki ATCC 30864]	164	72508	6	0	5	0	6,9
150	gi|291234001	PREDICTED: late histone H2B.L3-like isoform X1 [Saccoglossus kowalevskii]	162	13838	4	2	3	1	28
151	gi|449684402	PREDICTED: endochitinase 1-like isoform X1 [Hydra vulgaris]	162	52676	3	1	3	1	8,5
152	gi|449678353	PREDICTED: bandaporin-like [Hydra vulgaris]	161	22016	4	1	3	1	19,2
153	gi|449683097	PREDICTED: peptidyl-prolyl cis-trans isomerase-like [Hydra vulgaris]	158	17530	3	0	3	0	20,5
154	gi|449687222	PREDICTED: allograft inflammatory factor 1-like [Hydra vulgaris]	155	17334	3	1	3	1	29,3
155	gi|221126057	PREDICTED: antistasin-like [Hydra vulgaris]	154	26072	11	2	3	1	15,1
156	gi|831771570	hypothetical protein SAMD00019534_067750 [Acytostelium subglobosum LI	150	71282	5	0	4	0	6,1
157	gi|449661942	PREDICTED: arginase-1-like [Hydra vulgaris]	150	36923	4	1	3	1	10,3
158	gi|828213619	PREDICTED: gelsolin-like protein 1 [Hydra vulgaris]	147	40776	5	0	4	0	14,1
159	gi|828220786	PREDICTED: rho GDP-dissociation inhibitor 1-like [Hydra vulgaris]	147	22744	5	0	4	0	18,2
160	gi|828191663	PREDICTED: neogenin-like [Hydra vulgaris]	145	209395	4	0	4	0	2,8
161	gi|478520725	PREDICTED: keratin, type I cytoskeletal 26 [Ceratotherium simum simum]	144	52420	6	1	3	1	5,6
162	gi|449679776	PREDICTED: failed axon connections homolog [Hydra vulgaris]	143	51908	3	1	3	1	7,7
163	gi|83595133	manganese superoxide dismutase [Hydra vulgaris]	142	24334	4	0	3	0	12,8
164	gi|13111486	elongation factor-2, partial [Artemia salina]	141	71657	4	1	3	1	6,2
165	gi|221090861	PREDICTED: cathepsin L1-like [Hydra vulgaris]	141	36531	3	1	3	1	9,6
166	gi|526117746	flp protein [Hydra vulgaris]	141	14995	3	0	3	0	26,2
167	gi|701307518	PREDICTED: LOW QUALITY PROTEIN: keratin, type II cytoskeletal cochleal-like [	139	54856	5	0	4	0	6,8
168	gi|221120289	PREDICTED: uncharacterized protein LOC100199298 isoform X1 [Hydra vulgar	138	18479	3	0	3	0	17,6
169	gi|449666332	PREDICTED: zinc metalloproteinase nas-6-like [Hydra vulgaris]	136	33291	4	0	4	0	16
170	gi|526117401	PPOD2 peroxidase-like precursor [Hydra vulgaris]	135	32661	2	1	2	1	8
171	gi|449667021	PREDICTED: zinc metalloproteinase nas-14-like [Hydra vulgaris]	135	20331	3	0	3	0	16,6
172	gi|76560210	elongation factor 1 alpha [Colletes halophilus]	135	8539	3	0	3	0	25
173	gi|221091838	PREDICTED: calcyphosin-like protein [Hydra vulgaris]	134	21273	3	1	3	1	14,7
174	gi|221124062	PREDICTED: heme-binding protein 1-like [Hydra vulgaris]	134	29921	5	0	4	0	14,8
175	gi|828204847	PREDICTED: urocanate hydratase-like [Hydra vulgaris]	134	75467	3	1	3	1	4,9
176	gi|526117507	kazal-type serine protease inihibitor 2 precursor [Hydra vulgaris]	134	19400	5	0	4	0	23,2
177	gi|221109761	PREDICTED: uncharacterized protein LOC100200582 [Hydra vulgaris]	131	21912	3	0	3	0	18,5
178	gi|110433182	heat shock protein [Bursaphelenchus xylophilus]	130	70411	4	0	4	0	6,2
179	gi|114389	RecName: Full=Sodium/potassium-transporting ATPase subunit beta; AltName	129	36274	4	0	3	0	8,6
180	gi|828232304	PREDICTED: blastula protease 10-like [Hydra vulgaris]	129	27019	3	0	3	0	12,6
181	gi|221123276	PREDICTED: omega-amidase NIT2-like [Hydra vulgaris]	129	30745	4	0	4	0	16,2
182	gi|828195143	PREDICTED: pre-mRNA-processing factor 19-like [Hydra vulgaris]	127	55883	3	0	3	0	6,3
183	gi|828194138	PREDICTED: dystonin-like, partial [Hydra vulgaris]	123	381036	3	0	3	0	0,8
184	gi|828224045	PREDICTED: uncharacterized protein LOC100212316 [Hydra vulgaris]	122	91458	4	0	4	0	4,3
185	gi|449686817	PREDICTED: 40S ribosomal protein S12-like [Hydra vulgaris]	121	16472	2	1	2	1	14,3
186	gi|449679956	PREDICTED: gamma-aminobutyric acid receptor-associated protein-like 2 [Hyc	120	13699	4	0	4	0	26,3
187	gi|828227420	PREDICTED: LOW QUALITY PROTEIN: asparagine--tRNA ligase, cytoplasmic-like	120	64826	3	0	2	0	4,8
188	gi|15072473	peroxidase ppod2 [Hydra vulgaris]	119	33192	3	1	2	1	8,5
189	gi|742871900	hypothetical protein [Halocynthiibacter sp. PAMC 20958]	116	50662	2	0	2	0	2

190	gi|559183818	Heat shock protein 70, partial [Giardia intestinalis]	116	48333	3	0	3	0	4,6
191	gi|449671399	PREDICTED: actin-related protein 2/3 complex subunit 2-like isoform X2 [Hydr	115	34480	2	1	2	1	9,5
192	gi|828201578	PREDICTED: purine nucleoside phosphorylase-like, partial [Hydra vulgaris]	115	28573	2	1	2	1	9,9
193	gi|828227874	PREDICTED: uncharacterized protein LOC105848518, partial [Hydra vulgaris]	115	14191	4	0	4	0	29,7
194	gi|828224366	PREDICTED: xaa-Pro dipeptidase-like [Hydra vulgaris]	114	55873	3	0	3	0	5,3
195	gi|828225026	PREDICTED: peptidase M20 domain-containing protein 2-like [Hydra vulgaris]	113	58925	3	0	3	0	4,9
196	gi|449689149	PREDICTED: uncharacterized protein LOC100208770, partial [Hydra vulgaris]	113	23100	3	0	3	0	15,7
197	gi|828195701	PREDICTED: kynurenine--oxoglutarate transaminase-like [Hydra vulgaris]	112	21895	1	1	1	1	10,7
198	gi|449670322	PREDICTED: dipeptidyl peptidase 1-like [Hydra vulgaris]	112	51835	3	0	3	0	7
199	gi|221111809	PREDICTED: carbonic anhydrase 2-like [Hydra vulgaris]	110	34574	3	0	3	0	7,7
200	gi|221113581	PREDICTED: protein DEK-like [Hydra vulgaris]	109	41907	3	0	3	0	7,3
201	gi|221130733	PREDICTED: astacin-like metalloprotease toxin 5 [Hydra vulgaris]	108	27816	2	1	2	1	9,7
202	gi|828197209	PREDICTED: annexin A4-like [Hydra vulgaris]	106	66184	3	1	3	1	4,4
203	gi|828211213	PREDICTED: heterogeneous nuclear ribonucleoprotein A/B-like [Hydra vulgaris	106	19419	2	1	2	1	14,8
204	gi|828197199	PREDICTED: uncharacterized protein LOC101236102 [Hydra vulgaris]	106	66420	4	0	3	0	5,9
205	gi|510863409	peptidyl-prolyl cis-trans isomerase, cyclophilin-type [Ancylostoma ceylanicum	105	16883	8	0	3	0	9,7
206	gi|221121520	PREDICTED: agmatinase, mitochondrial-like [Hydra vulgaris]	105	34037	2	1	2	1	9,1
207	gi|449670247	PREDICTED: probable inactive purple acid phosphatase 2 [Hydra vulgaris]	104	67196	2	1	2	1	3,4
208	gi|828218463	PREDICTED: uncharacterized protein LOC101241534 [Hydra vulgaris]	103	142054	3	0	3	0	2,5
209	gi|449678564	PREDICTED: lysosome-associated membrane glycoprotein 1-like [Hydra vulgari	103	21879	2	0	2	0	10,7
210	gi|221131162	PREDICTED: ATP synthase subunit alpha, mitochondrial [Hydra vulgaris]	103	59027	3	0	3	0	6,2
211	gi|449665331	PREDICTED: contactin-associated protein-like 5 [Hydra vulgaris]	102	136592	3	0	3	0	3,2
212	gi|46909251	ATP synthase beta subunit, partial [Obelia sp. KJP-2004]	102	46198	2	0	2	0	7,3
213	gi|828195674	PREDICTED: gamma-glutamyltranspeptidase 1-like [Hydra vulgaris]	102	67827	3	0	3	0	7,5
214	gi|526117489	kazal-type serine protease inihibitor 3 precursor [Hydra vulgaris]	102	19438	5	0	3	0	19
215	gi|221131289	PREDICTED: methylmalonyl-CoA epimerase, mitochondrial-like [Hydra vulgari	101	18258	2	0	2	0	18,8
216	gi|828196768	PREDICTED: calsequestrin-2-like [Hydra vulgaris]	101	46695	3	0	3	0	9,2
217	gi|221120850	PREDICTED: dihydropteridine reductase-like [Hydra vulgaris]	100	25220	3	1	2	1	11,9
218	gi|526117377	caspase 7 [Hydra vulgaris]	100	47366	3	0	3	0	7,4
219	gi|449664868	PREDICTED: threonine--tRNA ligase, cytoplasmic-like isoform X1 [Hydra vulgar	100	83661	3	0	3	0	3,6
220	gi|449676841	PREDICTED: putative acyl-coenzyme A oxidase 3.2, peroxisomal [Hydra vulgari	100	72300	3	0	3	0	5,1
221	gi|828190890	PREDICTED: 60S acidic ribosomal protein P2-like [Hydra vulgaris]	99	11782	3	1	2	1	21,6
222	gi|449689337	PREDICTED: uncharacterized protein LOC100212684, partial [Hydra vulgaris]	99	24278	1	1	1	1	8,2
223	gi|221122769	PREDICTED: uncharacterized protein LOC100209607 [Hydra vulgaris]	98	25274	3	1	2	1	13,8
224	gi|538775593	piwi-like protein HyWI [Hydra vulgaris]	98	101873	3	0	3	0	2,8
225	gi|449680876	PREDICTED: endochitinase 4-like [Hydra vulgaris]	98	52510	2	0	2	0	5,8
226	gi|156402513	predicted protein [Nematostella vectensis]	97	21970	2	0	2	0	9,5
227	gi|221121832	PREDICTED: multiple inositol polyphosphate phosphatase 1-like [Hydra vulgar	96	50712	2	0	2	0	6
228	gi|221126625	PREDICTED: polypyrimidine tract-binding protein 1-like [Hydra vulgaris]	96	56616	1	1	1	1	3,2
229	gi|449680926	PREDICTED: nuclear transport factor 2-like [Hydra vulgaris]	95	13926	2	0	2	0	17,6
230	gi|440200331	triosephosphate isomerase, partial [Odites leucostola]	94	16181	2	0	2	0	13,5
231	gi|17137630	cytoplasmic dynein light chain 2, isoform A [Drosophila melanogaster]	94	10465	2	0	2	0	24,7
232	gi|449687420	PREDICTED: uncharacterized protein LOC100205832 isoform X1 [Hydra vulga	93	24618	4	0	2	0	8,4
233	gi|221123418	PREDICTED: eukaryotic translation initiation factor 4H-like [Hydra vulgaris]	93	30810	1	1	1	1	5,8
234	gi|828214768	PREDICTED: delta-1-pyrroline-5-carboxylate dehydrogenase, mitochondrial-lil	93	40600	3	0	3	0	10,6
235	gi|449680259	PREDICTED: glutathione peroxidase 2-like [Hydra vulgaris]	92	23459	3	0	3	0	13,8
236	gi|449687197	PREDICTED: golgi-associated plant pathogenesis-related protein 1-like [Hydra v	92	29150	3	0	2	0	8,6
237	gi|828204323	PREDICTED: hemicentin-2-like isoform X1 [Hydra vulgaris]	92	158275	2	0	2	0	1,6

238	gi|449684745	PREDICTED: glutathione S-transferase-like [Hydra vulgaris]	90	24182	2	0	2	0	11,4
239	gi|497899026	MULTISPECIES: hypothetical protein [Pseudomonas]	90	25538	2	0	2	0	8
240	gi|449666254	PREDICTED: glutamic acid-rich protein-like [Hydra vulgaris]	90	44127	1	1	1	1	3,9
241	gi|449691823	PREDICTED: dipeptidyl peptidase 3-like, partial [Hydra vulgaris]	89	33965	2	0	2	0	7,7
242	gi|221132768	PREDICTED: RNA polymerase II transcriptional coactivator-like [Hydra vulgaris	89	13128	3	0	3	0	15,7
243	gi|221114177	PREDICTED: alpha-crystallin A chain-like [Hydra vulgaris]	88	26553	2	0	2	0	9,4
244	gi|828192317	PREDICTED: peptidyl-prolyl cis-trans isomerase H-like [Hydra vulgaris]	87	17635	2	0	2	0	14,5
245	gi|449692187	PREDICTED: purine nucleoside phosphorylase-like, partial [Hydra vulgaris]	87	16840	2	0	2	0	12,8
246	gi|501292293	myosin heavy chain [Riptortus pedestris]	87	167897	2	0	2	0	1,4
247	gi|493710903	hypothetical protein [Providencia alcalifaciens]	87	14905	2	0	2	0	10,8
248	gi|551617245	hypothetical protein EMIHUDRAFT_423268 [Emiliania huxleyi CCMP1516]	87	56358	2	0	2	0	3,2
249	gi|449682262	PREDICTED: zinc metalloproteinase nas-13-like [Hydra vulgaris]	87	58069	3	0	3	0	6
250	gi|221113277	PREDICTED: uncharacterized protein LOC100214198 [Hydra vulgaris]	86	30392	4	0	2	0	7,8
251	gi|828220298	PREDICTED: ena/VASP-like protein [Hydra vulgaris]	86	39847	3	0	2	0	3,7
252	gi|828206951	PREDICTED: uncharacterized protein LOC100208668 isoform X1 [Hydra vulgar	85	253566	3	0	2	0	2
253	gi|449689073	PREDICTED: ornithine aminotransferase, mitochondrial-like [Hydra vulgaris]	85	48365	2	0	2	0	7
254	gi|766939774	PREDICTED: triosephosphate isomerase [Ceratosolen solmsi marchali]	85	27658	3	0	3	0	7,7
255	gi|828222781	PREDICTED: hypoxanthine-guanine phosphoribosyltransferase-like [Hydra vulg	85	24651	2	0	2	0	9,7
256	gi|221125651	PREDICTED: small nuclear ribonucleoprotein Sm D1-like [Hydra vulgaris]	85	13631	2	0	2	0	24,2
257	gi|487975987	MULTISPECIES: SnoaL-like domain protein [Acinetobacter calcoaceticus/baum	85	20833	2	0	2	0	5,1
258	gi|685831842	Annexin family and Annexin repeat-containing protein [Strongyloides ratti]	84	36712	2	2	1	1	3,4
259	gi|828206370	PREDICTED: epidermal growth factor receptor substrate 15-like 1 [Hydra vulga	84	89358	2	0	2	0	2,6
260	gi|768420965	PREDICTED: peptidyl-prolyl cis-trans isomerase B [Plutella xylostella]	83	22092	3	0	2	0	9,9
261	gi|32532	unnamed protein product [Homo sapiens]	83	26932	1	1	1	1	5,3
262	gi|28194281	ubiquitin extension protein [Heterodera glycines]	82	11762	6	0	2	0	21
263	gi|221103804	PREDICTED: probable glutathione S-transferase 7 [Hydra vulgaris]	82	24521	1	1	1	1	7
264	gi|828217423	PREDICTED: glutamate decarboxylase 2-like isoform X1 [Hydra vulgaris]	82	61072	1	1	1	1	2,8
265	gi|449666836	PREDICTED: malectin-A-like [Hydra vulgaris]	81	32778	2	0	2	0	8,5
266	gi|828211197	PREDICTED: sorting nexin-32-like [Hydra vulgaris]	81	48466	2	0	2	0	5,4
267	gi|459367722	hypothetical protein G210_3086 [Candida maltosa Xu316]	80	74058	2	0	2	0	1,2
268	gi|899147016	peroxiredoxin 3 [Esox lucius]	80	27673	3	0	2	0	7,6
269	gi|828207749	PREDICTED: N-acyl-phosphatidylethanolamine-hydrolyzing phospholipase D-l	80	52007	2	0	2	0	3,8
270	gi|828212124	PREDICTED: MAM and LDL-receptor class A domain-containing protein 1-like [	79	770950	2	0	2	0	0,3
271	gi|828192461	PREDICTED: aspartyl aminopeptidase-like [Hydra vulgaris]	79	51537	2	0	2	0	4,4
272	gi|999604	Chain A, Crystallographic Studies On A Family Of Cellular Lipophilic Transport	79	14923	2	1	1	1	9,2
273	gi|294882533	Metal homeostasis factor ATX1, putative [Perkinsus marinus ATCC 50983]	78	7429	1	1	1	1	17,6
274	gi|449679798	PREDICTED: U1 small nuclear ribonucleoprotein A-like [Hydra vulgaris]	78	25255	3	2	1	1	5,9
275	gi|890692673	GntR family transcriptional regulator [Photobacterium swingsii]	78	25836	1	1	1	1	4
276	gi|828205429	PREDICTED: uncharacterized protein LOC100204510 [Hydra vulgaris]	77	134052	2	0	2	0	2,3
277	gi|221126681	PREDICTED: prefoldin subunit 5-like [Hydra vulgaris]	77	18675	1	1	1	1	7,7
278	gi|449663959	PREDICTED: acylpyruvase FAHD1, mitochondrial-like [Hydra vulgaris]	76	23816	2	0	2	0	14,9
279	gi|443702382	hypothetical protein CAPTEDRAFT_177200 [Capitella teleta]	76	23316	1	1	1	1	6
280	gi|449662629	PREDICTED: iodotyrosine dehalogenase 1-like [Hydra vulgaris]	76	31350	2	0	2	0	10,7
281	gi|828197526	PREDICTED: malate dehydrogenase, cytoplasmic-like [Hydra vulgaris]	76	37041	3	0	3	0	9,6
282	gi|221123857	PREDICTED: lactoylglutathione lyase-like [Hydra vulgaris]	75	20110	1	1	1	1	11
283	gi|221131112	PREDICTED: prefoldin subunit 6-like [Hydra vulgaris]	75	15339	2	0	2	0	21,5
284	gi|553309896	polysaccharide pyruvyl transferase CsaB [Peptoniphilus sp. BV3C26]	75	41687	3	0	2	0	3,8
285	gi|221130032	PREDICTED: isochorismatase domain-containing protein 2, mitochondrial-like	75	22754	1	1	1	1	6,5

286	gi|449679397	PREDICTED: uncharacterized protein LOC100197967 [Hydra vulgaris]	75	40545	3	0	2	0	4,2
287	gi|449671578	PREDICTED: S-crystallin 4-like [Hydra vulgaris]	74	23979	2	0	2	0	7,7
288	gi|20145612	putative actin, partial [Hydractinia echinata]	74	8589	3	0	3	0	49,4
289	gi|828225023	PREDICTED: peptidase M20 domain-containing protein 2-like, partial [Hydra v	74	34646	3	0	3	0	7,5
290	gi|156336944	hypothetical protein NEMVEDRAFT_v1g150407 [Nematostella vectensis]	74	7062	2	0	2	0	29,7
291	gi|165979176	Cu-Zn superoxide dismutase, partial [Rhizophagus proliferus]	73	12709	1	1	1	1	8,3
292	gi|221124690	PREDICTED: endoplasmin-like [Hydra vulgaris]	71	94322	2	0	2	0	2,4
293	gi|828235608	PREDICTED: cytosol aminopeptidase-like [Hydra vulgaris]	70	56366	2	0	2	0	4,1
294	gi|330796823	replication factor C subunit [Dictyostelium purpureum]	70	55856	2	0	2	0	3
295	gi|828203837	PREDICTED: dimethylglycine dehydrogenase, mitochondrial-like [Hydra vulgar	70	96756	2	0	2	0	2,6
296	gi|221102513	PREDICTED: S-adenosylmethionine synthase isoform type-1-like [Hydra vulgari	69	42626	1	1	1	1	5,7
297	gi|828194686	PREDICTED: alpha-mannosidase 2-like [Hydra vulgaris]	69	130659	2	0	2	0	1,8
298	gi|5596622	isovaleryl-CoA-dehydrogenase precursor [Arabidopsis thaliana]	69	45371	1	1	1	1	2,9
299	gi|674263667	glutathione s transferase mu [Echinococcus multilocularis]	69	25815	3	0	2	0	4,1
300	gi|828202993	PREDICTED: prelamin-A/C-like [Hydra vulgaris]	68	68122	3	0	2	0	3,1
301	gi|490068496	MULTISPECIES: hypothetical protein [Streptomyces]	68	17530	2	1	1	1	7,6
302	gi|637068998	replication initiation protein [Streptococcus suis]	68	48271	2	0	2	0	3,2
303	gi|828195809	PREDICTED: astacin-like metalloprotease toxin 5 [Hydra vulgaris]	67	28949	2	0	2	0	7,1
304	gi|221131677	PREDICTED: cytochrome b-c1 complex subunit Rieske, mitochondrial-like [Hy	67	29671	3	0	2	0	4,1
305	gi|828226794	PREDICTED: superoxide dismutase [Cu-Zn]-like [Hydra vulgaris]	67	21118	1	1	1	1	6,3
306	gi|221102622	PREDICTED: lactadherin-like [Hydra vulgaris]	67	21699	2	0	2	0	10,2
307	gi|83595137	mitochondrial phospholipid hydroperoxide glutathione peroxidase [Hydra vu	67	21685	3	0	2	0	14,2
308	gi|493622316	REX family transcriptional regulator [Pseudoflavonifractor capillosus]	67	23378	1	1	1	1	3,7
309	gi|449683356	PREDICTED: proliferation-associated protein 2G4-like [Hydra vulgaris]	67	45036	2	0	2	0	5,5
310	gi|828231348	PREDICTED: LOW QUALITY PROTEIN: transmembrane 9 superfamily member 2-	67	76636	2	0	2	0	2,9
311	gi|88942082	superoxide dismutase [Azumapecten farreri]	66	15764	2	0	1	0	9,8
312	gi|828199555	PREDICTED: microtubule-actin cross-linking factor 1-like, partial [Hydra vulgar	66	777032	1	0	1	0	0,2
313	gi|221108650	PREDICTED: serpin B6-like [Hydra vulgaris]	65	43560	2	0	2	0	8,7
314	gi|449676978	PREDICTED: heterogeneous nuclear ribonucleoprotein Q-like [Hydra vulgaris]	65	68606	3	0	2	0	3,4
315	gi|828204809	PREDICTED: branched-chain-amino-acid aminotransferase, cytosolic-like [Hyd	65	38516	2	0	2	0	5,3
316	gi|828209870	PREDICTED: hydroxyacid-oxoacid transhydrogenase, mitochondrial-like [Hydr	65	51491	2	0	2	0	4,2
