# Supplementary material for: The Wnt-specific astacin proteinase HAS-7 restricts head organizer formation in Hydra
Source: BMC Biol. 2021 Jun 9;19:120. doi: 10.1186/s12915-021-01046-9 (PMC8191133; doi:10.1186/s12915-021-01046-9)

Uncropped Western Blot and gel images shown in Fig. 1b

HyWnt3-His/tentacle lysate

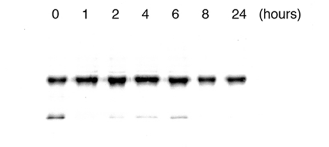

HyWnt3-His/head lysate

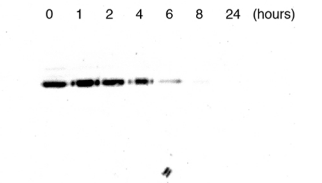

HyWnt3-His/upper body column lysate

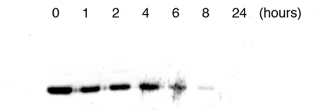

HyWnt3-His/lower body column lysate

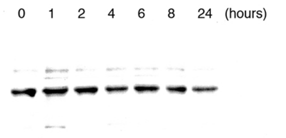

HyWnt3-His/PBS

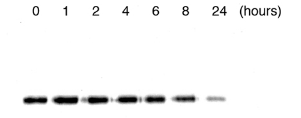

HyWnt3-His/head lysate/200 mM EDTA

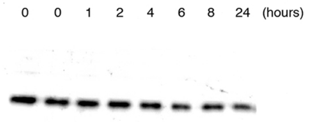

HyWnt3-His/head lysate/200 mM Phenantrolin

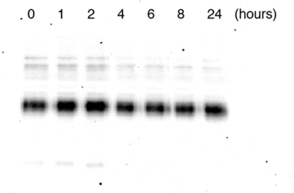

HyWnt3-His/head lysate/200 mM Batimastat

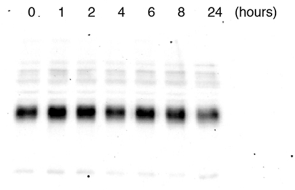

BSA Coomassie staining

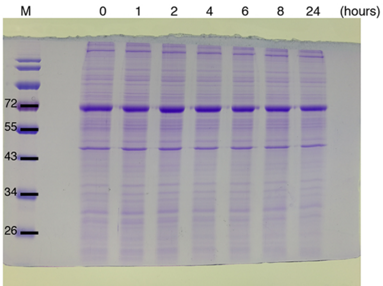

Uncropped Western Blot images shown in Fig. 1c-d

HyDkk1/2/4-His/tentacle lysate

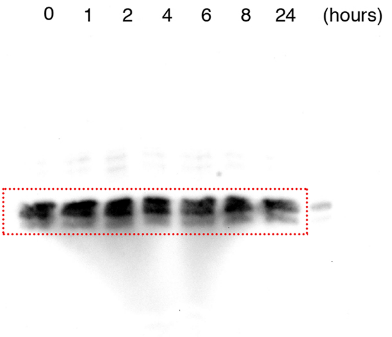

HyDkk1/2/4-His/head lysate

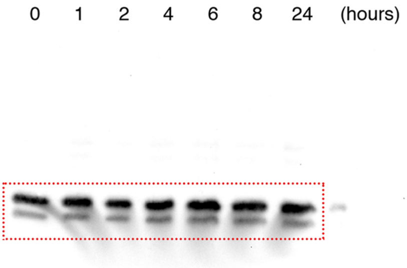

HyDkk1/2/4-His/upper body column lysate

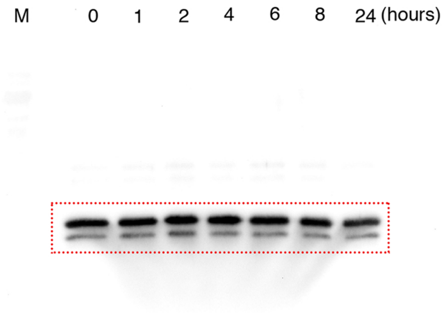

HyDkk1/2/4-His/lower body column lysate

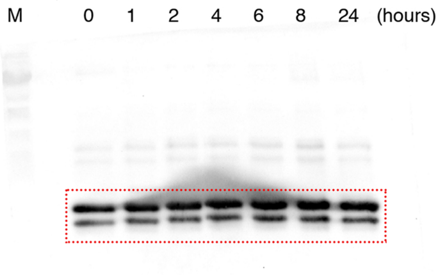

α-Tubulin in body part lysates

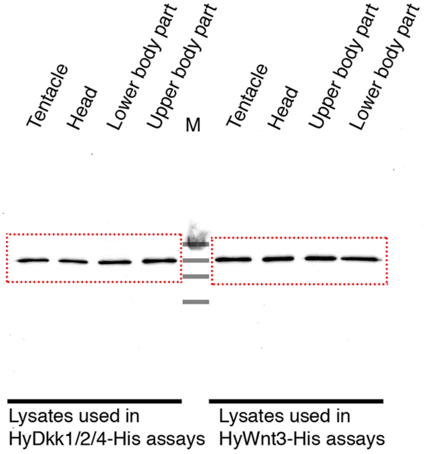

Uncropped Western Blot images shown in Fig. 1f

HyWnt3-His/head lysate

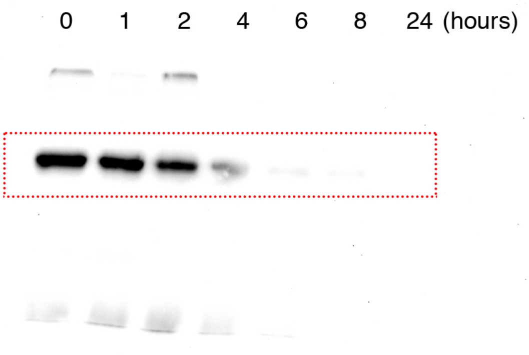

HyWnt3-His/head lysate/0.5 mM Fetuin-B

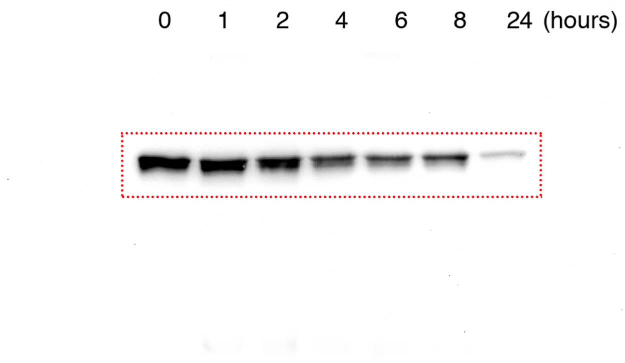

HyWnt3-His/head lysate/1 mM Fetuin-B

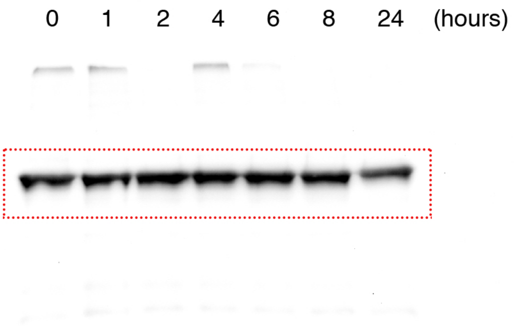

α-Tubulin detection in head lysate used for Fetuin-B assays

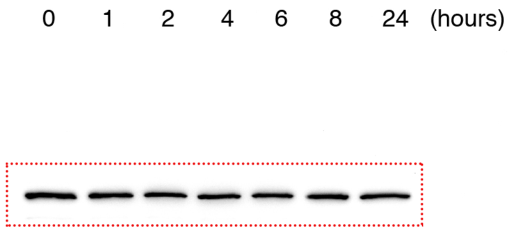

Uncropped Western Blot images shown in Fig. 3a-d

Fig. 3a, anti-HAS-7

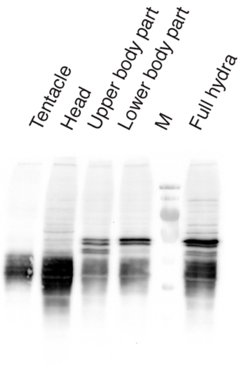

Fig. 3b

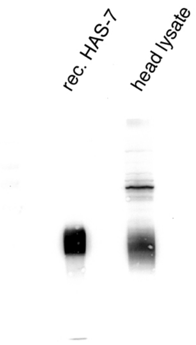

Fig. 3c, anti-HAS-7

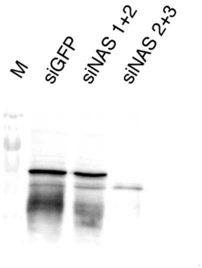

Fig. 3d, siGFP

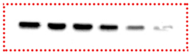

Fig. 3d, siGFP, Tubulin

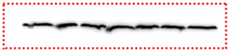

Fig. 3a, anti-Tubulin

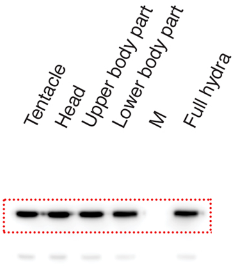

Fig. 3c, anti-Tubulin

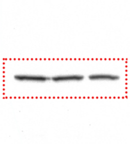

Fig. 3d, siHAS-7

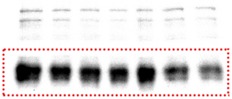

Fig. 3d, siHAS-7, Tubulin

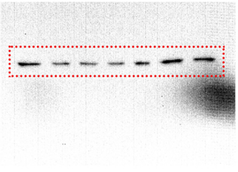

Uncropped Western Blot images shown in Fig. S1b

Fig. S1b, HyWnt3

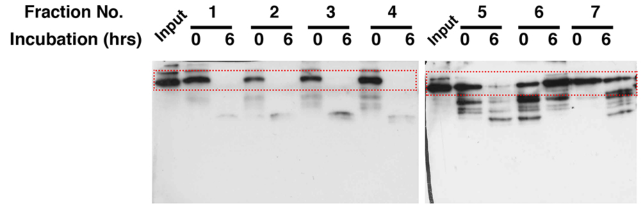

Fig. S1b, HmCadherin

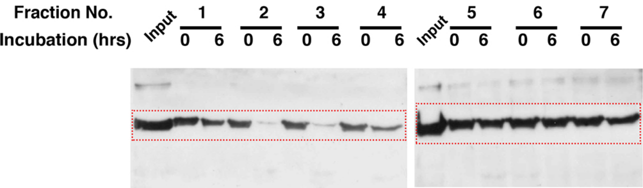

Uncropped Western Blot images shown in Fig. S4

Fig. S4a

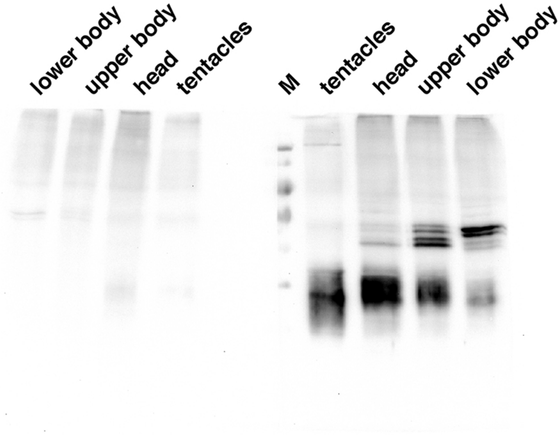

Fig. S4b

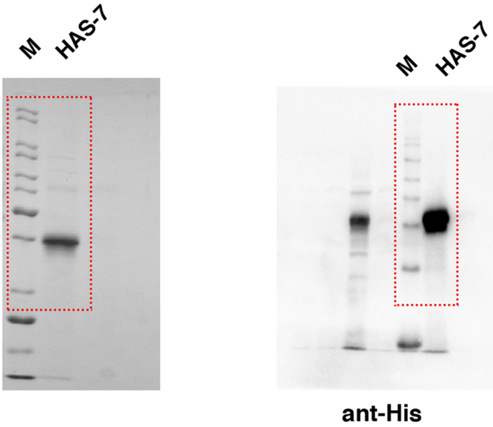

Fig. S4c

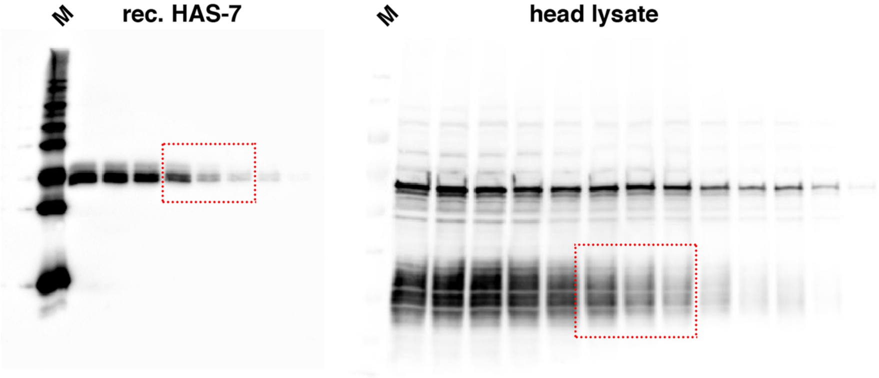

Fig. S4d

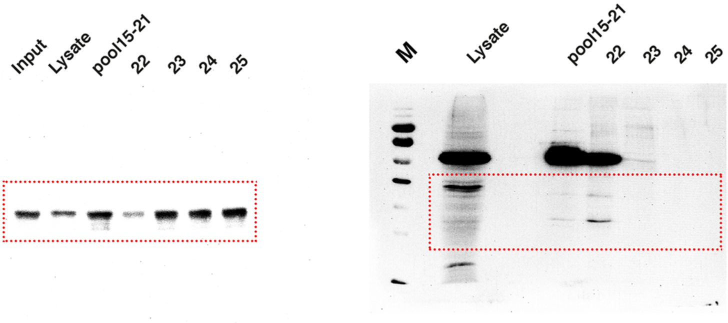

Supplement: Supplementary file 11 — Additional file 11: Fig. S9. Uncropped Western blot and gel images [file 12915_2021_1046_MOESM11_ESM.pdf]
