# Supplementary material for: The Wnt-specific astacin proteinase HAS-7 restricts head organizer formation in Hydra
Source: BMC Biol. 2021 Jun 9;19:120. doi: 10.1186/s12915-021-01046-9 (PMC8191133; doi:10.1186/s12915-021-01046-9)
Supplement: Supplementary file 12 — Additional file 12: Table S3. LNA and RNA probe sequences used for WISH. [file 12915_2021_1046_MOESM12_ESM.docx]

**Supplementary Table 3.** LNA and RNA probe sequences used for WISH.

| **Gene name** | **Accession Number** | **LNA or in situ probe sequence** |
| --- | --- | --- |
| HAS-1 | XP_012565441.1 | Full length antisense mRNA (1-717) |
| HAS-2 | XP_002162822.1 | ATCACGGTCAGGACGGCATTGT |
| HAS-3 | XP_002166229.3 | TAGTGACATATCTATCTCTGT |
| HAS-4 | XP_002162738.1 | ATTGTTCAGGTGTCAATTGTA |
| HAS-5 | XP_002164800.1 | TCAGACAAGTGTAGGTGTGATA |
| HAS-6 | XP_002157397.2 | TCTAAGGCAAGTGTAAGTGTGA |
| HAS-7 | XP_012560086.1 | Full length antisense mRNA (1-1021) |
| HAS-8 | XP_002153855.1 | TATGACGTAAGGTACAACAGCA |
| HAS-9 | XP_002161766.1 | ACGGCAAGATCTGCGGCAAGAT |
| HAS-10 | XP_002159980.2 | TACTGTACCAAGTCGCAAGCAA |
| HAS-11 | XP_012561076.1 | ACATGACTTGCAGCATAGCTGA |
| HMP-1 | NP_001296695.1 | Full length antisense mRNA (1-858) |
