# Supplementary material for: The Wnt-specific astacin proteinase HAS-7 restricts head organizer formation in Hydra
Source: BMC Biol. 2021 Jun 9;19:120. doi: 10.1186/s12915-021-01046-9 (PMC8191133; doi:10.1186/s12915-021-01046-9)
Supplement: Supplementary file 13 — Additional file 13: Table S4. siRNA and qPCR primer sequences. [file 12915_2021_1046_MOESM13_ESM.docx]

**Table S4.** siRNA and qPCR primer sequences.

| **Gene name** | **siRNA Sequence (anti-sense)** |
| --- | --- |
| HAS-7 | GUCUCCUUCAAACAGAUUGUUUU (siRNA1)  AAUGUUAUUCCAUAUAAUAUUUU (siRNA2)  UGAUUUGCAAUAACCUGUAUUUU (siRNA3) |
| HMP1 | AAUCACUGCAGAUAUGUAUGCUUUU (siRNA1)  UCCAGUGACACCGCUACACUUUU (siRNA2) |
| HAS-1 | AAAAUGUUAUUCCAUAUAAUAUUUU (siRNA1)  AAUGAUUUGCAAUAACCUGUAUUUU (siRNA2) |
| HyDkk-1/2/4 | CGAACGACGUCUAACCAGCUUUU (siRNA1)  AAACGGUUUGCAAAUGAAGCCAUUUU (siRNA2) |
| GFP | AAUUGGCCAUGGAACAGGUAGUUUU |
| Scrambled GFP | AAACCGGUGUGAAUCGAUGAGUUUU |
| HyWnt3 | CUUUGUAUAGAAACUCCAUUUUU |
| HyWnt8 | GUCUCUCCGGAUGUGGUUAUU (siRNA1)  AGCAAUCAAGACGAAUCGUUU (siRNA2) |
| HyNdr | UCGUAGAAAUAUUGUUUCCUUUU (siRNA1)  UGUUACUUCAAAUGUAAUCCAUU (siRNA2) |
| Hyβ-catenin | AAGGUUAUGAUGAUGAUGAAGUU |
|  |  |
|  | **qPCR Primer Sequence** |

EF1alpha Fw: TATTGATAGACCTTTTCGACTTTGC

Rev: CTGTACAGAGCCACTTTCAACTTTT

HAS-7 Fw: GGATGTGAAATCAAATGGTTATGCT

Rev: TGATGAACTCATTCTTCGAAGATCG
